# Time-resolved comparative molecular evolution of oxygenic photosynthesis

**DOI:** 10.1016/j.bbabio.2021.148400

**Published:** 2021-06-01

**Authors:** Thomas Oliver, Patricia Sánchez-Baracaldo, Anthony W. Larkum, A. William Rutherford, Tanai Cardona

**Affiliations:** a Department of Life Sciences, Imperial College London, London, UK; b School of Geographical Sciences, University of Bristol, Bristol, UK; c University of Technology Sydney, Ultimo, NSW, Australia

**Keywords:** MRCA, most recent common ancestor, LUCA, the last universal common ancestor or the most recent common ancestor of life, MSV, Margulisbacteria, Sericytochromatia and Vampirovibrionia, PSII, photosystem II, PSI, photosystem I, CBP, chlorophyll-binding protein, RC, reaction centre, ROS, reactive oxygen species, GOE, Great Oxidation Event, *d*D0, duplication leading to the origin of D1 and D2 subunits of photosystem II, *d*CP, duplication leading to the origin of CP43 and CP47 subunits of photosystem II, *d*AB, duplication leading to the origin of Alpha and Beta subunits of ATP synthase, ΔT, the span of time between dD0, dCP, dAB, or the LUCA, and the MRCA of Cyanobacteria, Ma, Mega-annum, million years, Ga, Giga-annum, billion years, ν, Rate of amino acid substitutions per site, δ Ga^−1^, Amino acid substitutions per site per billion years, Origin of photosynthesis, Origin of life, Cyanobacteria, Photosystem, Reaction centre, Water oxidation

## Abstract

Oxygenic photosynthesis starts with the oxidation of water to O_2_, a light-driven reaction catalysed by photosystem II. Cyanobacteria are the only prokaryotes capable of water oxidation and therefore, it is assumed that the origin of oxygenic photosynthesis is a late innovation relative to the origin of life and bioenergetics. However, when exactly water oxidation originated remains an unanswered question. Here we use phylogenetic analysis to study a gene duplication event that is unique to photosystem II: the duplication that led to the evolution of the core antenna subunits CP43 and CP47. We compare the changes in the rates of evolution of this duplication with those of some of the oldest well-described events in the history of life: namely, the duplication leading to the Alpha and Beta subunits of the catalytic head of ATP synthase, and the divergence of archaeal and bacterial RNA polymerases and ribosomes. We also compare it with more recent events such as the duplication of Cyanobacteria-specific FtsH metalloprotease subunits and the radiation leading to Margulisbacteria, Sericytochromatia, Vampirovibrionia, and other clades containing anoxygenic phototrophs. We demonstrate that the ancestral core duplication of photosystem II exhibits patterns in the rates of protein evolution through geological time that are nearly identical to those of the ATP synthase, RNA polymerase, or the ribosome. Furthermore, we use ancestral sequence reconstruction in combination with comparative structural biology of photosystem subunits, to provide additional evidence supporting the premise that water oxidation had originated before the ancestral core duplications. Our work suggests that photosynthetic water oxidation originated closer to the origin of life and bioenergetics than can be documented based on phylogenetic or phylogenomic species trees alone.

## Highlights

• The origin of oxygenic photosynthesis cannot be located on a species tree.
• Vampirovibrionia, Sericytochromatia and Margulisbacteria lost photosynthesis.
• Photosystem II can be as old as the oldest enzymes.
• Earliest type II reaction centres were structurally like water-splitting Photosystem II.
• Anoxygenic photosynthesis is not more primitive than oxygenic photosynthesis.

## 1 Introduction

### 1.1 Evolution of Cyanobacteria

The origin of oxygenic photosynthesis is considered a turning point in the history of life, marking the transition from the ancient world of anaerobes into a productive aerobic world that permitted the emergence of complex life [1]. Oxygenic photosynthesis starts with photosystem II (PSII), the water-oxidizing and O_2_-evolving enzyme of Cyanobacteria and photosynthetic eukaryotes. PSII is a highly conserved, multicomponent, membrane protein complex, which was inherited by the *most recent common ancestor* (MRCA) of Cyanobacteria in a form that is structurally and functionally similar to that found in all extant species [2]. Thus, the origin of oxygenic photosynthesis antedates the MRCA of Cyanobacteria by an undetermined amount of time.

Cyanobacteria's closest living relatives are the clades known as Vampirovibrionia (formerly Melainabacteria) [3,4], followed by Sericytochromatia [5] and Margulisbacteria [6]. Described Margulisbacteria include intracellular symbionts of placozoans [7] or are found as part of a chain of complex symbiotic interactions in the gut of termites [8]. Vampirovibrionia also include gut symbionts as well as specialist predators of green algae [9]. Less is known of the Sericytochromatia [4]. Currently, no photosynthetic strains have been described in these groups of uncultured bacteria and this has led to the hypothesis that oxygenic photosynthesis arose during the time spanning the divergence of Vampirovibrionia and the MRCA of Cyanobacteria, starting from an entirely non-photosynthetic ancestral state [5,10]. Recent molecular clock studies have suggested that the span of time between the divergence of Cyanobacteria and Vampirovibrionia could be of several hundred million years [11,12]. However, it is still unclear from molecular clock analyses and the microfossil record when exactly the MRCA of Cyanobacteria occurred [13].

### 1.2 Evolution of photosystem II

The heart of PSII is made up of a heterodimeric reaction centre (RC) *core* coupled to a core *antenna*. The two subunits of the RC core of PSII are known as D1 and D2, and these are associated respectively with the antenna subunits known as CP43 and CP47. D1 and CP43 make up one monomeric half of the RC, and D2 and CP47, the other half. Water oxidation is catalysed by a Mn_4_CaO_5_ cluster coordinated by ligands from both D1 and CP43 [14,15]. The cluster is functionally coupled to a redox active tyrosine-histidine pair (Y_Z_-H190) also located in D1, which relays electrons from Mn to the oxidized chlorophyll pigments of the RC during charge separation [16]. In a cycle of four consecutive light-driven charge separation events, O_2_ is released in the decomposition of two water molecules.

Photosystems evolved first as homodimers [17,18]: therefore, the core and the antenna of PSII originated from ancestral gene duplication events that antedated the MRCA of Cyanobacteria. In this way, CP43/D1 retain sequence and structural identity with CP47/D2. The conserved structural and functional traits between CP43/D1 and CP47/D2 suggest that the ancestral PSII homodimer—prior to the duplication events—was not only structurally similar to heterodimeric PSII, but also that it could have split water and evolved protective mechanisms against the formation of reactive oxygen species (ROS) [[18], [19], [20]].

In a previous study, we attempted to measure the span of time between the duplication that led to D1 and D2 (*d*D0) and a point that approximated the MRCA of Cyanobacteria: a period of time that we called Δ**T** [19]. We observed that the magnitude of ΔT can be very large, well over a billion years. Such a large ΔT suggested that the origin of a water-oxidizing PSII substantially antedated the MRCA of Cyanobacteria and highlighted that the evolution of the process, prior to this ancestor, was likely marked by loss of photosynthesis and extinction events. This implies that despite their specialized heterotrophic lifestyles, an ancestral photosynthetic state at the divergence of Margulisbacteria, Sericytochromatia, and Vampirovibrionia (MSV) cannot be ruled out. However, our study neither provided an absolute age for the MRCA of Cyanobacteria nor the duplication event itself, as we simulated a comprehensive range of scenarios. Instead, we showed that even when the time-span of ΔT is over a billion years, the rate of protein evolution at the duplication point (*d*D0) needed to be over 40 times greater than any rate ever observed for D1 and D2, which decreased exponentially during the Archean and stabilized at current rates during the Proterozoic. Thus, the smaller the value of ΔT, the faster the rate at *d*D0, with the rate increasing following a power law function and reaching unrealistic values even when ΔT is still in the order of several hundred million years [19]. It was unclear then if such patterns of molecular evolution were unique to the core subunits of PSII or whether other systems have experienced similar evolutionary trajectories.

Here, to help in understanding the evolution of oxygenic photosynthesis, Cyanobacteria and MSV as a function of time, we compared the age of duplication leading to the RC antenna subunits, CP43 and CP47, with several well-defined ancient and more recent events. These include the duplication of the core catalytic subunits of ATP synthase, a very ancient event generally accepted to have occurred before the last universal common ancestor (LUCA) [[21], [22], [23], [24], [25]]; and the evolution of RNA polymerase catalytic subunit β (RpoB) and ribosomal proteins, which are universally conserved and widely accepted to have originated before the LUCA [[26], [27], [28], [29]]. We further constrain our analysis using in silico ancestral sequence reconstruction of PSII and through strict structural and functional rationales. We show that the core subunits of PSII show patterns of molecular evolution that are usually associated with some of the oldest transitions in the evolution of life. We also show that all events leading from a primordial homodimeric photosystem to Cyanobacteria's heterodimeric PSII can be logically reconstructed.

## 2 Results

### 2.1 Phylogenetic overview

**Fig. 1:**
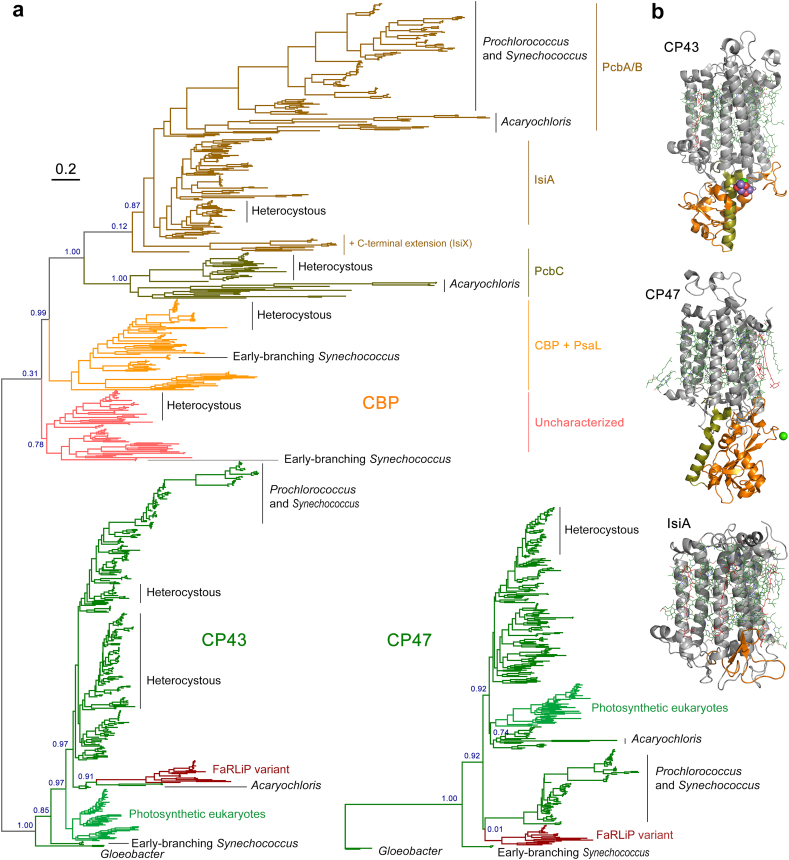
Maximum Likelihood trees of PSII core antenna subunits and derived light harvesting proteins. **a** A tree of CP43 and chlorophyll binding proteins (CBP). The unrooted tree splits into CP43 and CBP, with CP43 displaying a phylogeny roughly consistent with the evolution of Cyanobacteria, although several potential duplications of CP43 are noticeable within heterocystous Cyanobacteria and closer relatives. FaRLiP variant denotes the isoform used in the far-red light acclimation response. The CBP subtree shows a complex phylogeny driven by gene duplication events and fast evolution. The tree of CP47 was rooted at the divergence of *Gloeobacter* spp. Scale bar represents number of substitutions per site. **b** Structural comparison of CP43, CP47, and IsiA. The latter lacks the characteristic extrinsic loop (orange) of CP43 and CP47 that links the antenna with the electron donor side of PSII.

The phylogenies of CP43 and CP47 show that there is a much greater diversity of CP43 and CP43-derived subunits than CP47 (Fig. 1). This difference is the result of a greater number of gene duplication in CP43 than CP47 and mirrors the evolution of D1 compared with D2 [2], in which D1 has undergone more duplication events than D2. CP43 can be divided into two major groups: those that are assembled into PSII and can bind the Mn_4_CaO_5_ cluster, and those which have evolved to be used only as light harvesting complexes [30,31], known as chlorophyll binding proteins (CBP). The CBP are characterized by the loss of the extrinsic loop between the 5th and 6th transmembrane helices, where the ligands to the cluster are located (Fig. 1b). This large extrinsic loop is found in both CP43 and CP47 and interacts directly with the electron donor side of PSII, within D1 and D2 respectively. The unrooted tree of CP43 is consistent with CBP having a single origin likely occurring before the MRCA of Cyanobacteria (Supplementary Fig. S1) but have undergone an extensive duplication-driven diversification process. It also mirrors the evolution of D1 in that duplications appear to have occurred before the MRCA of Cyanobacteria [2]. The earliest of these D1 duplications also led to variants that lack the capacity to bind the Mn_4_CaO_5_ cluster [2,32], but are likely used in other supporting functions. Most notably, during chlorophyll *f* synthesis [33] in the far-red light acclimation response (FaRLiP) [34].

CP43 and CP47 are also distantly related to the antenna domain of cyanobacterial PSI and other type I RCs of phototrophic Chlorobia, Acidobacteriota and Heliobacteria (Supplementary Fig. S2). We found that the level of sequence identity between any two type I RC proteins is always greater than between a type I RC protein and CP43/CP47 (Supplementary Table S1). Therefore, the distance between CP43/CP47 and other type I antenna domains is the largest distance in the molecular evolution of RC proteins after that between type I core and type II RC core domains. The phylogenetic relationships between type I and type II RCs have been reviewed in detail before [20,35,36].

The phylogenetic distribution of Alpha and Beta subunits of the F-type ATP synthase showed that all Cyanobacteria have an F-type ATP synthase, and a fewer number of strains have an additional Na^+^-translocating ATPase (N-ATPase) of the bacterial F-type, as had been reported before [37]. We found that MSV have a standard F-type ATP synthase (Supplementary Fig. S3), but some N-ATPase Alpha and Beta sequences were also found in Vampirovibrionia and Sericytochromatia datasets, but not in Margulisbacteria. In this study we focused on the standard F-type ATP synthase of Cyanobacteria for further analysis.

We also constructed a phylogeny of bacterial RNA polymerase subunit β (RpoB) for divergence time estimation. This focused on Cyanobacteria and MSV, as well as phyla with known phototrophic representatives and included Thermotogae and Aquificae as potential outgroups (Supplementary Fig. S4). The tree was largely consistent with previous observed relationships between the selected groups [38], within Cyanobacteria and MSV, and within other phototrophs and their non-phototrophic relatives [39,40]. The only exception was Aquificae, which branched as a sister clade to Acidobacteriota, a feature that had been reported before for RpoB [28], and likely represents an ancient horizontal gene transfer event.

### 2.2 Rates of evolution

#### 2.2.1 Distances

**Fig. 2:**
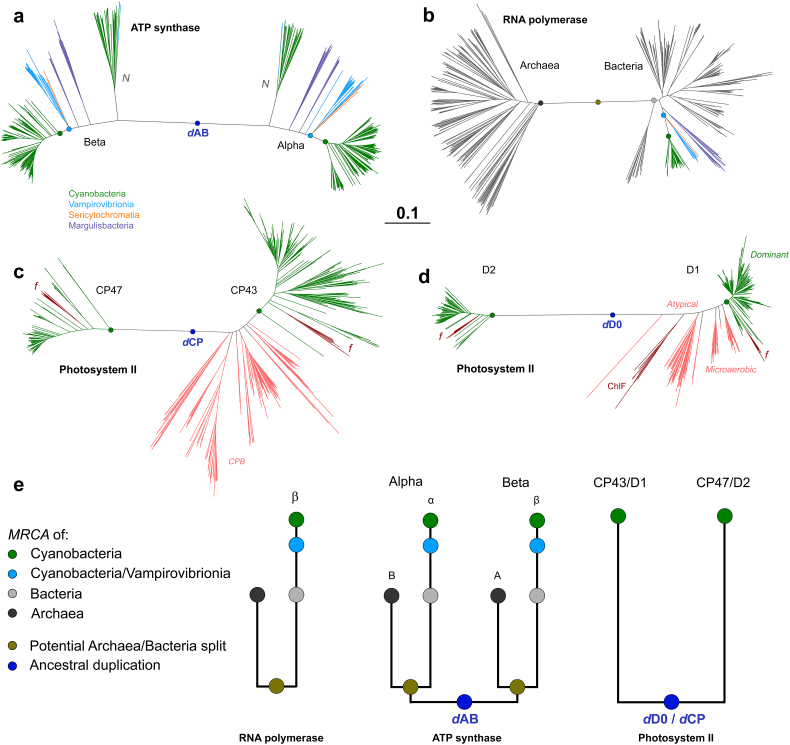
Distance comparison of the core subunits of ATP synthase, RNA polymerase, and PSII. **a** Alpha and Beta subunits of the F-type ATP synthase from Cyanobacteria, Vampirovibrionia, Sericytochromatia and Margulisbacteria. *N* denotes Na^+^ translocating N-type ATPase. The duplication event leading to Alpha and Beta is denoted as *d*AB. The green dot marks the MRCA of Cyanobacteria, and the light-blue dot the MRCA of the clade including Cyanobacteria and Vampirovibrionia. **b** Archaeal and Bacterial RpoB of RNA polymerase. **c** CP43 and CP47 subunits of PSII. The letter *f* denotes FaRLiP variants. The duplication event leading to CP43 and CP47 is denoted as *d*CP. Pink branches highlight the CBP subunits. **d** D1 and D2 of PSII. They show a pattern that mimics that of CP43 and CP47. Pink branches denote the atypical D1 forms and other variants that are thought to predate the MRCA of Cyanobacteria. The sequences marked with *dominant* represent the standard D1 form of PSII, inherited by the MRCA of Cyanobacteria and found in all oxygenic phototrophs [2,19]. ChlF marks the atypical D1 form involved in the synthesis of chlorophyll *f* during FaRLiP [33]. The duplication leading to D1 and D2 is denoted as *d*D0. **e** Schematic representation of distance and distribution of these enzymes in Cyanobacteria and relatives in relation to the MRCA of Bacteria, of Archaea, and the LUCA.

To gather temporal information, we compared the phylogenetic distances between CP43 and CP47, Alpha and Beta subunits of the cyanobacterial F-type ATP synthase, and archaeal and bacterial RpoB (visualized in Fig. 2, but see also Supplementary Figs. S1b, S3 and S4b). We found that the distances between Alpha and Beta, and the divergence of archaeal and bacterial RpoB, are very large relative to the distance between the divergence of Vampirovibrionia and Cyanobacteria. In the case of RpoB, the distance between Vampirovibrionia and Cyanobacteria is about a fifth of the distance between Archaea and Bacteria. However, the distance between CP43 and CP47 (and also between D1 and D2 [19]) is of similar magnitude to that between Alpha and Beta, and to that between archaeal and bacterial RpoB, but substantially surpasses the distance between MSV and Cyanobacteria (Fig. 2). These observations suggest that ancestral proteins to CP43/CP47 and D1/D2 existed before the divergences of MSV.

We compared the within-group mean distances for Alpha, Beta, RpoB, and a concatenated dataset of ribosomal proteins compiled in a previous independent study [39] (see Supplementary Table S2). We consistently found that Vampirovibrionia and Margulisbacteria have larger within-group mean distances compared to Cyanobacteria, which suggests greater rates of evolution in the non-photosynthetic clades. These were larger for Margulisbacteria relative to the other two groups. Thus, RpoB in Vampirovibrionia and Margulisbacteria showed 1.6× and 4.0× larger corrected mean distances than Cyanobacteria, respectively (Supplementary Table S2). To put this into scale, RpoB from two strains of Margulisbacteria belonging to the genus *Termititenax* [8] or between two distant Gastranaerophilales (Vampirovibrionia) [41], show levels of divergence that surpass the largest distance between the two most distant extant Cyanobacteria. For example, the level of sequence identity for the two *Termititenax* is 78%, between two distant Gastranaerophilales is 70%, but for *Gloeobacter* spp. compared with any other cyanobacterium is about 84%. The latter likely being a much older diversification event. At the level of the concatenated ribosomal proteins dataset, Margulisbacteria showed an almost 2-fold larger within-group mean distance than Cyanobacteria. Faster rates of evolution are consistent with the trophic modes of the few characterized strains of Margulisbacteria and Vampirovibrionia, suggesting that these may be generalizable within their diversity, and resemble similar, but perhaps not as extreme, observations made for the Candidate Phyla Radiation (CPR) and DPANN Archaea [42,43]. In fact, the long branches of CPR and MSV artefactually attract each other in phylogenomic trees [39,44,45], potentially indicating convergent evolutionary processes.

#### 2.2.2 Deep-time duplications

**Fig. 3:**
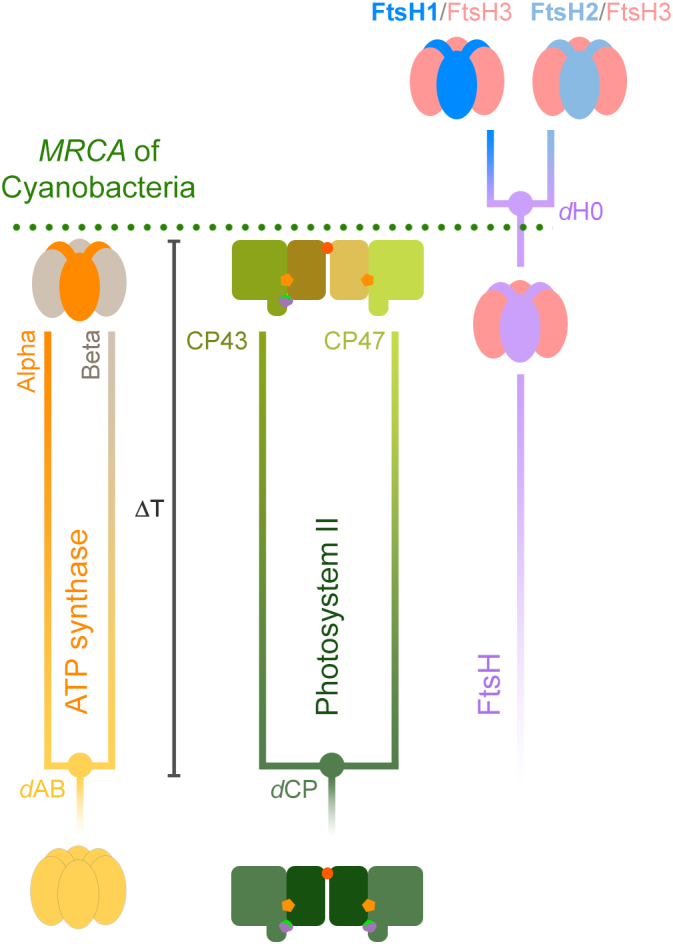
Schematic representation of ancestral duplication events. The MRCA of Cyanobacteria inherited a standard F-type ATP synthase, with a heterohexameric catalytic head (F_1_) made of alternating subunits Alpha and Beta; and a PSII with a heterodimeric core and antenna. ΔT denotes the span of time between the ancestral duplication events and the MRCA of Cyanobacteria. In the case of ATP synthase, this duplication is suspected to antedate the divergence of Bacteria and Archaea and their further diversification. FtsH contains an N-terminal membrane-spanning domain attached to a soluble domain consisting of an AAA (ATPase associated with diverse activities) module attached to a C-terminal protease domain. FtsH is universally conserved in Bacteria, has a hexameric structure like that of ATP synthase's catalytic head, and can be found usually as homohexamers, but also as heterohexamers. The MRCA of Cyanobacteria likely inherited three variant FtsH subunit forms, one of which appears to have duplicated after the divergence of the genus *Gloeobacter* and possibly other early-branching Cyanobacteria [47]. This late duplication led to FtsH1 and FtsH2, which form heterohexamers with FtsH3, following the nomenclature of Shao et al. [47]. FtsH1/FtsH3 is found in the cytoplasmic membrane of Cyanobacteria, while FtsH2/FtsH3 is involved in the degradation of PSII and other thylakoid membrane proteins.

We then compared the rates of evolution of CP43 and CP47 with those of Alpha and Beta cyanobacterial F-type ATP synthase, using a Bayesian relaxed molecular clock approach with identical calibrations, molecular clock parameters, and a simplified, but highly constrained sequence dataset (see Materials and Methods and Supplementary Text S2 for an expanded rationale and technical details). The goal of these experiments is not to use the clock to time the LUCA, the origin of photosynthesis or Cyanobacteria, but to measure the rates of protein evolution that are required to *model* or *simulate* any chosen span of time between the ancestral duplications and the MRCA of Cyanobacteria. We used an autocorrelated log normal model of rate variation with a non-parametric CAT+Γ model of amino acid substitutions to extract rates of evolution. We refer to the span of time between the duplication points leading to Alpha and Beta (***d*AB**), or to CP43 and CP47 (***d*CP**), and the MRCA of Cyanobacteria as ΔT (schematized in Fig. 3).

**Fig. 4:**
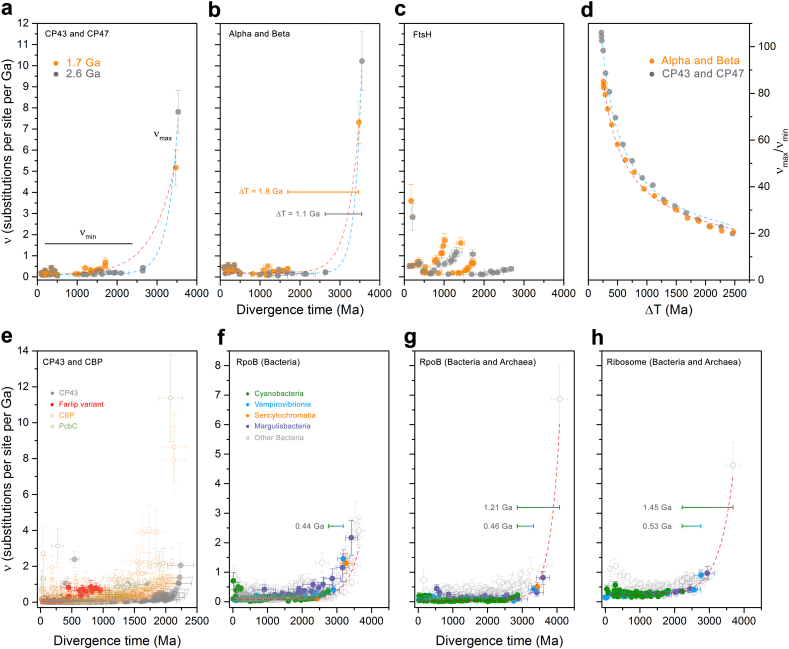
Comparison of the changes in the rates of evolution as a function of time. **a** Rates of CP43 and CP47 are simulated using two specific evolutionary scenarios (orange and grey traces). The orange trace was calculated under the assumption that the MRCA of Cyanobacteria occurred after the GOE, at ~1.7 Ga, while the duplication of CP43 and CP47 occurred at ~3.5 Ga. The fastest rate seen at the point of duplication is denoted as ν_max_, and stabilizes during the Proterozoic, ν_min_. The grey trace was calculated under the assumption that the MRCA of Cyanobacteria occurred before the GOE, at ~2.6 Ga. **b** Same calculations as **a** but performed on Alpha and Beta sequences of cyanobacterial and plastid ATP synthase. **c** Rates of evolution of cyanobacterial and plastid FtsH subunits assuming a MRCA of Cyanobacteria at ~1.7 and ~2.6 Ga. **d** Changes in the rates of evolution with varying ΔT for ATP synthase (orange) and PSII subunits (grey). **e** Changes in the rates of evolution as a function of time for a relaxed molecular clock computed with the full dataset of CP43 (full circles) and CBP sequences (open circles). **f** Changes in the rate of evolution of bacterial RpoB showing an exponential decrease in the rate of evolution. The bar denotes the span of time between the MRCA of the clade containing Vampirovibrionia and Cyanobacteria and the MRCA of Cyanobacteria (0.44 Ga). **g** Changes in the rate of evolution of bacterial and archaeal RpoB. The longer bar represents the span of time between the divergence of Archaea and Bacteria and the MRCA of Cyanobacteria (1.21 Ga). **h** Changes in the rate of evolution of a dataset of concatenated ribosomal proteins showing similar patterns of evolution as RpoB. Error bars on each data point are standard errors on the rate and mean divergence time.

In Fig. 4a–d we examine the changes in the rate of evolution under specific evolutionary scenarios. In the case of ATP synthase, we first assumed that the MRCA of Cyanobacteria occurred after the GOE to simulate scenarios similar to those presented in [11] or [46], at about 1.7 Ga; and that *d*AB occurred at 3.5 Ga (Δ**T** = **1.8 Ga**). Under these conditions the average rate of evolution of Alpha and Beta is calculated to be 0.28 ± 0.06 substitutions per site per Ga (δ Ga^−1^). We will refer to the average rate through the Proterozoic as *ν*_min_. In this scenario, the rate of evolution at the point of duplication, which we denote ν_max_, is 7.32 ± 1.00 δ Ga^−1^, making ν_max_/ν_min_ 26. In other words, when the span of time between the ancestral pre-LUCA duplication and the MRCA of Cyanobacteria is 1.8 Ga, the rate of evolution at the point of duplication is about 26 times greater than any rate observed through the diversification of Cyanobacteria or photosynthetic eukaryotes. To place these rates in the larger context of protein evolution, we encourage the reader to refer to Supplementary Text S1.

Now, if we consider a scenario in which *d*AB is 4.0 Ga and leaving all other constraints unchanged, ν_max_ is 6.02 ± 0.9 δ Ga^−1^ resulting in a ν_max_/ν_min_ of 21. If instead we keep the duplication at 3.5 Ga but assume that the MRCA of Cyanobacteria occurred before the GOE to simulate a more conservative scenario, at 2.6 Ga (Δ**T** = **1.1 Ga**), we obtain that ν_min_ is consequently slower, 0.25 ± 0.06 δ Ga^−1^, when compared to a post-GOE ancestor. This older MRCA (smaller ΔT) thus leads to a rise in ν_max_, calculated to be 10.22 ± 1.37 δ Ga^−1^ and leading to a ν_max_/ν_min_ of 40. Given that the phylogenetic distance is a constant, the rate of evolution increases with a decrease in ΔT following a power law function. We had observed nearly identical evolutionary patterns for the core RC proteins D1 and D2 of PSII [19]. The change in ν_max_/ν_min_ as a function of ΔT is shown in Fig. 4d.

The core antennae of PSII, CP43 and CP47, showed patterns of divergence similar to those of Alpha and Beta, both in terms of ν_max_ at the point of duplication (*d*CP) and ν_min_ (Fig. 4a and b, and Table 1). However, CP43 and CP47 featured slightly slower rates of evolution than Alpha and Beta, which is consistent with the fact that CP43 and CP47 show less sequence divergence between orthologues at all taxonomic ranks of oxygenic phototrophs (see Supplementary Table S3).

**Table 1:** Rates of evolution.

	ν_min_ (δ Ga^−1^)		ν_max_ (δGa^−1^)		ν_max_/ν_min_	
	MRCA of Cyanobacteria/Duplication point					
	1.7/3.5 Ga	2.6/3.5 Ga	1.7/3.5 Ga	2.6/3.5 Ga	1.7/3.5 Ga	2.6/3.5 Ga
Alpha/beta	0.28 ± 0.06	0.25 ± 0.06	7.32 ± 1.00	10.22 ± 1.37	26	40
CP43/CP47	0.19 ± 0.05	0.16 ± 0.04	5.17 ± 0.84	7.81 ± 1.01	27	49
FtsH1	1.32 ± 0.29	1.00 ± 0.23	0.66 ± 0.21	0.32 ± 0.11	0.5	0.32
FtsH2	0.24 ± 0.06	0.16 ± 0.04			2.75	2

We then studied a relatively recent gene duplication event (Fig. 4c), which occurred long after the LUCA, but also after the MRCA of Cyanobacteria: that leading to Cyanobacteria-specific FtsH1 and FtsH2 [47]. This more recent duplication served as a point of comparison and control (see Fig. 3 for a scheme). In marked contrast to *d*AB, the rate at the point of duplication was 0.66 ± 0.21 δ Ga^−1^. We also found that FtsH1 is evolving nearly 6 times faster than FtsH2 under the assumption that MRCA of Cyanobacteria occurred at 1.7 Ga (Table 1). If the MRCA of Cyanobacteria is assumed to have occurred at 2.6 Ga, all rates slowdown proportionally. This late duplication is consistent with classical neofunctionalization, in which the gene copy that gains new function experiences an acceleration of the rate of evolution [48,49]. Like PSII and ATP synthase, the calculated rates of evolution match observed distances as estimated by the change in the level of sequence identity as a function of time, in which the fastest evolving FtsH1 accumulated greater sequence change than FtsH2 in the same period (Supplementary Table S3).

Given that the complex evolution of CP43 and CBP involved several major duplication events and potentially large variations in the rate of evolution (Fig. 1 and Supplementary Fig. S1), we carried out a molecular clock analysis of a large dataset of 392 CP43 and 465 CBP proteins using cross-calibrations across paralogues as described in Supplementary Text S2. Clocks were executed with no constraint on the MRCA of Cyanobacteria. The estimated mean divergence time for the oldest node, the duplication at the origin of CBP, is 2.23 Ga (95% confidence interval, CI: 1.90–2.69 Ga) using an autocorrelated log normal model (see Fig. 4e, Supplementary Fig. S5 for a chronogram, and Supplementary Table S4 for a comparison of estimated ages under different models). The mean divergence time for the node representing the CP43 inherited by the MRCA of Cyanobacteria was 2.22 Ga (95% CI: 1.88–2.68 Ga). Thus, a span of time of only 15 Ma is measured between these two mean ages. The average rate of evolution of CP43, not including CBP sequences, was found to be 0.14 ± 0.05 δ Ga^−1^, which is in the same range as determined in the simplified, but highly constrained experiment above. We noted a 6-fold increase in the rate of evolution associated with the duplication leading to the FaRLiP-CP43 variant (Fig. 4e). This duplication led to an acceleration of the rate similar in magnitude to that of FtsH1/FtsH2 and is consistent with neofunctionalization as the photosystems evolved to use far-red light and bind chlorophyll *f*.

CBP sequences, on average, display rates of evolution about three times faster than CP43 (Fig. 4e). However, the serial duplications that led to the evolution of CP43-derived light harvesting complexes resulted in accelerations in the rate of evolution of a similar magnitude as observed for *d*AB and *d*CP. The largest of these is associated with the origin of PcbC [31], a variant commonly found in heterocystous Cyanobacteria and Cyanobacteria that use alternative pigments, such as chlorophyll *b*, *d* and *f*, but that remains largely uncharacterized. The ancestral node of PcbC was timed at 2.07 Ga (95% CI: 1.76–2.50 Ga) with a rate of 11.7 ± 2.42 δ Ga^−1^, decelerating quickly, but stabilizing at about four times faster rates than the average rate of CP43. We find it noteworthy that the fast rates of evolution at the origin of CBP are not associated with very large spans of time between these and CP43, nor did it result in very old root node ages despite the use of very broad constraints.

### 2.3 Species divergence

**Fig. 5:**
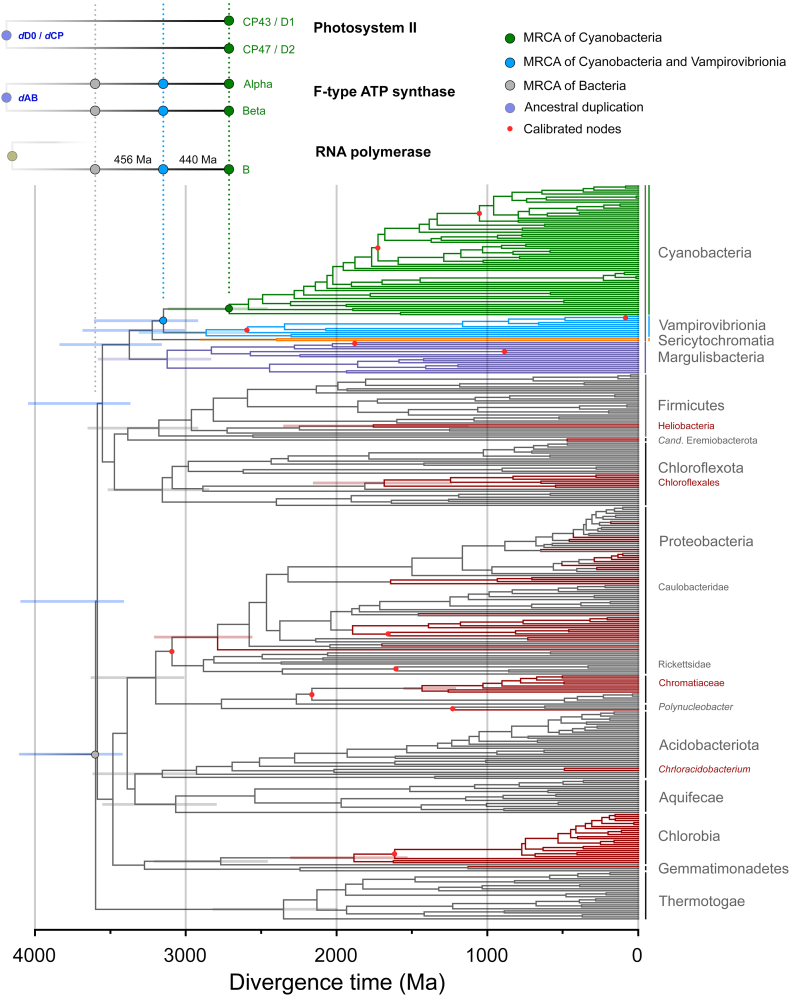
Bayesian relaxed molecular clock of bacterial RpoB. The tree highlights the span of time between the MRCA of Cyanobacteria (green dot) and their closest relatives. Calibrated nodes are marked with red dots. Anoxygenic phototrophs are highlighted in red branches and non-phototrophic bacteria in grey, with the exception of Margulisbacteria, Sericytochromatia and Vampirovibronia, which are coloured as indicated in the figure. Superimposed at the top are the implied distribution and divergence time for ATP synthase and PSII. Horizontal bars within the tree mark 95% confidence intervals. These are shown in selected nodes of interest for clarity but see also Table 2.

To understand the evolution of MSV relative to Cyanobacteria we wished to apply a molecular clock to a system where the calculated rates could be compared to observed rates, as determined by distances between species of known divergence times or at similar taxonomic ranks. We found RpoB to be suitable for this because it has been inherited vertically with few instances of horizontal gene transfer and had enough signal to resolve known phylogenetic relationships between and within clades. We implemented a set of 12 calibrations across bacteria, including two calibrations on Margulisbacteria and two in Vampirovibrionia with the aim of covering both slower and faster evolving lineages. The following results are based on an autocorrelated log normal molecular clock using CAT+Γ, a root constrained with a broad interval ranging from between 4.52 and 3.41 Ga, and as described in Materials and Methods and Supplementary Text S3 (Fig. 5). We found this to perform well and provided results comparable to other independent studies that did not combine a full set of MSV sequences and other clades with phototrophs in a single tree (Table 2). Nonetheless, a pipeline of sensitivity experiments tested the dependency of these results on models and prior assumptions: these are shown and described in Supplementary Figs. S6 and S7.

**Table 2:** Divergence time estimates.

Event	RpoB (Ga) (95% CI)	Concatenated ribosomal proteins	TimeTree compilation^a^	Shih et al.^b^	Magnabosco et al.^c^
Divergence of Thermotogae	3.64 (3.43–4.11)	3.01 (2.64–3. 51)	3.81 (3.51–4.16)		
Divergence of Margulisbacteria	3.42 (3.17–3.86)	2.93 (2.58–3.44)			
Divergence of Sericytochromatia	3.26 (3.01–3.68)				
Divergence of Vampirovibrionia/Stem Cyanobacteria	3.19 (2.92–3.61)	2.76 (2.40–3.23)	3.19 (2.26–3.55)	2.54 (2.09–3.06)	2.77 (2.39–2.93)
MRCA of Cyanobacteria	2.75 (2.46–3.13)	2.22 (1.86–2.64)	2.24 (1.81–2.82)	2.02 (1.72–2.37)	2.24 (1.91–2.41)
Divergence of Heliobacteria	2.28 (1.74–2.78)		2.23 (1.99–2.48)		
MRCA of Heliobacteria	1.78 (1.13–2.36)				
Divergence of phototrophic Chloroflexota	1.83 (1.41–2.25)		1076	1.098 (0.80–1.41)	2.86 (2.49–3.00)
MRCA of phototrophic Chloroflexota	1.71 (1.24–2.16)			0.86 (0.61–1.14)	1.98 (1.68–2.43)
Divergence of phototrophic Chlorobia	2.80 (2.45–3.20)				2.56 (2.26–2.85)
MRCA of phototrophic Chlorobia	1.91 (1.53–2.31)		525		1.71 (1.64–1.95)
Earliest phototrophic Proteobacteria	2.82 (2.55–3.21)		2.48 (2.04–2.62)		
Divergence of *Chloracidobacterium*	2.04 (1.53–2.51)				

a Data from TimeTree compiling estimated divergence times from independent studies as described in ref. [140]. For the divergence of Thermotogae the estimated age was taken from eight different studies (*n* = 8). For the divergence of Melainabacteria (or divergence from Cyanobacteria's closest relatives), *n* = 10. For stem Heliobacteria, *n* = 2. For stem phototrophic Chloroflexota and Chlorobia, the values were reported from a single study, Marin et al. [65]. For earliest potential phototrophic Proteobacteria, *n* = 3. This latter was taken as the node made by the clades including *Niveispirillum lacus* and *Rhodobacter sphaeroides* to match our RpoB tree. The independent studies used by TimeTree to generate the estimated ages are listed in Supplementary Table S8.

b Data taken from ref. [11] Model T68 for the cyanobacterial dates. That is, no GOE calibration with a calibration on *Bangiomorpha*. The dates on Chloroflexota were taken from ref. [141].

c Data taken from ref. [12] Model A. This used a 1.2 Ga calibration on heterocystous Cyanobacteria.

The root of the tree (divergence of Thermotogae) was timed at 3.64 Ga (95% CI: 3.42–4.11 Ga) and the divergence of Cyanobacteria at 2.74 Ga (95% CI: 2.46–3.12 Ga). Thus, the span of time between the mean age of the root of the bacterial tree and the mean age of the MRCA of Cyanobacteria was calculated to be 0.89 Ga and that between Vampirovibrionia and the latter was found to be 0.44 Ga (Figs. 4f and 5). This result is consistent with previous studies using different rationales, datasets and calibrations [11,12].

We also noted an exponential decrease in the rates of evolution of RpoB through the Archean, which stabilized at current levels in the Proterozoic (Fig. 4f). The rate at the root node was calculated to be 2.37 ± 0.45 δ Ga^−1^ and the average rate of evolution of RpoB during the Proterozoic was found to be 0.19 ± 0.06 δ Ga^−1^. The average rate of cyanobacterial RpoB was 0.14 ± 0.04 δ Ga^−1^; for Margulisbacteria was 0.44 ± 0.17 δ Ga^−1^, and for Vampirovibrionia 0.19 ± 0.05 δ Ga^−1^: about 3.1× and 1.3× the mean cyanobacterial rate, respectively. These rates agree reasonably well with the observed distances (Supplementary Table S2), further indicating that the calibrations used in these clades performed well. Nevertheless, we suspect that the values for MSV represent underestimations of the true rates of evolution (slower than they should be), as some of the clades that include symbionts still appear much older than anticipated from their hosts. This is probably due to the relaxed nature of molecular clocks, which tends to smooth rates out [50]. For example, Gastranaerophilales were timed to have originated long before the emergence of animals (95% CI: 1.91–2.74 Ga). This dichotomy in the rates was also detected for Rickettsidae (0.40 ± 0.11 δ Ga^−1^) made up of symbionts and parasites, when compared to Caulobacteridae (0.17 ± 0.05 δ Ga^−1^) of the Alphaproteobacteria, which included free-living and phototrophic strains. We also noted the same potential underestimation for many strains of Rickettsidae, which appeared to be much older than their symbiotic associations would suggest, despite the provided constraints.

A more complex, but commonly used model like CAT+GTR+Γ implementing a birth-death prior with ‘soft bounds’ on the calibrations, resulted in rates that were smoothed out, and which translated into spread-out divergence times with Margulisbacteria and Vampirovibrionia evolving at 1.9× and 0.7× times the cyanobacterial rates, respectively (Supplementary Fig. S7, model **n** to **p**). These rate effects are thus translated into a very late Mesoproterozoic age for Cyanobacteria, and a relatively older divergence time for Vampirovibrionia: results that replicate those presented in ref. [46].

To investigate the effect of the age of the root on the divergence time of MSV and Cyanobacteria we also varied the root prior from 3.2 to 4.4 Ga (Supplementary Fig. S6). We noted that regardless of the time of origin of Bacteria (approximated by the divergence of Thermotogae in our analysis), a substantially faster rate is required during the earliest diversification events, decreasing through the Archean and stabilizing in the Proterozoic. This matches well the patterns of evolution of ATP synthase and PSII core subunits as shown in the previous section.

**Fig. 6:**
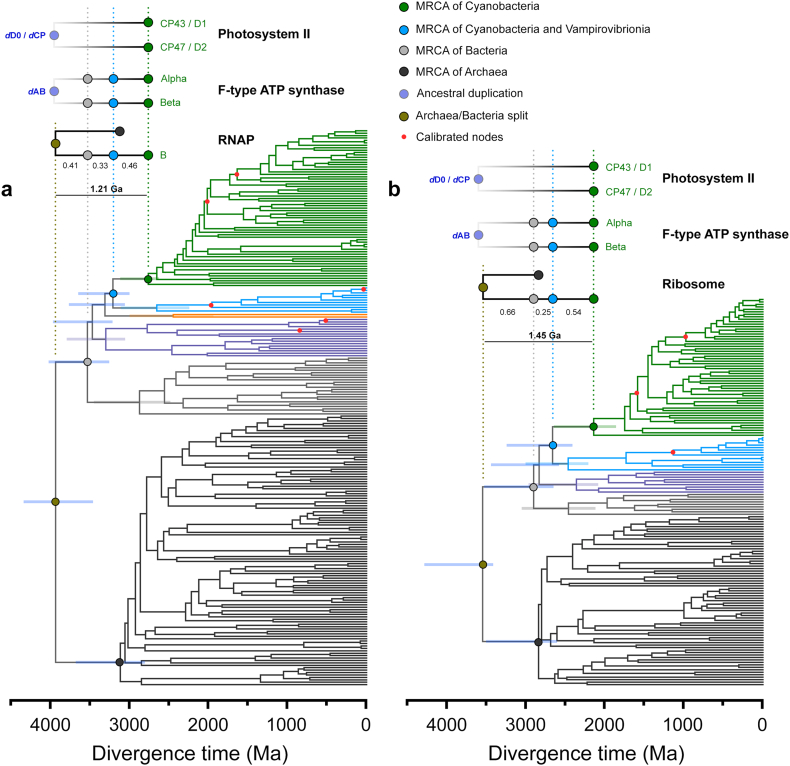
Comparison of relaxed molecular clocks of RpoB (**a**) and a concatenated dataset of ribosomal proteins (**b**) that include sequences from a diverse set of Archaea. RNAP stands for RNA polymerase. Clocks were calculated using a calibration on the root with a maximum of 4.52 Ga and a minimum of 3.41 Ga and applying a log normal autocorrelated clock with CAT+Γ model. Bars on selected nodes denote 95% confidence intervals.

We then compared the above RpoB molecular clock result with a different clock that included a set of 112 diverse sequences from Archaea, in addition to the sequences from Thermotogae, MSV, and Cyanobacteria, but removing all other bacterial phyla (Fig. 6a). We found that the calculated average rate of evolution of bacterial RpoB during the Proterozoic was slower (0.09 ± 0.03 δ Ga^−1^) than in the absence of archaeal sequences (0.19 ± 0.06 δ Ga^−1^), resulting in overall older mean ages (see Fig. 4g). However, the rate at the Bacteria/Archaea divergence point was 6.87 ± 1.17 δ Ga^−1^, similar to the rate for *d*AB and *d*CP, requiring therefore an exponential decrease in the rates similar to that observed for ATP synthase and the PSII core subunits. Notably, this rate and exponential decrease is associated with a large span of time between the mean estimated ages of the LUCA and the MRCA of Cyanobacteria, calculated in this tree to be 1.21 Ga. The span between Vampirovibrionia and Cyanobacteria was found to be 0.46 Ga (Fig. 6a).

Fig. 5 also highlights that the none of the most recent common ancestors of the groups containing anoxygenic phototrophs nor their divergence from their closest non-phototrophic relatives appears to be older than one of the oldest and best accepted geochemical pieces of evidence for photosynthesis at 3.41 Ga [51] (Table 2). For example, the MRCA of Heliobacteria or of phototrophic Chlorobia, containing homodimeric type I RCs, are likely to have existed only after the GOE, even allowing for large uncertainties in the calculations. These clades have traditionally been described as harbouring primitive forms of photosynthesis.

Finally, we performed a similar molecular clock analysis on a subset of concatenated ribosomal proteins published by Hug et al. [39] that included Archaea, Thermotogae, Margulisbacteria, Vampirovibrionia and Cyanobacteria. Even though this dataset generated younger ages compared to RpoB (see Table 2 and Fig. 6), a similar exponential decrease in the rates was observed with a rate at the Bacteria/Archaea divergence point measured at 4.62 ± 0.71 δ Ga^−1^ and an average rate of bacterial ribosomal protein evolution of 0.30 ± 0.07 δ Ga^−1^ (Fig. 4h). These rates were associated with a span of time between the mean estimated ages of the LUCA and the MRCA of Cyanobacteria of 1.45 Ga; and between Vampirovibrionia and Cyanobacteria 0.54 Ga (Fig. 6b).

### 2.4 Structural analysis

A fundamental premise of our investigation is that water oxidation started before the duplication of D1 and D2, and CP43 and CP47. The rationale behind this premise has been laid out before [18,52], and more extensively recently [19]. If this rationale is correct, it raises the question of how the CP47/D2 side of the RC lost its capacity to carry out water-splitting catalysis.

#### 2.4.1 Ancestral sequence reconstruction

To gain further insight on the nature of the structural site around the water-oxidizing complex in the ancestral photosystem, we used ancestral sequence reconstruction to predict the most probable ancestral states. We will refer to the ancestral protein to D1 and D2 as D0 (Supplementary Fig. S8). We generated 14 predicted D0 sequences using a combination of three ASR methods and amino acid substitution models. On average the 14 D0 sequences had 87.12 ± 0.55% sequence identity indicating that the different algorithms provided largely consistent results. While the regions that include all transmembrane helices are aligned unambiguously, the N-terminal and C-terminal ends were aligned less confidently due to greater sequence variability at both ends. Nonetheless, we found that the predicted D0 sequences retain more identity with D1 than D2 along the entire sequence. The average level of sequence identity of D0 sequences compared with the standard D1 (PsbA1) of *Thermosynechococcus vulcanus* was found to be 69.58 ± 0.55%, and for D2 it was 36.32 ± 0.15% from the same organism.

The ligands to the Mn_4_CaO_5_ in PSII are provided from three different structural domains (Supplementary Fig. S9): 1) the D1 ligands D170 and E189, located near the redox Y_Z_. 2) The D1 ligands H332, E333, D342, and A344 located at the C-terminus. 3) The CP43 ligands, E354 and R357, located in the extrinsic loop between the 5th and 6th helices, with the latter residue less than 4 Å from the Ca atom. Remarkably, there is structural and sequence evidence supporting the loss of ligands in these three different regions of CP47/D2.

In all the D0 sequences, at position D1-170 and 189, located in the unambiguously aligned region, the calculated most likely ancestral states were E170 and E189, respectively. The mutation D170E results in a PSII phenotype with activity similar to that of the wild-type [53]. At position D1-170, a glutamate was predicted with average posterior probabilities (PPs) of 44.2% (FastML), 67.2% (MEGA) and 77.0% (PAML). At position D1-E189, the average PPs for glutamate were 31.1% (FastML), 35.2% (MEGA) and 40.2% (PAML). The distribution of PPs across a selection of D0 sequences and key sites is presented in Supplementary Fig. S10 and Table S5. In contrast, D2 has strictly conserved phenylalanine residues at these positions, but the PP of phenylalanine being found at either of these positions was less than about 5% for all predicted D0 sequences. As a comparison, the redox active tyrosine residues Y_Z_ (D1-Y161) and Y_D_ (D2-Y160), which are strictly conserved between D1 and D2, have a predicted average PPs of 68.8% (FastML), 98.8% (MEGA) and 98.6% (PAML). Therefore, the ligands to the catalytic site in the ancestral protein leading to D2 were likely lost by direct substitutions to phenylalanine residues, while retaining the redox active D2-Y160 (Y_D_) and H189 pair (Supplementary Fig. S9).

#### 2.4.2 A structural rearrangement

**Fig. 7:**
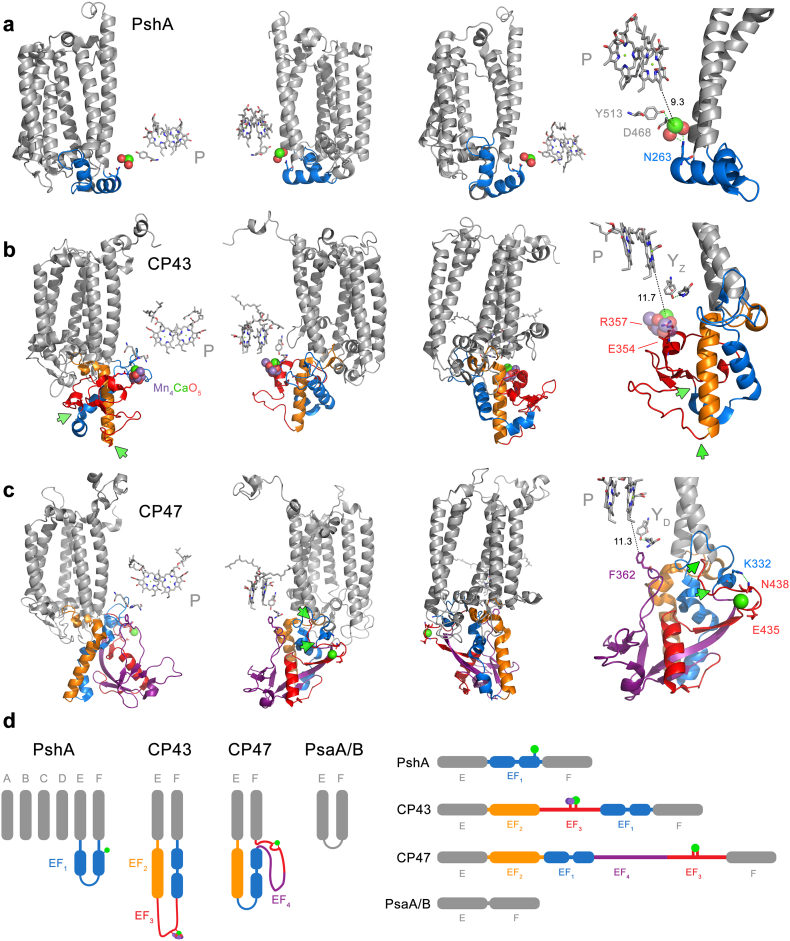
Structural rearrangements of the large extrinsic loop of CP43 and CP47. **a** The antenna domain of heliobacterial PshA is shown in three different rotated perspectives. Only the first six transmembrane helices of the antenna are shown for clarity. A Ca at the electron donor side is bound from an extrinsic loop between the 5th (E) and 6th (F) helices. This extrinsic loop, EF_1_ (blue), is made of two small alpha helices. The fourth molecular view furthest to the right shows the link between the electron donor site and EF_1_ in closer detail. **b** The CP43 subunit of PSII with the extrinsic loop shown in colours. **c** The CP47 subunit of PSII. Immediately after the 5th helix (E), a long alpha helix protrudes outside the membrane in both CP43 and CP47 and showing structural and sequence identity (orange). We denote this helix EF_2_. After EF_2_ structural differences are noticed between CP43 and CP47 as schematized in panel **d**. In CP43, after helix EF_2_ a loop is found (shown in red ribbons), which we denote EF_3_. This contains the residues that bind the Mn_4_CaO_5_ cluster and it is followed by a domain that resembles EF_1_ in the HbRC at a structural level. In CP47, EF_3_ and EF_1_ retain sequence identity with the respective regions in CP43. CP47 has additional sequence that is not found in CP43 (EF_4_, purple). The green arrows mark the position at which the domain swap occurred in CP43 relative to CP47. We found that the CP43-E354 and R357 are found in the equivalent domain in CP47 as E436 and N438 coordinating a Ca atom. N438 (EF_3_) links to EF_1_ via K332. It is unclear if the EF_1_ region in the HbRC is strictly homologous to that in CP43 and CP47 as very little sequence identity is found between the two: however, a couple of conserved residues between all EF_1_ may suggest it emerged from structural domains present in the ancestral RC protein (see Supplementary Fig. S12).

Prompted by the finding of a Ca-binding site at the electron donor site of the homodimeric type I RC of Heliobacteria (Firmicutes) with several similarities to the modern Mn_4_CaO_5_ cluster of PSII, including a link to the antenna domain and the C-terminus [20], we revisited the sequences and structural overlaps of CP43 and CP47. We found that a previously unnoticed structural rearrangement within the extrinsic loop occurred in one subunit relative to the other (marked EF_3_ and EF_4_ in Fig. 7, Supplementary Figs. S11 and S13). CP43 retains the simplest domain, being about 60 residues shorter than CP47. If CP43 retains the ancestral fold, the additional sequence in CP47's swapped domain (EF_4_ in Fig. 7d) would have contributed to the loss a catalytic cluster as it inserted one phenylalanine residue (CP47-F360) into the electron donor site, less than 4 Å from Y_D_. An equivalent residue does not exist in CP43.

We then noted that in the swapped region (EF_3_ in Fig. 7d), sequence identity is retained between CP43 and CP47 (Supplementary Fig. S11). We found that CP43-E354 and R357 are equivalent to CP47-E435 and N438 in the *T. vulcanus* structure. An inspection of this structure showed that these two residues specifically bind a Ca atom of unknown function (Fig. 7c and d). The presence of an equivalent glutamate to CP43-E354 in CP47 is consistent with this being already present before duplication and therefore, at an early stage of photosystem evolution.

#### 2.4.3 Gene overlap

Finally, a peculiar but well-known trait conserved across Cyanobacteria and photosynthetic eukaryotes is that the 5′ end of the *psbC* gene (CP43) overlaps with the 3′ end of the *psbD* gene (D2) usually over 16 bp (Supplementary Table S6). The *psbD* gene contains a well-defined Shine-Dalgarno ribosomal binding site downstream of the *psbC* gene and over the coded D2 C-terminal sequence [[54], [55], [56]]. The evolution of this unique gene overlap has no current explanation in the literature, but its origin could have disrupted the C-terminal ligands of the ancestral protein leading to the modern D2, an event that would have contributed to heterodimerization.

## 3 Discussion

### 3.1 Origin of oxygenic photosynthesis and rates of evolution

This work was not explicitly aimed at providing an exact time of origin for the LUCA, the MRCA of Cyanobacteria, or the origin of photosynthesis. The use of different datasets, calibration strategies, chosen clock parameters, and researchers' preferences can result in widely varying calculated rates, which can translate into disparate estimated ages, see for example [46,57]. Instead, rather than arguing in favour or against a particular age or methodology, our objective was to contrast the evolution of oxygenic photosynthesis with the evolution of enzymes whose origin is less controversial. The duplication of ATP synthase's ancestral catalytic subunit, and the archaeal/bacterial divergence of RNA polymerase and the ribosome, are some of the oldest evolutionary events known in biology [[21], [22], [23], [24], [25], [26], [27], [28], [29]]. When taken on their own, their phylogenies are often interpreted as evidence of the earliest origin. We have shown here and in our previous work [19] that PSII has patterns of molecular evolution that closely parallel those of these most ancient systems. These patterns do not emerge from the application of any particular molecular clock approach or model of rate change, but from the inherently long phylogenetic distance that separates Alpha and Beta, archaeal and bacterial RNA polymerase and ribosomes, or the core subunits of PSII, in combination with the slow rates of evolution that these enzymes have featured through their multibillion-year diversification process. These results have profound implications because it cannot now be taken for granted that there was ever a long period of time between the origin of life and the origin of anoxygenic photosynthesis, followed by another long period of time between the origin of anoxygenic photosynthesis and the origin of oxygenic photosynthesis.

The exponential decrease in the rates of evolution observed in the studied systems, even when the span of time between the ancestral duplications (or the LUCA) and the MRCA of Cyanobacteria was considered to be well over a billion years, is consistent with our assessment that a large ΔT for the evolution of the core subunits of ATP synthase and PSII cannot be explained entirely by duplication-driven effects. Instead, we speculate that these effects are the result of the faster rates of evolution that are expected to have occurred during the earliest history of life [[58], [59], [60], [61], [62], [63]]. It is worth highlighting that ATP synthase, RNA polymerase, the ribosome, and the photosystems, are all complex molecular machines, with crucial functions and under strict regulation. These features largely explain their slow rates of evolution and the high degree of sequence (structural and functional) conservation through geological time. A similar level of complexity, however, can be traced back to the last common ancestor of each one of these molecular systems regardless of how ancient they truly are. Now, given that the rates of evolution of the core of PSII have remained slower than those of ATP synthase for billions of years, it could imply that the duplications of the core of PSII occurred before the duplication leading to Alpha and Beta. In consequence, to argue that oxygenic photosynthesis is a late evolutionary innovation relative to the origin of life, one must first demonstrate that the rates of protein evolution of PSII and ATP synthase catalytic core subunits, RNA polymerase, and the ribosome, have not remained proportional to each other during the Archean. That is to suggest that PSII—exclusively—experienced unprecedentedly faster rates of evolution during the heterodimerization process, and then, the rates stopped abruptly with the MRCA of Cyanobacteria. One could potentially argue that the origin of water oxidation itself could account for unprecedentedly faster rates. However, a decrease in ΔT of about 60% (from 1.1 to 0.46 Ga, for example) would result in a 30-fold additional increase in the rates at the earliest stages of diversification, resulting in photosystems that would be evolving at rates that surpass those of the fastest evolving peptide toxins (Fig. 4d and Supplementary Text S1). Furthermore, it should be noted that a ΔT of over a billion years already includes a period of extremely rapid evolution at the origin of all these ancient systems.

### 3.2 Diversification of bacteria

It has been postulated before that the diversification of the major phyla of Bacteria occurred very rapidly, starting at about 3.4 Ga and peaking at about 3.2 Ga, in what has been dubbed the Archean Genetic Expansion [64], but see also [46,65]. A recent study suggested that the major groups of Bacteria and Archaea diversified rapidly after the LUCA and hypothesized that the long distance between archaeal and bacterial ribosomal proteins could be attributed to fast rates of ribosome evolution during their divergence [66], but see also [67,68]. Our analyses suggest that the more explosive the diversification of prokaryotes, the greater the chance that photosynthetic water-splitting is an ancestral trait of life. Yet, if phototrophic communities—whether oxygenic or not—already existed 3.2 to 3.4 Ga ago, as it is supported by the geochemical record [51,69], then the earliest bacteria were likely photosynthetic. This is consistent with the evolution of RC proteins, which suggests that the structural and functional specialization that led to the two photosystem types, antedated the diversification processes leading to the known phyla containing photosynthetic bacteria [35]. It also explains why none of the groups of extant photosynthetic bacteria appear to be older than the earliest geochemical evidence for photosynthesis or hardly older than the GOE. The presented data is also consistent with recent studies of the oxygenation of the planet, which suggests that even if photosynthetic O_2_ evolution started as early as the oldest rocks, the properties of early Earth biogeochemistry would have maintained very low concentrations of O_2_ over the Archean [[70], [71], [72]].

### 3.3 Photosystem structural constraints

**Fig. 8:**
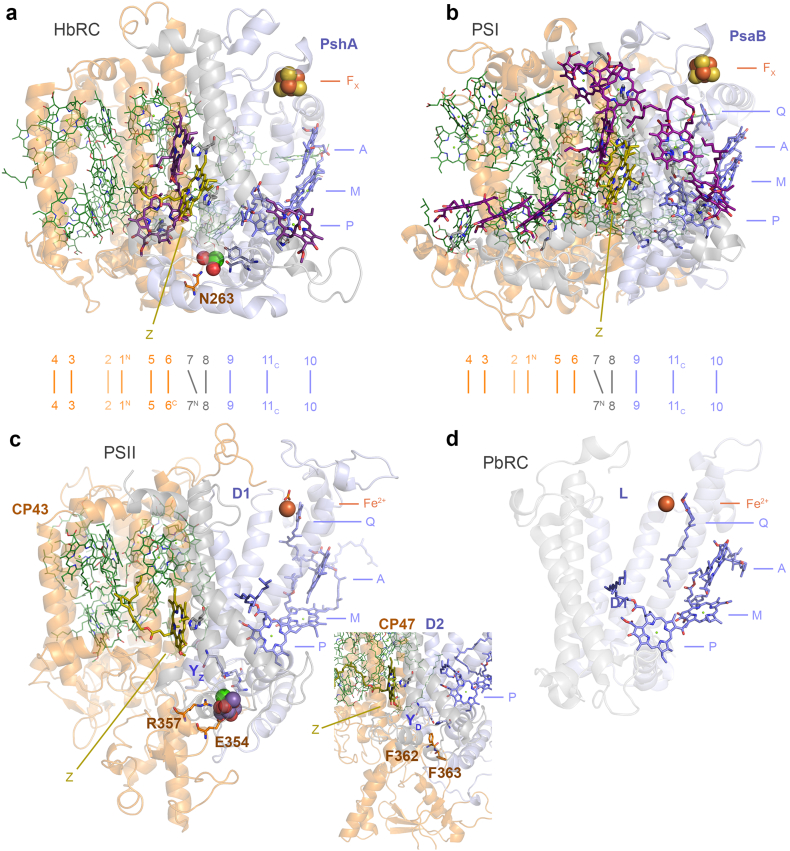
Structural comparisons of the antenna and core domains of photosynthetic RCs. **a** A monomeric unit (PshA) of the homodimeric RC of Heliobacteria (HbRC, Firmicutes). The RC protein is made up of 11 transmembrane helices. The first six N-terminal helices are traditionally considered as the antenna domain (orange ribbons), while the last five helices are the RC core domain (grey and light-blue ribbons). Below the structure, the organization of the 11 helices is laid down linearly for guidance: 1^N^ denotes the first N-terminal helix and 11_C_ the last C-terminal helix. P denotes the “special pair” pigment; M, the “monomeric” bacteriochlorophyll electron donor; and A, the primary electron acceptor. F_X_ is the Fe_4_S_4_ cluster characteristic of type I RCs. Antenna pigments bound by the antenna domain are shown in green lines. Antenna pigments bound by the 7th and 8th helices are shown as purple sticks, except for the bacteriochlorophyll molecule denoted as Z, in olive green and bound by the 8th helix. The antenna domain connects the electron donor side of the RC via N263 and a Ca-binding site. **b** A monomeric unit of PSI (PsaB, Cyanobacteria) following a similar structural organization and nomenclature as in panel **a**. Unlike the HbRC, PSI binds quinones (Q) as intermediary electron acceptors between A and F_X_. **c** A monomeric unit of PSII (CP43/D1 and CP47/D2 in the inset). It has a structural organization similar to that of type I RCs. However, the monomeric unit is split into two proteins after the 6th transmembrane helix. The Mn_4_CaO_5_ cluster is coordinated by D1 but is directly connected to the antenna domain via E354 and R357 in manner that resembles the Ca-binding site found at the electron donor site of the HbRC. **d** A monomeric unit of an anoxygenic type II reaction (PbRC, Proteobacteria). Unlike type I RCs, type II RCs lack F_X_. Instead, a non-heme Fe^2+^ is found linking the RC proteins. The PbRC lacks antenna domain and the antenna pigment Z bound at the equivalent 8th helix.

We have shown that the phylogenetic distance between CP43/CP47 and other type I RC proteins is the largest distance after that between type I and type II RC proteins (Supplementary Fig. S2). The antenna domain extends to the 8th helix, both in type I RCs and in PSII, with the latter retaining one antenna chlorophyll in the equivalent 8th helix (marked Z in Fig. 8) and substantial sequence identity around this chlorophyll's binding site, as shown before [35,73]. These conserved features indicate that at no time during the evolution of PSII was it devoid of core antenna proteins. In other words, CP43/CP47 are a descendant of the ancestral core antenna of type II photosystems. Both structural and phylogenetic evidence is in agreement and indicates that the ancestral type II RC, at the dawn of photosynthesis, was architecturally like water-splitting PSII.

Sequence reconstruction of the ancestral subunit to D1 and D2 is consistent with the existence of a highly oxidizing homodimeric photosystem, though transient and short-lived [19], that could split water in either side of the RC [52]. The structural comparisons between CP43/D1 and CP47/D2 suggest a mechanism for heterodimerization and loss of catalysis on one side that accounts for all ligands. These include direct amino acid substitutions, the loop swap within the extrinsic region of the ancestral protein to CP47, and the gene overlap between *psbC* (CP43) and *psbD* (D2). It would be difficult to reconcile these unique structural and genetic features with a scenario in which PSII evolved water oxidation at a late stage, or starting as a purple anoxygenic type II RC, once the heterodimerization process was well underway or completed, or in the absence of core antenna domains, given that these interact directly with the electron donor side of PSII, in a manner strikingly similar to that of the Ca-site in the homodimeric type I RC of the Firmicutes [20]. A conserved Ca-binding site has also been recently confirmed for the RC of the Chlorobia [74], as predicted by Cardona and Rutherford [20]. What is more, these structural and functional features, together with the ASR analysis (Supplementary Fig. S8 and S9) suggest that the atypical forms of D1, lacking ligands to the Mn_4_CaO_5_ cluster, such as the chlorophyll *f* synthase [33,75], or the “rogue” or “sentinel” D1 [32,76], diverged from forms of D1 that were able to support water oxidation, but that could antedate the MRCA of Cyanobacteria [2].

We have calculated that (oxygenic) PSII has experienced the slowest rates of evolution of all photosystems, with the core of the anoxygenic type II RC evolving on average about five times faster than the core of PSII [19]. That faster rate has led to conspicuous structural changes of the anoxygenic RC relative to PSII and type I photosystems. The outcome of these differences in the rates are not only visually apparent (Fig. 8), but also the anoxygenic type II RC among all photosystems has experienced the greatest erosion of sequence and structural symmetry and even complete loss of structural domains [20,77]. These differences in the rates and associated structural differences appear inconsistent with a scenario in which PSII experienced unprecedentedly fast rates of evolution. Counterintuitively, because PSII is the slowest evolving of all photosystems and among the slowest evolving enzymes known; and because that has been the case for a couple of billions of years, if not much longer, it follows then that PSII is the most likely RC to have changed the least since the origin of photosynthesis.

### 3.4 Origin of homodimeric water-splitting photosystems

Compelling scenarios that discuss the origin of water oxidation propose Mn-oxidizing photosynthesis as a key transitional stage [18,[78], [79], [80], [81], [82]]. Few of these explicitly state whether this transition should have occurred before or after the duplications that led to CP43/D1 and CP47/D2. Cardona et al. [2] correlated the phylogenetic branching pattern of the D1 tree to transitional stages from a Mn^II^-oxidizing photosystem towards the emergence of the full Mn_4_CaO_5_ cluster. This correlation was later revisited based on the extended analysis presented in ref. [19], in line with previous suggestions [52], and it is indeed not now supported by ASR analysis as further indicated in this work.

In another scenario, Fischer et al. [78] hypothesized that a Mn^II^-oxidizing photosystem occurred at the homodimeric stage and considered that the D1 and D2 duplication enabled water oxidation. Fischer et al. [78] also speculated using geochemical evidence from Mn deposits in the ~2.41 Ga Koegas Subgroup of the Kaapvaal Craton of South Africa [83], that the duplication occurred just before the GOE [78]. The data we present here can be consistent with a Mn-oxidizing transitional stage, but does not support an early Paleoproterozoic ancestral photosystem core duplication event. An alternative speculative scenario that could reconcile the Koegas Subgroup deposits with phylogenetics and other geochemical data [84], is that Mn oxidation in this locality was catalysed by a PSII using an atypical D1 form similar or ancestral to the rogue D1 or the ChlF proteins as recently suggested by Chernev et al. [82], but derived from water-splitting variants. It is also plausible that this occurred as a transitional stage towards the emergence of heterotrophy in cyanobacterial ancestors—even perhaps in any of the direct ancestors of MSV—under the selection pressure of other competing lineages of photosynthetic Cyanobacteria, linked to ecological changes occurring around the GOE [85,86].

The data presented here and in [19] suggest that water oxidation antedated the core duplications. We have taken this evidence before at face value to envision a water-splitting homodimeric photosystem that could perform catalysis on both branches of the RC. Such a system, however short-lived or inefficient, raises questions about the actual mechanism of catalysis [52]. For example, Fischer et al. considered the idea of a homodimeric water-splitting photosystem “highly unlikely” [87], because it would “require the coordination of eight electron transfers”. This thinking implicitly assumes that water oxidation can only occur through a mechanism that is identical to that of PSII, extrapolated to both sides of the RC, at the same time. This is but one of several scenarios that are consistent with water oxidation prior to core duplication. For example, partial oxidation of water to hydrogen peroxide is a plausible evolutionary transition before the full catalytic cycle of PSII was completed. Hydrogen peroxide release from partially oxidized water can occur in the lower oxidized states of the catalytic cycle in centres that have been perturbed by depletion of Ca^2+^ or Cl^−^ [88,89], removal of the extrinsic polypeptides [90], or by heating a sample [91]. Alternatively, a homodimeric system does not necessarily imply entirely homodimeric function. Like PSII, oxidation of Mn on one side of the RC could create asymmetric electrostatic effects on the photochemical pigments, P [92], and the quinone acceptor [93] (Fig. 8). In addition, the rate of Y_Z_ oxidation by P^+^ accelerates by two orders of magnitude in the presence of an assembled cluster [94]. This *acquired* asymmetry in a homodimeric photosystem upon Mn oxidation could shift redox equilibria increasing the probability of further oxidation events on one side relative to the other. It is conceivable that a catalytic site could turn over before the other is fully assembled, with the unassembled site assuming a supporting role similar to Y_D_ on D2 [95,96].

### 3.5 Duplications

**Fig. 9:**
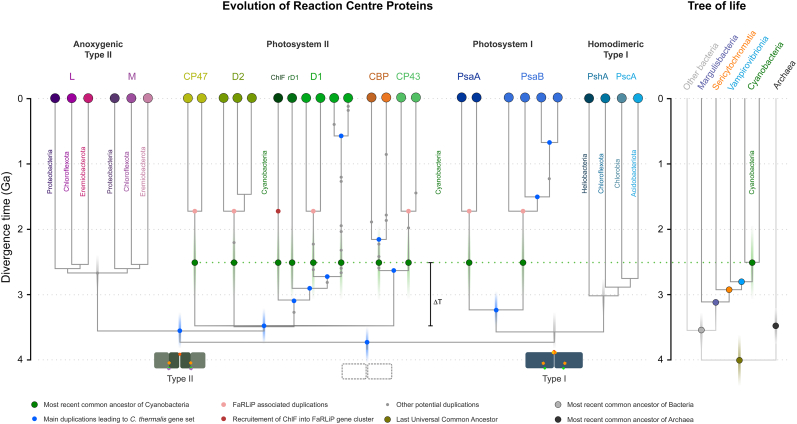
Schematic representation of the evolution of reaction centre proteins as a function of time. The section of the tree that focuses on PSII and PSI shows the RC gene content of the cyanobacterium *Chroococcidiopsis thermalis* PCC 7203. These are the product of several ancient and more recent gene duplications starting before the MRCA of Cyanobacteria (blue dotes at node positions). It has been suggested that the type I and II divergence was driven by a gene duplication event. The tree highlights how it is possible that oxygenic photosynthesis started deeper within the evolution of prokaryotes than currently understood (ΔT). We argue that the origin of water oxidation started prior to the CP43/D1 and CP47/D2 duplication and that the earliest type II photosystems were structurally, and potentially also functionally, like water-splitting PSII. Divergence times are meant to point out the relative occurrence of events, rather than exact ages, as described in the text, in ref. [19] for type II RC proteins and in [102] for type I RC proteins. The vertical transparent bars across nodes of interests highlight the uncertainty on the estimation of divergence times.

Before natural selection or drift can drive the heterodimerization of the photosystem, a gene duplication must occur first. The attention can thus be shifted away from asking if the duplication occurred before or after the origin of water oxidation, to asking why the RC gene duplicated to begin with. This is crucial because within the described diversity of photosynthetic organisms, all known RC gene duplications have occurred exclusively in the context of oxygenic photosynthesis. In particular, but not exclusively, duplications of the subunits that allow catalysis, D1 and CP43. In other words, and to the best of our knowledge, there are no known gene duplication events of PufL, PufM, PshA or any version of PscA described for any known anoxygenic phototroph (Fig. 9). Therefore, based on available observations, the only driving force known to lead to RC gene duplications is water oxidation to oxygen, and the subsequent need for sustained repair and protection against ROS-mediated damage. Duplicated subunits can then drift away from their main functions and become the canvas of novel adaptations in a process that has never stopped [2]. This is spectacularly exemplified in the evolution of FaRLiP [97], as chlorophyll *f* synthesis was likely enabled by a drifting D1 variant accumulating just two amino acid substitutions at the interface with CP43 [98].

Another conspicuous duplication is that leading to the heterodimeric core of PSI, which has been discussed to have occurred as an adaptation to oxygenic photosynthesis [[99], [100], [101], [102]]. In fact, Orf et al. [103] recently suggested that PSI started to accumulate adaptations to avoid singlet oxygen formation even before the duplication of the core. Furthermore, we have recently shown that the duplication leading to L and M, likely post-dated the evolution of oxygenic photosynthesis [19]. We have also discussed how the heterodimerization of the anoxygenic type II RC core could have occurred under pressure to minimize ROS-mediated damage [19].

The only other discussed, yet more ambiguous duplication is that which gave rise to type I and type II RC proteins, unanimously and implicitly assumed to have occurred before the origin of water oxidation [18,[104], [105], [106], [107]]. While we find compelling some of the rationales supporting a duplication at the origin of type I and II RCs, Cardona [77] raised issues with the implicit assumption because, like Mn-oxidizing photosynthesis without water oxidation, it invokes a transitional form of photosynthesis for which no evidence has been found within the known diversity of anoxygenic phototrophs. In this case, the emergence of anoxygenic photosynthesis using both RC types in series. Both phylogenetic and structural evidence converge towards a scenario in which photosynthetic water-splitting started at an early stage during the evolution of life and long before the rise of what we understand today as Cyanobacteria. We have shown evidence suggesting that the ancestral type II RC was similar to PSII, which is consistent with the hypothesis that the type I and type II split, likely enabled by a duplication event, indeed occurred in the context of the establishment of oxygenic photosynthesis [73,77], as illustrated in Fig. 9. We argue that this perspective offers more explanatory power than conventional scenarios. Not only it explains the structural and functional characteristics of PSII, but it also explains the unique coordination sphere of the Mn_4_CaO_5_ cluster in a manner consistent with phylogenetic relationships. Distinctly, this perspective does not require the involvement of speculative forms of photosynthesis. More importantly yet, it also provides a mechanism for the generation of genetic diversity via serial gene duplications from where photosystem innovation can occur, and, for which ample evidence exists within the genomes of most Cyanobacteria today.

We think it is plausible that there was never a discrete origin of photosynthesis, but that the process may trace back to abiogenic photochemical reactions, some of which may have resulted in the oxidation of water, and at the interface of nascent membranes, membrane proteins, photoactive tetrapyrroles and other inorganic cofactors: much in the same way that ribosomes may have originated at the interface of nascent genetics and protein synthesis [108]. A photosynthetic origin of life is not a new idea [[109], [110], [111]] and abiotic photosynthesis-like chemistry has been recently proposed to have occurred at Gale Crater on Mars [112], and to occur even on Earth [113].

## 4 Materials and methods

### 4.1 Sequence alignments and phylogenetic analysis

The first dataset was retrieved on the 31st of October 2017 to initiate this project. It included a total of 1389 type I RC, CP43 and CP47 protein sequences from the NCBI refseq database using BLAST. This dataset did not contain CBP proteins. A second dataset was retrieved on the 10th of October 2018 that included 392 CP43, 465 CBP proteins, and 375 CP47 subunits. 40 CP43 and 40 CP47 sequences from a diverse set of photosynthetic eukaryotes were selected manually and included. In addition, a selection of cyanobacterial and plastid CP43, CP47, AtpA (Alpha), AtpB (Beta), and FtsH were manually selected for an analysis of the rates of evolution as described below and in Supplementary Text S2.

A dataset of Alpha and Beta subunits belonging to cyanobacterial F-type ATP synthase were retrieved from the NCBI refseq on the 31st of August 2019. 507 and 529 cyanobacterial Alpha and Beta sequences were obtained respectively. We retrieved Alpha and Beta homologous for Margulisbacteria (19 and 18 sequences respectively), Sericytochromatia (4 and 2), and Vampirovibrionia (66 and 49) using AnnoTree [114] and searching with the KEGG codes K02111 (F-type Alpha) and K02112 (F-type Beta). A total of 111 AtpA (subunit A) and 176 AtpB (subunit B) from archaeal V-type ATP synthase were retrieved using the sequences from *Methanocaldococcus jannaschii* as BLAST queries.

On the same date (31st of August 2019), a dataset of 366 bacterial RNA polymerase subunit β (RpoB) were collected from the NCBI refseq. We focused on Cyanobacteria (65 sequences) and other phyla known to contain phototrophic representatives, as well as for their potential for allocating calibration points as described below. These included sequences from Firmicutes (32), Chloroflexota (32), Proteobacteria (102), Acidobacteriota (34), Chlorobia (26), Gemmatimonadetes (3), Aquifecae (12) and Thermotogae (24). Sequences for *Candidatus* Eremiobacterota (2) recently reported to include phototrophic representatives [115], Margulisbacteria (13), Sericytochromatia (2), and Vampirovibrionia (11) were obtained from AnnoTree using KEGG code K03043 as query. In addition, three extra margulisbacterial RpoB sequences were collected: from *Termititenax persephonae* and *Termititenax aidoneus* retrieved from the refseq database, and from margulisbacterium *Candidatus* Ruthmannia eludens obtained from https://www.ebi.ac.uk/ena/data/view/PRJEB30343 [7].

A second dataset of RpoB subunits containing only sequences from Cyanobacteria and MSV, in addition to sequences from Archaea was also used. To retrieve the archaeal sequences the subunit B′ (RpoB1) of *Methanocaldococcus jannaschii* was used as a BLAST query. A total 213 sequences across the entire diversity of Archaea were obtained. Two version of these were observed, those with split B (RpoB1 and RpoB2) and those with full-length B subunits: from the latter, 112 full-length B subunits were selected for molecular clock analysis. Finally, we also prepared a dataset of concatenated ribosomal proteins containing 2596 aligned sites. This dataset was obtained from an independent study [39] and we selected a total of 157 sequences: 56 cyanobacterial, 14 sequences from Vampirovibrionia, 9 from Margulisbacteria, 9 from Thermotogae and 78 from Archaea.

Sequences were aligned with Clustal Omega using 5 combined guided trees and Hidden Markov Model Iterations [116]. Given that RpoB sequences are known to contain many clade specific indels [28], we further processed this particular dataset using Gblocks [117] to remove these indels, allowing smaller final blocks, gap positions within the final blocks, and less strict flanking positions. This procedure left a total of 903 well-aligned sites for the dataset with only bacterial sequences and 788 for the dataset with archaeal sequences. The dataset of concatenated ribosomal proteins was made available by the authors aligned: after curating for unused taxa, remaining gap-only sites were removed without further processing.

Maximum Likelihood phylogenetic analysis was performed with the PhyML online service using the Smart Model Selection mode implementing the Bayesian Information Criterion for parameter selection [118,119]. Tree searching operations were calculated with the Nearest Neighbour Interchange model. Support was computed using the average likelihood ratio test [120]. Trees were visualized with Dendroscope V3.5.9 [121].

### 4.2 Distance estimation

Distance estimation was performed as a straightforward and intuitive approach to detect possible variations in the rate of sequence change. Distance trees were plotted using BioNJ [122] as implemented in Seaview V4.7 [123] using observed distances and 100 bootstrap replicates. Within-group mean distances were calculated for RpoB, Alpha, Beta, and the ribosomal dataset using three different substitution models as implemented in the package MEGA-X [124]: no. of differences, a Poisson model, and a JTT model. A gamma distribution to model rates among sites was used with a parameter of 1.00 and 500 bootstrap replicates.

To compare the level of sequence identity between RC proteins, two datasets of 10 random amino acid sequences were generated using the Sequence Manipulation Suit [125]. The datasets contained sequences of 350 and 750 residues. These were independently aligned as described above, resulting in 45 pairwise sequence identity comparisons for each dataset. These random sequence datasets were used as a rough minimum threshold of identity. Alignments of RC proteins were generated using three representative sequences spanning known diversity. Cyanobacterial CP43, CP47, standard D1 and D2 sequences were from *Gloeobacter violaceus*, *Stanieria cyanosphaera*, and *Nostoc* sp. PCC 7120; Heliobacterial PshA from *Heliobacterium modesticaldum*, *Heliobacillus mobilis*, and *Heliorestis convoluta*; PscA from *Chlorobium tepidum*, *Prosthecochloris aestuarii*, *Chlorobium* sp. GBchlB, *Chloracidobacterium thermophilum* and *Chloracidobacterium* sp. CP2-5A; Proteobacterial L and M from *Methylobacterium* sp. 88A, *Roseivivax halotolerans*, and *Blastochloris sulfoviridis*; and L and M from *Chloroflexus* sp. Y-400-fl, *Roseiflexus castenholzii*, and *Oscillochloris trichoides*.

### 4.3 Rates and molecular clocks

To measure the rates of amino acid substitution of PSII, ATP synthase and FtsH subunits under varying evolutionary scenarios we followed a methodology modified from Cardona et al. [19] and as detailed in Supplementary Text S2 *Quantification of rates of evolution*, *Rationale* and *Method*. For this experiment, all used rates of amino acid substitutions and divergence times were calculated with Phylobayes 3.3. We applied a log normal autocorrelated model under the CAT+Γ non-parametric model of amino acid substitution and a uniform distribution of equilibrium frequencies. We preferred a CAT model instead of a CAT+GTR as the latter only outperforms the former on sequence alignments of over 1000 sites and it is also much less computationally expensive [126]. Four discrete categories for the gamma distribution were used and four chains were executed in parallel through all experiments carried out in this study. The rates of evolution were retrieved from the output files of Phylobayes and expressed as amino acid substitutions per site per Ga. These rates are calculated by the software as described by the developers elsewhere [127,128].

To have a closer understanding of the spans of time between Cyanobacteria and MSV and their associated rates of evolution under different evolutionary scenarios, we applied a molecular clock to the phylogeny of RpoB sequences described above. We implemented 12 calibrations and several root priors, which are described and illustrated in Supplementary Text S3 *Calibrating the RpoB* *and* *ribosomal proteins phylogenies*. Molecular clocks were calculated as described above. We also compared a range of models and parameters including an uncorrelated gamma model of rates of amino acid substitution, +GTR, and the application of birth-death priors combined with soft bounds. When soft bounds were used a 2.5% tail probability was allowed to fall outside the minimum and maximum boundary, or 5% in the case of a single boundary. The full set of comparisons is presented in Supplementary Figs. S6 and S7.

### 4.4 Ancestral sequence reconstruction

Ancestral sequence reconstruction (ASR) of D1 and D2 amino acid sequences was carried out with a dataset collected on the 17th of November 2017. Duplicates and partial sequences were removed, leaving 755 D1 and 248 D2 sequences. CD-HIT [129] was used to remove sequences with greater than 92% sequence identity to create a representative sample. The L and M RC subunits from 5 strains of Proteobacteria were used as outgroup. The final alignment did not include the atypical variant D1 sequence from *Gloeobacter kilaueensis* (NCBI accession AGY58976.1) as this showed an unstable phylogenetic position in this dataset. Maximum likelihood trees used as input for ASR were computed with PhyML using Smart Model Selection [119]. The LG substitution model with observed amino acid frequencies and four gamma rate categories exhibited the best log likelihood (LG+Γ+F) (Supplementary Table S7). We used the top four models for tree reconstruction (LG+Γ+F, LG+Γ+I+F, LG+Γ and LG+Γ+I) in addition to another tree computed using the WAG substitution model with observed amino acid frequencies and four gamma rate categories (WAG+Γ+F). These trees were used as input trees to calculate maximum likelihood ancestral states at each site for the node corresponding to the homodimeric reaction protein, D0. Three ASR programs were used for the reconstructions: FastML [130], Lazarus [131] (a set of Python scripts which wraps PAML [132]) and MEGA-7 [133]. The substitution model used by all three programs corresponded to the substitution model used by PhyML to generate the specific input tree. Branch lengths of the trees were fixed as these were already estimated for the input trees. In FastML, a maximum likelihood method of indel reconstruction using a probability cut-off of 0.7 was used. In MEGA-7, all sites were used for analysis with no branch swap filter. In Lazarus, the ‘—gapcorrect’ option was used to parsimoniously place indels.

### 4.5 Structural analysis

The following crystal structures were used in this work: the crystal structure of PSII from *Thermosynechococcus vulcanus* and *Cyanidium caldarium*, PDB ID: 3wu2 and 4yuu respectively [14,134]; the anoxygenic type II RC of *Thermochromatium tepidum*, PDB ID: 5y5s [135]; the homodimeric type I RC from *Heliobacterium modesticaldum*, 8v5k [136]; PSI from *Thermosynechococcus elongatus*, PDB ID: 1jb0 [137]; the cryo-EM IsiA structure from *Synechocystis* sp. PCC 6803 [138]. Structures were visualized using Pymol™ V. 1.8.2.2 (Schrodinger, LLC) and structural overlaps were carried out with the CEAlign plugin [139].

## Declaration of competing interest

The authors declare that they have no known competing financial interests or personal relationships that could have appeared to influence the work reported in this paper.

## Appendix A Supplementary data

Supplementary material: sequence alignments and trees Image 1

Supplementary figures and text Image 2

Supplementary tables Image 3

## References

[1] Sánchez-Baracaldo P., Cardona T. (2020). On the origin of oxygenic photosynthesis and Cyanobacteria. New Phytol..

[2] Cardona T., Murray J.W., Rutherford A.W. (2015). Origin and evolution of water oxidation before the last common ancestor of the Cyanobacteria. Mol. Biol. Evol..

[3] Di Rienzi S.C., Sharon I., Wrighton K.C., Koren O., Hug L.A., Thomas B.C., Goodrich J.K., Bell J.T., Spector T.D., Banfield J.F., Ley R.E. (2013). The human gut and groundwater harbor non-photosynthetic bacteria belonging to a new candidate phylum sibling to Cyanobacteria. Elife.

[4] Soo R.M., Hemp J., Hugenholtz P. (2019). Evolution of photosynthesis and aerobic respiration in the cyanobacteria. Free Radic. Biol. Med..

[5] Soo R.M., Hemp J., Parks D.H., Fischer W.W., Hugenholtz P. (2017). On the origins of oxygenic photosynthesis and aerobic respiration in Cyanobacteria. Science.

[6] Anantharaman K., Brown C.T., Hug L.A., Sharon I., Castelle C.J., Probst A.J., Thomas B.C., Singh A., Wilkins M.J., Karaoz U., Brodie E.L., Williams K.H., Hubbard S.S., Banfield J.F. (2016). Thousands of microbial genomes shed light on interconnected biogeochemical processes in an aquifer system. Nat. Commun..

[7] Gruber-Vodicka H.R., Leisch N., Kleiner M., Hinzke T., Liebeke M., McFall-Ngai M., Hadfield M.G., Dubilier N. (2019). Two intracellular and cell type-specific bacterial symbionts in the placozoan Trichoplax H2. Nat. Microbiol..

[8] Utami Y.D., Kuwahara H., Igai K., Murakami T., Sugaya K., Morikawa T., Nagura Y., Yuki M., Deevong P., Inoue T., Kihara K., Lo N., Yamada A., Ohkuma M., Hongoh Y. (2019). Genome analyses of uncultured TG2/ZB3 bacteria in ‘Margulisbacteria’ specifically attached to ectosymbiotic spirochetes of protists in the termite gut. ISME J..

[9] Soo R.M., Woodcroft B.J., Parks D.H., Tyson G.W., Hugenholtz P. (2015). Back from the dead; the curious tale of the predatory cyanobacterium Vampirovibrio chlorellavorus. PeerJ.

[10] Carnevali P.B.M., Schulz F., Castelle C.J., Kantor R.S., Shih P.M., Sharon I., Santini J.M., Olm M.R., Amano Y., Thomas B.C., Anantharaman K., Burstein D., Becraft E.D., Stepanauskas R., Woyke T., Banfield J.F. (2019). Hydrogen-based metabolism as an ancestral trait in lineages sibling to the Cyanobacteria. Nat. Commun..

[11] Shih P.M., Hemp J., Ward L.M., Matzke N.J., Fischer W.W. (2017). Crown group Oxyphotobacteria postdate the rise of oxygen. Geobiology.

[12] Magnabosco C., Moore K.R., Wolfe J.M., Fournier G.P. (2018). Dating phototrophic microbial lineages with reticulate gene histories. Geobiology.

[13] Schirrmeister B.E., Sánchez-Baracaldo P., Wacey D. (2016). Cyanobacterial evolution during the Precambrian. Int. J. Astrobiol..

[14] Umena, Y., K. Kawakami, J.R. Shen, and N. Kamiya, Crystal structure of oxygen-evolving Photosystem II at a resolution of 1.9 Å. Nature, 2011. 473: 55–60. DOI:10.1038/nature09913. 21499260

[15] Ferreira K.N., Iverson T.M., Maghlaoui K., Barber J., Iwata S. (2004). Architecture of the photosynthetic oxygen-evolving center. Science.

[16] Cardona T., Sedoud A., Cox N., Rutherford A.W. (2012). Charge separation in Photosystem II: a comparative and evolutionary overview. Biochim. Biophys. Acta.

[17] Rutherford, A.W., T. Mattioli, and W. Nitschke, The FeS-type photosystems and the evolution of photosynthetic reaction centers, in *Origin and evolution of biological energy conversion*, H. Baltscheffsky, Editor. 1996, VCH: New York, N. Y. 177-203.

[18] Rutherford, A.W. and W. Nitschke, Photosystem II and the quinone–iron-containing reaction centers, in *Origin and evolution of biological energy conversion*, H. Baltscheffsky, Editor. 1996, VCH: New York, N. Y. 143–175.

[19] Cardona T., Sánchez-Baracaldo P., Rutherford A.W., Larkum A.W.D. (2019). Early Archean origin of Photosystem II. Geobiology.

[20] Cardona T., Rutherford A.W. (2019). Evolution of photochemical reaction centres: more twists?. Trends Plant Sci..

[21] Mulkidjanian A.Y., Makarova K.S., Galperin M.Y., Koonin E.V. (2007). Inventing the dynamo machine: the evolution of the F-type and V-type ATPases. Nat. Rev. Microbiol..

[22] Gogarten J.P., Kibak H., Dittrich P., Taiz L., Bowman E.J., Bowman B.J., Manolson M.F., Poole R.J., Date T., Oshima T., Konishi J., Denda K., Yoshida M. (1989). Evolution of the vacuolar H^+^-ATPase: implications for the origin of eukaryotes. Proc. Natl. Acad. Sci. U. S. A..

[23] Lane N., Allen J.F., Martin W. (2010). How did LUCA make a living? Chemiosmosis in the origin of life. Bioessays.

[24] Ouzounis C.A., Kunin V., Darzentas N., Goldovsky L. (2006). A minimal estimate for the gene content of the last universal common ancestor: exobiology from a terrestrial perspective. Res. Microbiol..

[25] Muller V., Gruber G. (2003). ATP synthases: structure, function and evolution of unique energy converters. Cell. Mol. Life Sci..

[26] Werner F., Grohmann D. (2011). Evolution of multisubunit RNA polymerases in the three domains of life. Nat. Rev. Microbiol..

[27] Kyrpides N., Overbeek R., Ouzounis C. (1999). Universal protein families and the functional content of the Last Universal Common Ancestor. J. Mol. Evol..

[28] Lane W.J., Darst S.A. (2010). Molecular evolution of multisubunit RNA polymerases: sequence analysis. J. Mol. Biol..

[29] Harris J.K., Kelley S.T., Spiegelman G.B., Pace N.R. (2003). The genetic core of the universal ancestor. Genome Res..

[30] Chen M., Zhang Y., Blankenship R.E. (2008). Nomenclature for membrane-bound light-harvesting complexes of cyanobacteria. Photosynth. Res..

[31] Chen M., Hiller R.G., Howe C.J., Larkum A.W.D. (2005). Unique origin and lateral transfer of prokaryotic chlorophyll-*b* and chlorophyll-*d* light-harvesting systems. Mol. Biol. Evol..

[32] Murray J.W. (2012). Sequence variation at the oxygen-evolving centre of Photosystem II: a new class of ‘rogue’ cyanobacterial D1 proteins. Photosynth. Res..

[33] Ho, M.Y., G. Shen, D.P. Canniffe, C. Zhao, and D.A. Bryant, *Light-dependent chlorophyll f synthase is a highly divergent paralog of PsbA of Photosystem II.* Science, 2016. 353: aaf9178. DOI:10.1126/science.aaf9178. 27386923

[34] Gan, F., G. Shen, and D.A. Bryant, *Occurrence of far-red light photoacclimation (FaRLiP) in diverse cyanobacteria.* Life (Basel), 2014. 5: 4–24. DOI:10.3390/life5010004. PMC4390838 25551681

[35] Cardona T. (2015). A fresh look at the evolution and diversification of photochemical reaction centers. Photosynth. Res..

[36] Cardona T. (2016). Reconstructing the origin of oxygenic photosynthesis: do assembly and photoactivation recapitulate evolution?. Front. Plant Sci..

[37] Dibrova D.V., Galperin M.Y., Mulkidjanian A.Y. (2010). Characterization of the N-ATPase, a distinct, laterally transferred Na^+^-translocating form of the bacterial F-type membrane ATPase. Bioinformatics.

[38] Battistuzzi F.U., Feijao A., Hedges S.B. (2004). A genomic timescale of prokaryote evolution: insights into the origin of methanogenesis, phototrophy, and the colonization of land. BMC Evol. Biol..

[39] Hug L.A., Baker B.J., Anantharaman K., Brown C.T., Probst A.J., Castelle C.J., Butterfield C.N., Hernsdorf A.W., Amano Y., Ise K., Suzuki Y., Dudek N., Relman D.A., Finstad K.M., Amundson R., Thomas B.C., Banfield J.F. (2016). A new view of the tree of life. Nat. Microbiol..

[40] Parks D.H., Rinke C., Chuvochina M., Chaumeil P.A., Woodcroft B.J., Evans P.N., Hugenholtz P., Tyson G.W. (2017). Recovery of nearly 8,000 metagenome-assembled genomes substantially expands the tree of life. Nat. Microbiol..

[41] Soo R.M., Skennerton C.T., Sekiguchi Y., Imelfort M., Paech S.J., Dennis P.G., Steen J.A., Parks D.H., Tyson G.W., Hugenholtz P. (2014). An expanded genomic representation of the phylum Cyanobacteria. Genome Biol. Evol..

[42] López-García, P. and D. Moreira, Physical connections: prokaryotes parasitizing their kin. Environ. Microbiol. Rep., 2020. DOI:10.1111/1758-2229.12910. 33225570

[43] Castelle C.J., Brown C.T., Anantharaman K., Probst A.J., Huang R.H., Banfield J.F. (2018). Biosynthetic capacity, metabolic variety and unusual biology in the CPR and DPANN radiations. Nat. Rev. Microbiol..

[44] Coleman, G.A., A.A. Davín, T. Mahendrarajah, A. Spang, P. Hugenholtz, G.J. Szöllősi, and T.A. Williams, A rooted phylogeny resolves early bacterial evolution. bioRxiv, 2020: 2020.07.15.205187. DOI:10.1101/2020.07.15.205187. 33958449

[45] Meheust R., Burstein D., Castelle C.J., Banfield J.F. (2019). The distinction of CPR bacteria from other bacteria based on protein family content. Nat. Commun..

[46] Betts H.C., Puttick M.N., Clark J.W., Williams T.A., Donoghue P.C.J., Pisani D. (2018). Integrated genomic and fossil evidence illuminates life's early evolution and eukaryote origin. Nat. Ecol. Evol..

[47] Shao S., Cardona T., Nixon P.J. (2018). Early emergence of the FtsH proteases involved in photosystem II repair. Photosynthetica.

[48] Innan H., Kondrashov F. (2010). The evolution of gene duplications: classifying and distinguishing between models. Nat. Rev. Genet..

[49] Lynch M., Conery J.S. (2000). The evolutionary fate and consequences of duplicate genes. Science.

[50] Ho S.Y.W., Duchene S. (2014). Molecular-clock methods for estimating evolutionary rates and timescales. Mol. Ecol..

[51] Tice M.M., Lowe D.R. (2004). Photosynthetic microbial mats in the 3,416-Myr-old ocean. Nature.

[52] Rutherford A.W., Faller P. (2003). Photosystem II: evolutionary perspectives. Philos. Trans. Royal Soc. B.

[53] Nixon P.J., Diner B.A. (1992). Aspartate 170 of the Photosystem II reaction center polypeptide D1 is involved in the assembly of the oxygen-evolving manganese cluster. Biochemistry.

[54] Adachi Y., Kuroda H., Yukawa Y., Sugiura M. (2012). Translation of partially overlapping psbD-psbC mRNAs in chloroplasts: the role of 5′-processing and translational coupling. Nucleic Acids Res..

[55] Carpenter, S.D., J. Charite, B. Eggers, and W.F. Vermaas, The psbC start codon in Synechocystis sp. PCC 6803. FEBS Lett., 1990. 260: 135–7. DOI:10.1016/0014-5793(90)80085-w. 2105233

[56] Chisholm D., Williams J.G. (1988). Nucleotide sequence of psbC, the gene encoding the CP-43 chlorophyll a-binding protein of Photosystem II, in the cyanobacterium Synechocystis 6803. Plant Mol. Biol..

[57] Garcia-Pichel F., Lombard J., Soule T., Dunaj S., Wu S.H., Wojciechowski M.F. (2019). Timing the evolutionary advent of cyanobacteria and the later great oxidation event using gene phylogenies of a sunscreen. Mbio.

[58] Cockell C.S. (2000). Ultraviolet radiation and the photobiology of earth’s early oceans. Orig. Life Evol. Biospheres.

[59] Lewis C.A., Crayle J., Zhou S.T., Swanstrom R., Wolfenden R. (2016). Cytosine deamination and the precipitous decline of spontaneous mutation during Earth’s history. Proc. Natl. Acad. Sci. U. S. A..

[60] Levy M., Miller S.L. (1998). The stability of the RNA bases: implications for the origin of life. Proc. Natl. Acad. Sci. U. S. A..

[61] Eisen, J.A. and P.C. Hanawalt, A phylogenomic study of DNA repair genes, proteins, and processes. Mutat. Res./DNA Repair, 1999. 435: 171–213. DOI:10.1016/s0921-8777(99)00050-6. PMC3158673 10606811

[62] Koonin, E.V. and M.Y. Galperin, The major transitions in evolution: A comparative genomic perspective in Sequence — Evolution — Function: Computational Approaches in Comparative Genomics. 2003, Springer-Science+Business Media, B.V>. 252–292. DOI:10.1007/978-1-4757-3783-7.

[63] Woese C. (1998). The universal ancestor. Proc. Natl. Acad. Sci. U. S. A..

[64] David L.A., Alm E.J. (2011). Rapid evolutionary innovation during an Archaean genetic expansion. Nature.

[65] Marin J., Battistuzzi F.U., Brown A.C., Hedges S.B. (2017). The timetree of prokaryotes: new insights into their evolution and speciation. Mol. Biol. Evol..

[66] Zhu, Q., U. Mai, W. Pfeiffer, S. Janssen, F. Asnicar, J.G. Sanders, P. Belda-Ferre, G.A. Al-Ghalith, E. Kopylova, D. McDonald, T. Kosciolek, J.B. Yin, S. Huang, N. Salam, J.-Y. Jiao, Z. Wu, Z.Z. Xu, K. Cantrell, Y. Yang, E. Sayyari, M. Rabiee, J.T. Morton, S. Podell, D. Knights, W.-J. Li, C. Huttenhower, N. Segata, L. Smarr, S. Mirarab, and R. Knight, Phylogenomics of 10,575 genomes reveals evolutionary proximity between domains Bacteria and Archaea. Nat. Commun., 2019. 10: 5477. DOI:10.1038/s41467-019-13443-4. PMC6889312 31792218

[67] Berkemer S.J., McGlynn S.E. (2020). A New Analysis of Archaea–Bacteria Domain Separation: Variable Phylogenetic Distance and the Tempo of Early Evolution. Mol. Biol. Evol..

[68] Moody, E.R.R., T.A. Mahendrarajah, N. Dombrowski, J.W. Clark, C. Petitjean, P. Offre, G.J. Szollosi, A. Spang, and T.A. Williams, Universal markers support a long inter-domain branch between Archaea and Bacteria. bioRxiv, 2021: 2021.01.19.427276. DOI:10.1101/2021.01.19.427276.

[69] Homann, M., C. Heubeck, A. Airo, and M.M. Tice, Morphological adaptations of 3.22 Ga-old tufted microbial mats to Archean coastal habitats (Moodies Group, Barberton Greenstone Belt, South Africa). Precambrian Res., 2015. 266: 47–64. DOI:10.1016/j.precamres.2015.04.018.

[70] Alcott L.J., Mills B.J.W., Poulton S.W. (2019). Stepwise Earth oxygenation is an inherent property of global biogeochemical cycling. Science.

[71] Kadoya S., Catling D.C., Nicklas R.W., Puchtel I.S., Anbar A.D. (2020). Mantle data imply a decline of oxidizable volcanic gases could have triggered the great oxidation. Nat. Commun..

[72] Reinhard C.T., Planavsky N.J. (2020). Biogeochemical controls on the redox evolution of Earth’s oceans and atmosphere. Elements.

[73] Cardona T. (2017). Photosystem II is a chimera of reaction centers. J. Mol. Evol..

[74] Chen, J.-H., H. Wu, C. Xu, X.-C. Liu, Z. Huang, S. Chang, W. Wang, G. Han, T. Kuang, J.-R. Shen, and X. Zhang, Architecture of the photosynthetic complex from a green sulfur bacterium. Science, 2020. 370: eabb6350. DOI:10.1126/science.abb6350. 33214250

[75] Trinugroho J.P., Beckova M., Shao S., Yu J., Zhao Z., Murray J.W., Sobotka R., Komenda J., Nixon P.J. (2020). Chlorophyll f synthesis by a super-rogue photosystem II complex. Nat Plants.

[76] Wegener K.M., Nagarajan A., Pakrasi H.B. (2015). An atypical psbA gene encodes a sentinel D1 protein to form a physiologically relevant inactive Photosystem II complex in Cyanobacteria. J. Biol. Chem..

[77] Cardona T. (2019). Thinking twice about the evolution of photosynthesis. Open Biol..

[78] Fischer W.W., Hemp J., Johnson J.E. (2015). Manganese and the evolution of photosynthesis. Orig. Life Evol. Biospheres.

[79] Allen J.F., Martin W. (2007). Evolutionary biology - out of thin air. Nature.

[80] Allen J.P., Olson T.L., Oyala P., Lee W.J., Tufts A.A., Williams J.C. (2012). Light-driven oxygen production from superoxide by Mn-binding bacterial reaction centers. Proc. Natl. Acad. Sci. U. S. A..

[81] Williamson A., Conlan B., Hillier W., Wydrzynski T. (2011). The evolution of Photosystem II: insights into the past and future. Photosynth. Res..

[82] Chernev P., Fischer S., Hoffmann J., Oliver N., Assunção R., Yu B., Burnap R.L., Zaharieva I., Nürnberg D.J., Haumann M., Dau H. (2020). Light-driven formation of manganese oxide by today’s photosystem II supports evolutionarily ancient manganese-oxidizing photosynthesis. Nat. Commun..

[83] Johnson J.E., Webb S.M., Thomas K., Ono S., Kirschvink J.L., Fischer W.W. (2013). Manganese-oxidizing photosynthesis before the rise of cyanobacteria. Proc. Natl. Acad. Sci. U. S. A..

[84] Planavsky N.J., Asael D., Hofmann A., Reinhard C.T., Lalonde S.V., Knudsen A., Wang X., Ossa Ossa F., Pecoits E., Smith A.J.B., Beukes N.J., Bekker A., Johnson T.M., Konhauser K.O., Lyons T.W., Rouxel O.J. (2014). Evidence for oxygenic photosynthesis half a billion years before the great oxidation event. Nat. Geosci..

[85] Hodgskiss M.S.W., Crockford P.W., Peng Y.B., Wing B.A., Horner T.J. (2019). A productivity collapse to end Earth’s great oxidation. Proc. Natl. Acad. Sci. U. S. A..

[86] Bekker A., Holland H.D. (2012). Oxygen overshoot and recovery during the early Paleoproterozoic. Earth Planet. Sci. Lett..

[87] Fischer W.W., Hemp J., Johnson J.E. (2016). Evolution of oxygenic photosynthesis. Annu. Rev. Earth Planet. Sci..

[88] Fine P.L., Frasch W.D. (1992). The oxygen-evolving complex requires chloride to prevent hydrogen peroxide formation. Biochemistry.

[89] Arato A., Bondarava N., Krieger-Liszkay A. (2004). Production of reactive oxygen species in chloride- and calcium-depleted photosystem II and their involvement in photoinhibition. Bba-Bioenergetics.

[90] Hillier W., Wydrzynski T. (1993). Increases in peroxide formation by the Photosystem II oxygen evolving reactions upon removal of the extrinsic 16, 22 and 33 kDa proteins are reversed by CaCl2 addition. Photosynth. Res..

[91] Thompson L.K., Blaylock R., Sturtevant J.M., Brudvig G.W. (1989). Molecular basis of the heat denaturation of photosystem II. Biochemistry.

[92] Ishikita H., Saenger W., Biesiadka J., Loll B., Knapp E.W. (2006). How photosynthetic reaction centers control oxidation power in chlorophyll pairs P680, P700, and P870. Proc. Natl. Acad. Sci. U. S. A..

[93] Johnson G.N., Rutherford A.W., Krieger A. (1995). A change in the midpoint potential of the quinone Q(a) in Photosystem II associated with photoactivation of oxygen evolution. Biochim. Biophys. Acta.

[94] Schlodder E., Meyer B. (1987). pH dependence of oxygen evolution and reduction kinetics of photooxidized chlorophyll a_II_ (P-680) in Photosystem II particles from Synechococcus sp. Biochim. Biophys. Acta.

[95] Rutherford A.W., Boussac A., Faller P. (2004). The stable tyrosyl radical in photosystem II: why D?. Biochim. Biophys. Acta.

[96] Styring S., Sjoholm J., Mamedov F. (2012). Two tyrosines that changed the world: interfacing the oxidizing power of photochemistry to water splitting in photosystem II. Bba-Bioenergetics.

[97] Gan F., Zhang S., Rockwell N.C., Martin S.S., Lagarias J.C., Bryant D.A. (2014). Extensive remodeling of a cyanobacterial photosynthetic apparatus in far-red light. Science.

[98] Trinugroho, J.P., M. Beckova, S.X. Shao, J.F. Yu, Z.Y. Zhao, J.W. Murray, R. Sobotka, J. Komenda, and P.J. Nixon, Chlorophyll f synthesis by a super-rogue photosystem II complex. Nature Plants, 2020. 6: 238−+. DOI:10.1038/s41477-020-0616-4. 32170286

[99] Rutherford A.W., Osyczka A., Rappaport F. (2012). Back-reactions, short-circuits, leaks and other energy wasteful reactions in biological electron transfer: redox tuning to survive life in O_2_. FEBS Lett..

[100] Ben-Shem A., Frolow F., Nelson N. (2004). Evolution of photosystem I - from symmetry through pseudosymmetry to asymmetry. FEBS Lett..

[101] Jagannathan, B., G.Z. Shen, and J.H. Golbeck, The evolution of Type I reaction centers: the response to oxygenic photosynthesis, in Functional genomics and evolution of photosynthetic systems, R.L. Burnap and W. Vermaas, Editors. 2012, Springer Science: Dordrecht. 285-316.

[102] Cardona T. (2018). Early Archean origin of heterodimeric Photosystem I. Heliyon.

[103] Orf, G.S., C. Gisriel, and K.E. Redding, Evolution of photosynthetic reaction centers: insights from the structure of the heliobacterial reaction center. Photosynth. Res., 2018. DOI:10.1007/s11120-018-0503-2. 29603081

[104] Allen J.F. (2005). A redox switch hypothesis for the origin of two light reactions in photosynthesis. FEBS Lett..

[105] Sousa F.L., Shavit-Grievink L., Allen J.F., Martin W.F. (2013). Chlorophyll biosynthesis gene evolution indicates photosystem gene duplication, not photosystem merger, at the origin of oxygenic photosynthesis. Genome Biol. Evol..

[106] Olson J.M., Pierson B.K. (1987). Origin and evolution of photosynthetic reaction centers. Orig. Life Evol. Biospheres.

[107] Mulkidjanian A.Y., Koonin E.V., Makarova K.S., Mekhedov S.L., Sorokin A., Wolf Y.I., Dufresne A., Partensky F., Burd H., Kaznadzey D., Haselkorn R., Galperin M.Y. (2006). The cyanobacterial genome core and the origin of photosynthesis. Proc. Natl. Acad. Sci. U. S. A..

[108] Petrov A.S., Bernier C.R., Hsiao C.L., Norris A.M., Kovacs N.A., Waterbury C.C., Stepanov V.G., Harvey S.C., Fox G.E., Wartell R.M., Hud N.V., Williams L.D. (2014). Evolution of the ribosome at atomic resolution. Proc. Natl. Acad. Sci. U. S. A..

[109] Granick S. (1957). Speculations on the origins and evolution of photosynthesis. Ann. N. Y. Acad. Sci..

[110] Mauzerall D. (1992). Light, iron, Sam Granick and the origin of life. Photosynth. Res..

[111] Mulkidjanian A.Y., Bychkov A.Y., Dibrova D.V., Galperin M.Y., Koonin E.V. (2012). Origin of first cells at terrestrial, anoxic geothermal fields. Proc. Natl. Acad. Sci. U. S. A..

[112] Franz, H.B., P.R. Mahaffy, C.R. Webster, G.J. Flesch, E. Raaen, C. Freissinet, S.K. Atreya, C.H. House, A.C. McAdam, C.A. Knudson, P.D. Archer, J.C. Stern, A. Steele, B. Sutter, J.L. Eigenbrode, D.P. Glavin, J.M.T. Lewis, C.A. Malespin, M. Millan, D.W. Ming, R. Navarro-Gonzalez, and R.E. Summons, Indigenous and exogenous organics and surface-atmosphere cycling inferred from carbon and oxygen isotopes at Gale crater. Nat Astron, 2020. DOI:10.1038/s41550-019-0990-x.

[113] Lu A.H., Li Y., Ding H.R., Xu X.M., Li Y.Z., Ren G.P., Liang J., Liu Y.W., Hong H., Chen N., Chu S.Q., Liu F.F., Wang H.R., Ding C., Wang C.Q., Lai Y., Liu J., Dick J., Liu K.H., Hochella M.F. (2019). Photoelectric conversion on Earth’s surface via widespread Fe- and Mn-mineral coatings. Proc. Natl. Acad. Sci. U. S. A..

[114] Mendler K., Chen H., Parks D.H., Lobb B., Hug L.A., Doxey A.C. (2019). AnnoTree: visualization and exploration of a functionally annotated microbial tree of life. Nucleic Acids Res..

[115] Ward L.M., Cardona T., Holland-Moritz H. (2019). Evolutionary implications of anoxygenic phototrophy in the bacterial phylum Candidatus Eremiobacterota (WPS-2). Front. Microbiol..

[116] Sievers F., Wilm A., Dineen D., Gibson T.J., Karplus K., Li W.Z., Lopez R., McWilliam H., Remmert M., Soding J., Thompson J.D., Higgins D.G. (2011). Fast, scalable generation of high-quality protein multiple sequence alignments using Clustal Omega. Mol. Syst. Biol..

[117] Castresana J. (2000). Selection of conserved blocks from multiple alignments for their use in phylogenetic analysis. Mol. Biol. Evol..

[118] Guindon, S., J.F. Dufayard, V. Lefort, M. Anisimova, W. Hordijk, and O. Gascuel, New algorithms and methods to estimate maximum-likelihood phylogenies: assessing the performance of PhyML 3.0. Syst. Biol., 2010. 59: 307–21. DOI:10.1093/sysbio/syq010. 20525638

[119] Lefort V., Longueville J.E., Gascuel O. (2017). SMS: smart model selection in PhyML. Mol. Biol. Evol..

[120] Anisimova M., Gascuel O. (2006). Approximate likelihood-ratio test for branches: a fast, accurate, and powerful alternative. Syst. Biol..

[121] Huson D.H., Scornavacca C. (2012). Dendroscope 3: an interactive tool for rooted phylogenetic trees and networks. Syst. Biol..

[122] Gascuel O. (1997). BIONJ: an improved version of the NJ algorithm based on a simple model of sequence data. Mol. Biol. Evol..

[123] Gouy M., Guindon S., Gascuel O. (2010). SeaView version 4: a multiplatform graphical user interface for sequence alignment and phylogenetic tree building. Mol. Biol. Evol..

[124] Kumar S., Stecher G., Li M., Knyaz C., Tamura K. (2018). MEGA X: molecular evolutionary genetics analysis across computing platforms. Mol. Biol. Evol..

[125] Stothard P. (2000). The sequence manipulation suite: JavaScript programs for analyzing and formatting protein and DNA sequences. BioTechniques.

[126] Lartillot N., Lepage T., Blanquart S. (2009). PhyloBayes 3: a Bayesian software package for phylogenetic reconstruction and molecular dating. Bioinformatics.

[127] Lepage T., Bryant D., Philippe H., Lartillot N. (2007). A general comparison of relaxed molecular clock models. Mol. Biol. Evol..

[128] Kishino H., Thorne J.L., Bruno W.J. (2001). Performance of a divergence time estimation method under a probabilistic model of rate evolution. Mol. Biol. Evol..

[129] Li W., Godzik A. (2006). Cd-hit: a fast program for clustering and comparing large sets of protein or nucleotide sequences. Bioinformatics.

[130] Ashkenazy H., Penn O., Doron-Faigenboim A., Cohen O., Cannarozzi G., Zomer O., Pupko T. (2012). FastML: a web server for probabilistic reconstruction of ancestral sequences. Nucleic Acids Res..

[131] Hanson-Smith V., Kolaczkowski B., Thornton J.W. (2010). Robustness of ancestral sequence reconstruction to phylogenetic uncertainty. Mol. Biol. Evol..

[132] Yang Z.H. (2007). PAML 4: phylogenetic analysis by maximum likelihood. Mol. Biol. Evol..

[133] Kumar S., Stecher G., Tamura K. (2016). MEGA7: molecular evolutionary genetics analysis version 7.0 for bigger datasets. Mol. Biol. Evol..

[134] Ago H., Adachi H., Umena Y., Tashiro T., Kawakami K., Kamiya N., Tian L.R., Han G.Y., Kuang T.Y., Liu Z.Y., Wang F.J., Zou H.F., Enami I., Miyano M., Shen J.R. (2016). Novel features of eukaryotic Photosystem II revealed by its crystal structure analysis from a red alga. J. Biol. Chem..

[135] Yu L.J., Suga M., Wang-Otomo Z.Y., Shen J.R. (2018). Structure of photosynthetic LH1-RC supercomplex at 1.9 Å resolution. Nature.

[136] Gisriel C., Sarrou I., Ferlez B., Golbeck J.H., Redding K.E., Fromme R. (2017). Structure of a symmetric photosynthetic reaction center-photosystem. Science.

[137] Jordan, P., P. Fromme, H.T. Witt, O. Klukas, W. Saenger, and N. Krauss, Three-dimensional structure of cyanobacterial Photosystem I at 2.5 Å resolution. Nature, 2001. 411: 909–17. DOI:10.1038/35082000. 11418848

[138] Toporik H., Li J., Williams D., Chiu P.L., Mazor Y. (2019). The structure of the stress-induced photosystem I-IsiA antenna supercomplex. Nat. Struct. Mol. Biol..

[139] Jia Y.T., Dewey G., Shindyalov I.N., Bourne P.E. (2004). A new scoring function and associated statistical significance for structure alignment by CE. J. Comput. Biol..

[140] Kumar S., Stecher G., Suleski M., Hedges S.B. (2017). TimeTree: a resource for timelines, timetrees, and divergence times. Mol. Biol. Evol..

[141] Shih P.M., Ward L.M., Fischer W.W. (2017). Evolution of the 3-hydroxypropionate bicycle and recent transfer of anoxygenic photosynthesis into the Chloroflexi. Proc. Natl. Acad. Sci. U. S. A..

