# Supplementary material for: Time-resolved comparative molecular evolution of oxygenic photosynthesis
Source: Biochim Biophys Acta Bioenerg. 2021 Jun 1;1862(6):148400. doi: 10.1016/j.bbabio.2021.148400 (PMC8047818; doi:10.1016/j.bbabio.2021.148400)
Supplement: Supplementary file 2 — Supplementary figures and text [file mmc2.docx]

**Time-resolved comparative molecular evolution of oxygenic photosynthesis**

Thomas Oliver^1^, Patricia Sánchez-Baracaldo^2^, Anthony W. Larkum^3^, A. William Rutherford^1^, Tanai Cardona^1^*

^1^Department of Life Sciences, Imperial College London, London, UK

^2^School of Geographical Sciences, University of Bristol, Bristol, UK

^3^University of Technology Sydney, Ultimo NSW, Australia

*Correspondence to: t.cardona@imperial.ac.uk

**Content**

Suppementary Figure S1 Page 2

Suppementary Figure S2 Page 4

Suppementary Figure S3 Page 5

Suppementary Figure S4 Page 6

Suppementary Figure S5 Page 7

Suppementary Figure S6 Page 8

Suppementary Figure S7 Page 10

Suppementary Figure S8 Page 13

Suppementary Figure S9 Page 14

Suppementary Figure S10 Page 15

Suppementary Figure S11 Page 16

Suppementary Figure S12 Page 17

Supplementary Text S1 Page 18

Supplementary Text S2 Page 20

Supplementary Text S3 Page 26

References Page 31


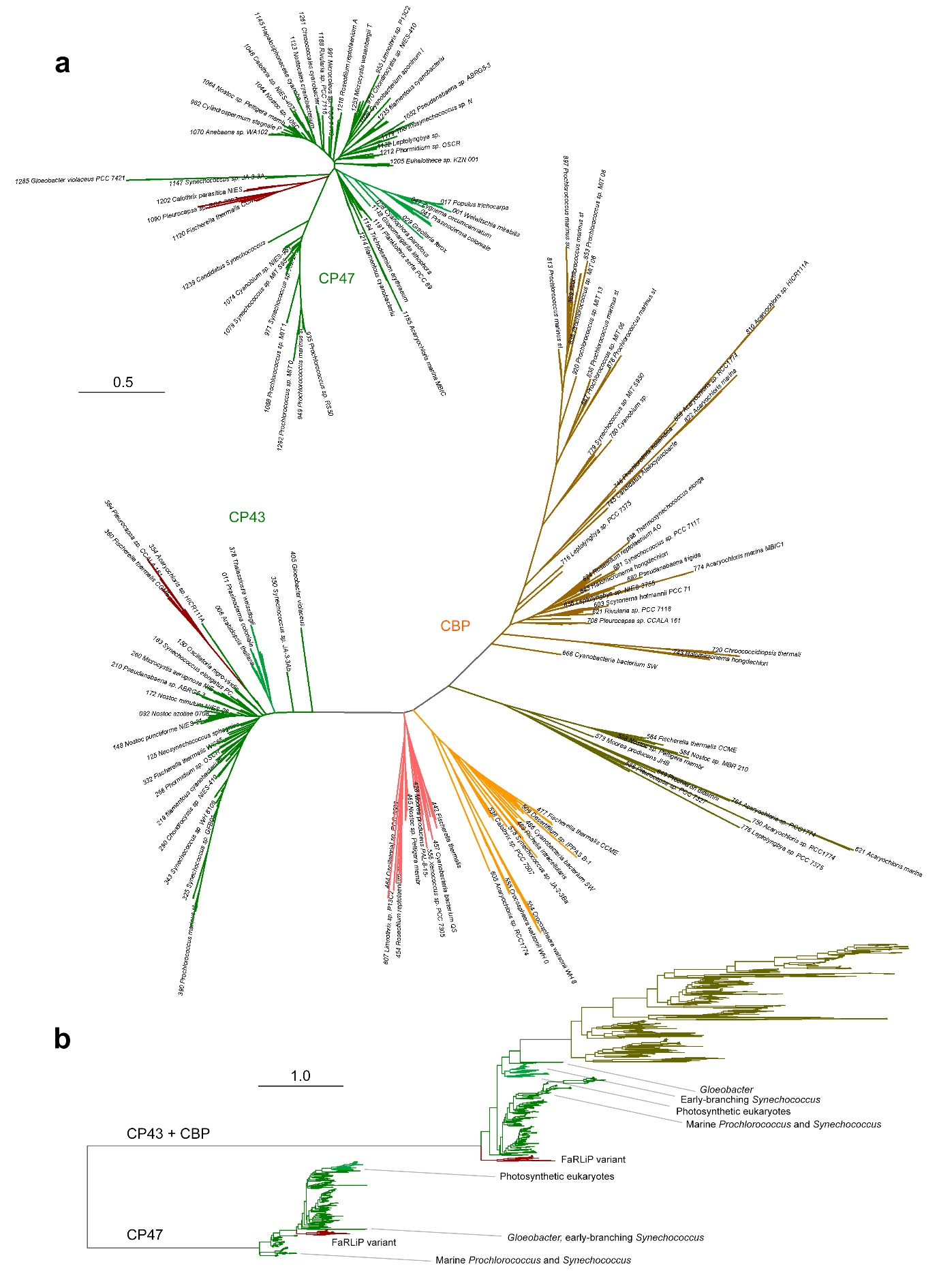


**Supplementary Figure S1.** Unrooted ML tree of CP43/CBP and CP47 subunits. **a** Unrooted trees of CP47 (top) and CP43/CBP (bottom). CP43 and CP47 are shown in green with the FaRLiP variants coloured red. CBP are coloured pink, orange and brown as shown in Figure 1 in the main text. **b** A ML tree using an alignment that included CP43 (green)/CBP (brown) and CP47 sequences. The generated tree highlights the long distance between CP47 and CP43/CBP. However, the faster evolving CBP sequences in combination with the long branches that separate CP43 and CP47 produces a phylogeny with strong long-branch attraction artefacts and an inverted CP43 topology regardless of model selection.


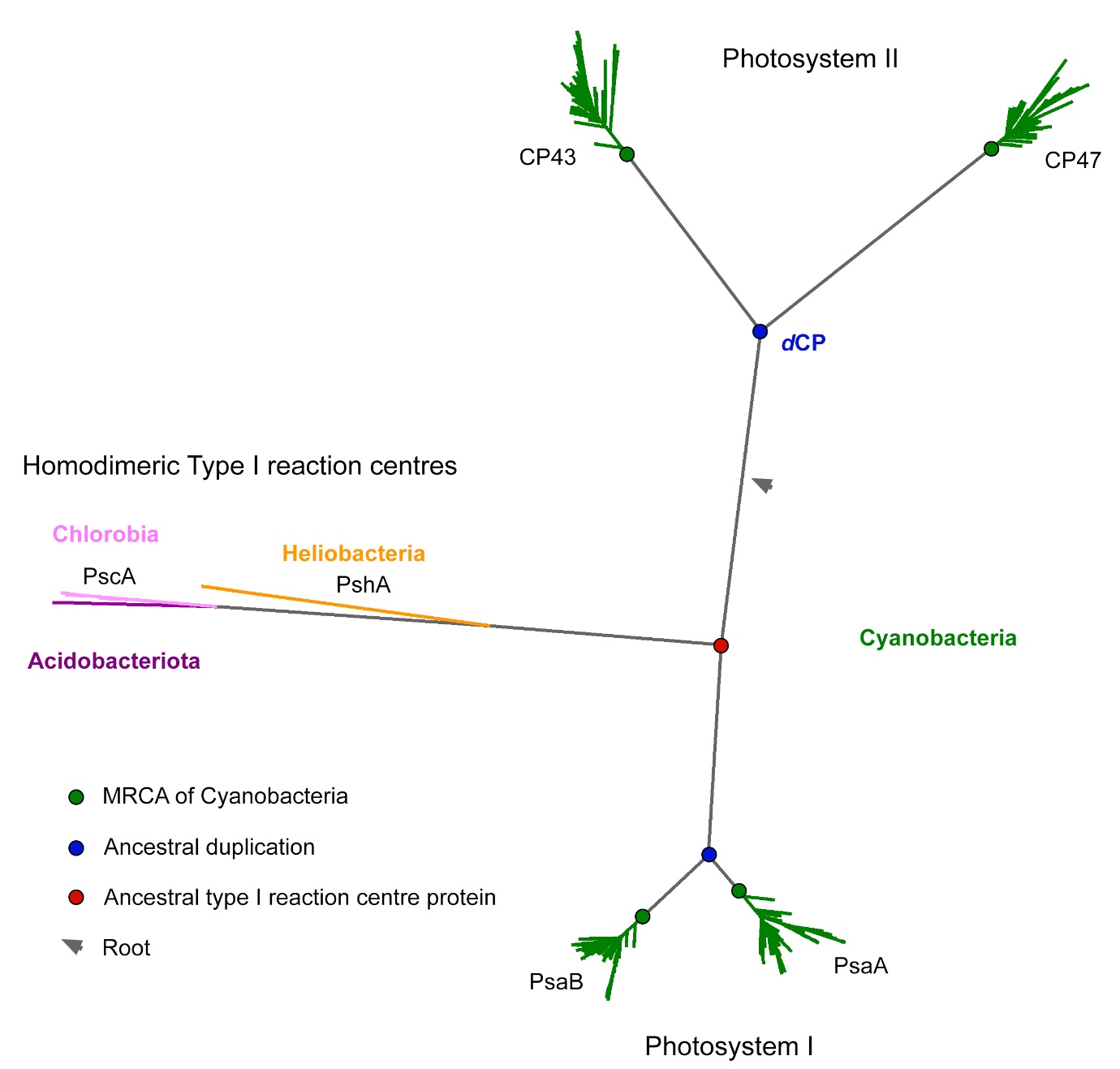


**Supplementary Figure S2.** ML tree of type I reaction centre proteins including CP43 and CP47. This tree highlights the long distance between the PSII antenna proteins and type I reaction centres. Type I reaction centre proteins are more likely to share a common ancestor to the exclusion of CP43 and CP47. This is supported by the observation that type I reaction centre proteins share greater sequence and structural identity (see also Supplementary Table S1). If that is the case, the root would therefore be placed at the longest branch (arrow). This placement is consistent with the phylogenetic relationships of reaction centre proteins showing that type I and type II reaction centres make separate monophyletic lineages [1, 2]. The blue dot represents the ancestral duplications that are specific to the evolution of oxygenic photosynthesis. Green dots represent the MRCA of Cyanobacteria, which inherited well-defined CP43/CP47; and PsaA and PsaB, the core subunit of photosystem I (PSI). Therefore, the duplications of the ancestral core photosystem proteins of oxygenic photosynthesis predate the MRCA of Cyanobacteria by a set amount of time.


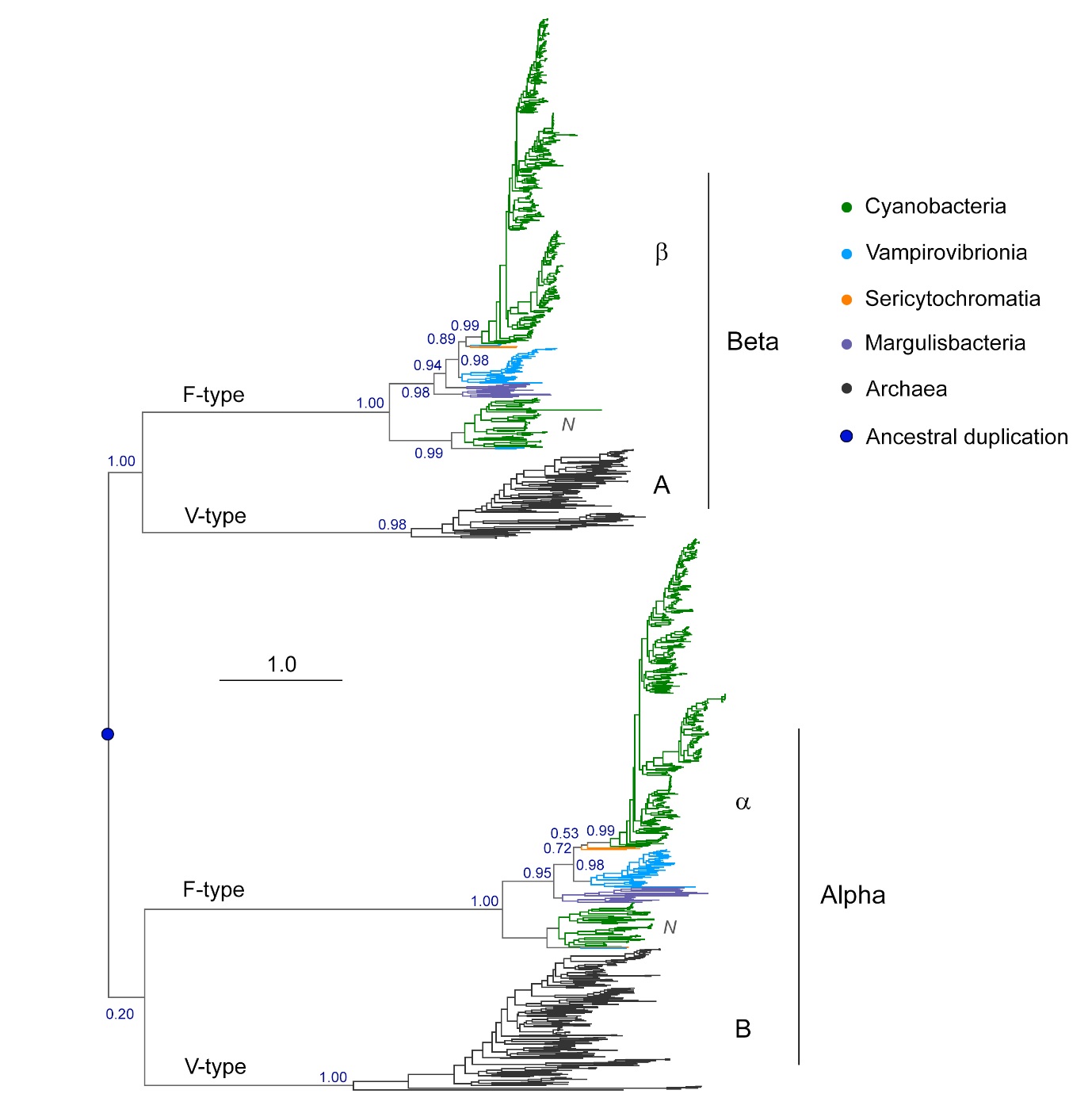


**Supplementary Figure S3.** ML tree of ATP synthase catalytic head subunits with emphasis on Cyanobacteria and their non-photosynthetic closest relatives. The evolution of ATP synthase is complex [3]. Overall, F-type is considered of bacterial origin and V-type of archaeal origin. Interdomain events of horizontal gene transfer are known and some strains of Margulisbacteria have been reported to have acquired V-type ATP synthase [4], for example. These are not included here. Alpha and Beta subunits share distant homology with other proteins containing P-loop NTPase domains. It is generally considered that the ancestral duplication that allowed the heterohexamerization of the catalytic head (F_1_, V_1_) is a very ancient event that occurred before the diversification of most forms of life and may even predate the LUCA. For simplicity, we refer in this work to the A and β subunit of ATP synthase together as Beta, and to B and α subunit as Alpha. *N* denotes Na^+^-translocating N-type ATPase of the bacterial F-type. Scale bar, number of substitutions per site.


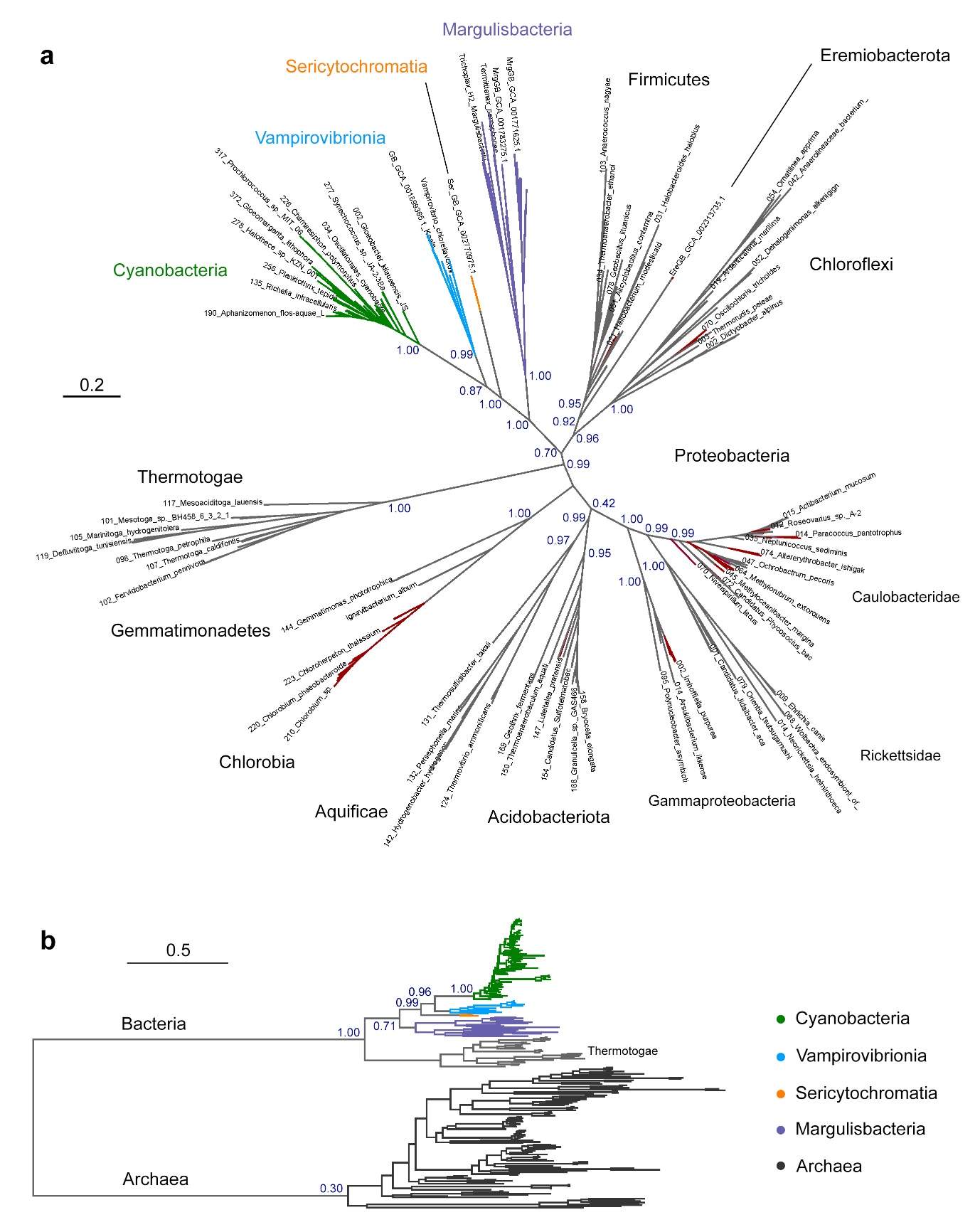


**Supplementary Figure S4**. ML trees of RNA polymerase subunit β (RpoB). **a** A tree of bacterial RpoB with emphasis on clades known to contain phototrophic representatives, Cyanobacteria and their closest non-photosynthetic relatives. Red branches denote phototrophic strains or clades. **b** A ML tree of selected bacterial RpoB and a diverse range of archaeal homologous sequences. Scale bars, number of substitutions per site.


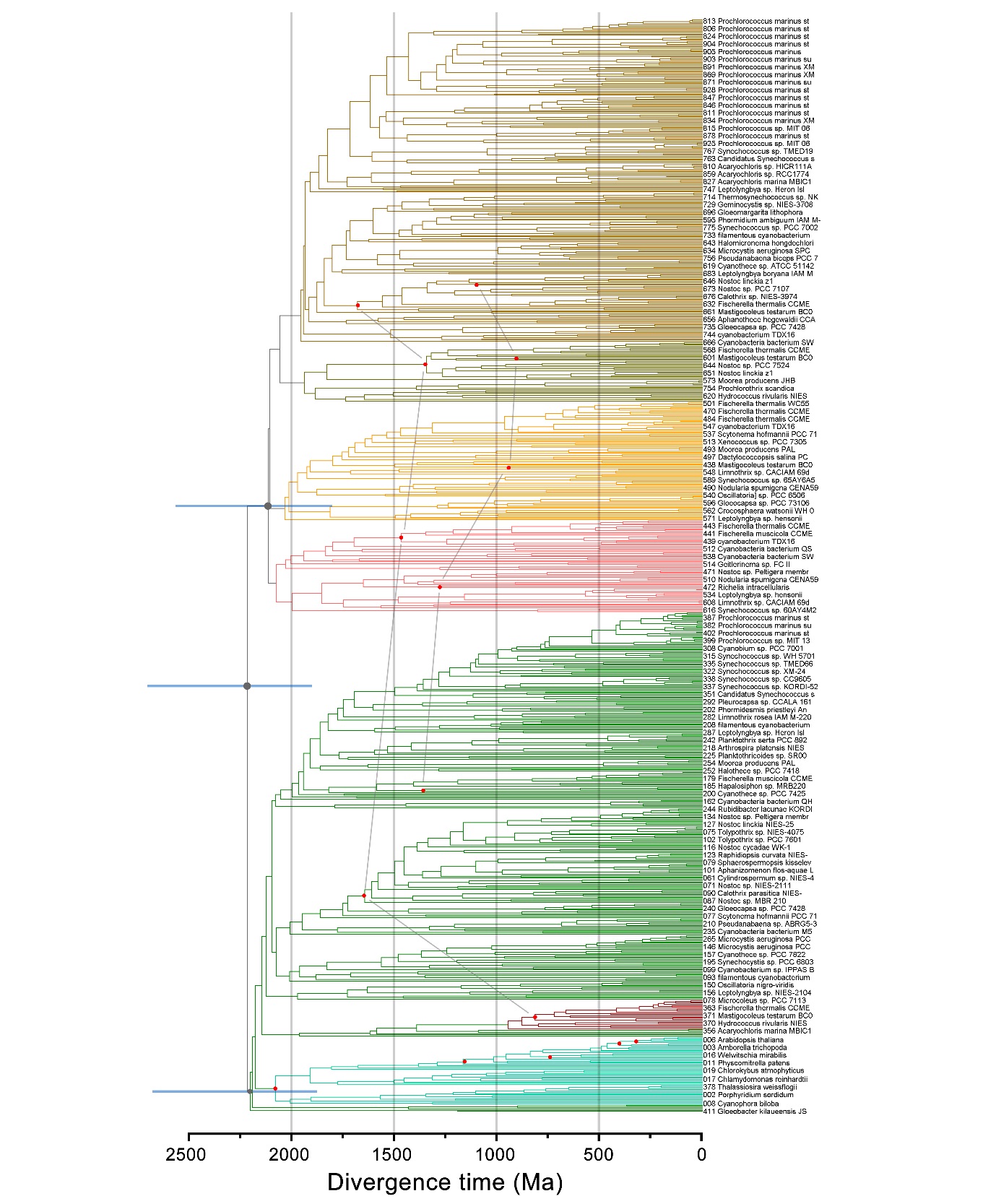


**Supplementary Figure S5.** Bayesian relaxed molecular clock of CP43 and CBP. This clock was calculated using a log normal autocorrelated molecular clock with a CAT+Γ non-parametric model of amino acid substitutions. The blue bars represent 95% confidence intervals at the deepest nodes. Red dots mark calibrated nodes and those connected by grey lines represent cross-calibrations across paralogues. The variation in the mean ages of the calibrated nodes is the result of the very broad constraints used in combination with the inherent variation of the rates of evolution within clades. The tree is colour-coded as seen in Figure 1 and Supplementary Figure 1a.

**
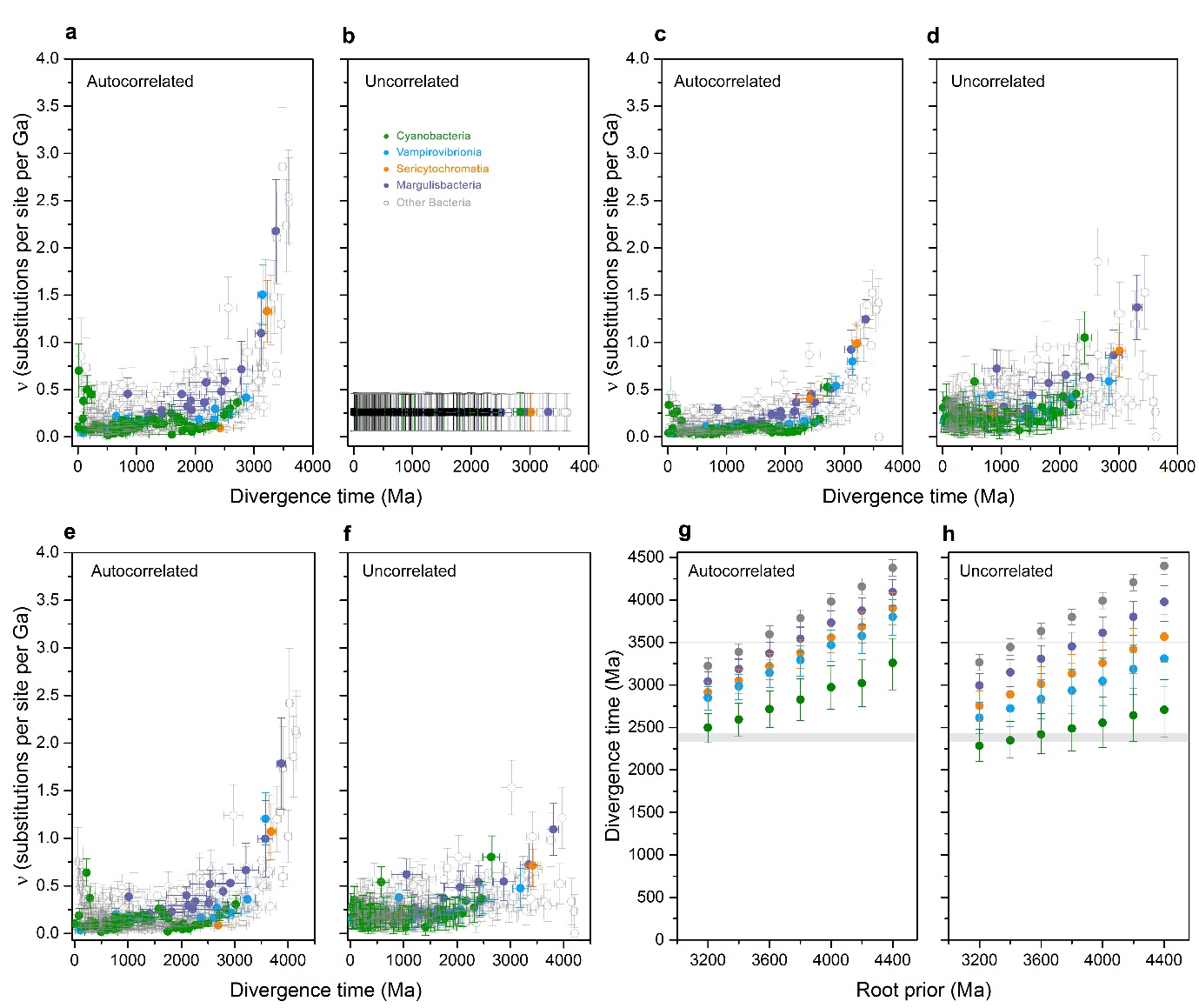
**

**Supplementary Figure S6.** Comparison of the rates of evolution determined using an autocorrelated and uncorrelated model of rate change for the bacterial RpoB sequence dataset. Conceptually, autocorrelated models assume that rates of evolution can vary across clades, but closely related clades are more likely to evolve at similar rates. Uncorrelated models assume that each branch of the tree can evolve at a rate that is independent from any other [5]. **a** Rates at internal nodes (instant rates) as a function of divergence time computed with a log normal autocorrelated relaxed clock. The root age was fixed at 3.6 Ga using a gamma root prior with a narrow standard deviation of 0.05 Ga. **b** Rates at internal nodes (instant rates) computed with an uncorrelated gamma model and a root prior of 3.6 ± 0.05 Ga. This model assumes that the rate assigned at every internal node of the tree is equal to the total average rate. **c** Average rate of evolution across internal branches of the same tree used to extract rates in panel **a**. The root has no length and therefore its rate is 0. **d** Average rate of evolution across internal branches of the same tree used to extract rates in panel **b**. A similar trend of decreasing rates through the Archean is observed, but a greater scatter is seen in comparison with an autocorrelated model. **e** Instant rates from an autocorrelated relaxed clock identical to **a** but using a root prior of 4.2 ± 0.05 Ga. **f** Average internal branch rates from an uncorrelated clock identical to **d**, but using a root prior of 4.2 ± 0.05 Ga. Panels **c** to **f** show that high rates of evolution are required during the Archean regardless of when exactly the domain Bacteria originated. The younger the estimated age of Bacteria, the faster the rates required at the initial stages of diversification. Panels **g** and **h** compare the estimated divergence times of the branching events leading to Thermotogae (grey), Margulisbacteria (violet), Sericytochromatia (orange), Vampirovibrionia (blue), and the MRCA of Cyanobacteria (green) for the two models using identical calibrations, but varying the root prior from 3.2 to 4.4 ± 0.05 Ga. The estimated ages are dependent on the age of the root, however, the span of time between ancestral nodes remains somewhat constant. There is a greater spread of dates generated with the uncorrelated gamma model. We attribute this difference to the assumption that all internal nodes evolve at the same rate, which is not necessarily reflective of true evolutionary processes. Error bars on the nodes represent standard error on mean values.


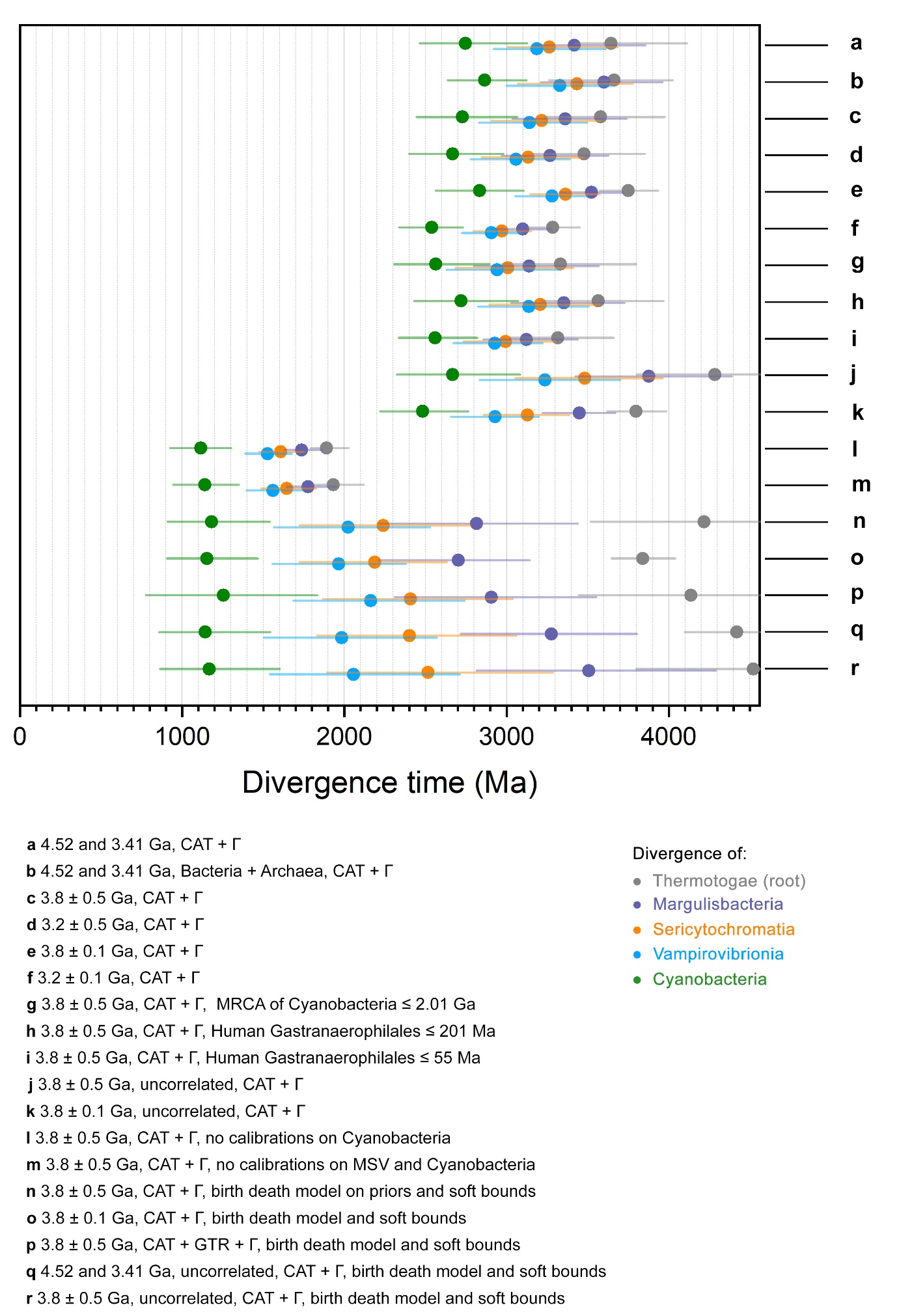


**Supplementary Figure S7.** Molecular clock **s**ensitivity analysis. Each row represents an independently performed molecular clock using various models as listed from **a** to **p** on the RpoB dataset. Each coloured dot represents the estimated divergence time for each respective node as indicated in the figure. Error bars represent 95% confidence intervals. Model **a** was calculated using a calibration on the root with a maximum of 4.52 Ga and a minimum of 3.41 Ga and applying a log normal autocorrelated clock with CAT+Γ. Model **b** was calculated under similar parameters as **a** but used the dataset that included sequences from Archaea. All other models used the sequence dataset that included only bacterial sequences. Model **c**, instead of a calibration on the root, used a gamma root prior with average of 3.8 Ga and a standard deviation (s.d.) of 0.5 Ga. Models **a** to **c** generated largely consistent results indicating that the root constraints are sufficiently broad to allow divergence times to converge towards similar ages.

Models **c** to **e** varied the gamma root prior and its s.d. while maintaining all calibrations and settings intact, to understand how the rates of evolution and ages would vary in connection to a younger or an older root. In model **c**, the prior was set to 3.8 ± 0.5 Ga, which is a relatively flexible constraint. This produced a posterior mean age on the root of 3.57 Ga (95% CI: 3.23 – 3.97 Ga). In model **d**, the prior average was changed to 3.2 Ga, while maintaining the broad s.d. of 0.5 Ga. This produced a posterior age of 3.47 Ga (95% CI: 3.16 – 3.85 Ga). Thus, when a flexible constraint on the root is used, the estimated mean age on the root converges towards 3.5-3.6 Ga, as in model **a** or **b**. Now, if the s.d. is made more restrictive (0.1 Ga), then the posterior age would be fixed within the set range. This allowed us to simulate other evolutionary scenarios. For example, model **e**, with a root prior of 3.2 ± 0.1 Ga, generated a posterior age of 3.28 Ga (95% CI: 3.13 – 3.45 Ga). Model **f** with a root prior of 3.8 ± 0.1 Ga, generated a posterior age of 3.75 Ga (95% CI: 3.57 – 3.93 Ga). It is unclear when exactly the domain Bacteria started to diversify. Therefore, model **e** would assume that Bacteria started to radiate late relative to **f**. We found that the span of time between the MRCA of Cyanobacteria and the root was found to be in the range of 0.75 for the most restrictive and young prior (**e**) to 0.91 Ga for the most restrictive and old prior (**f**), while the span of time between Cyanobacteria and Vampirovibrionia varied from 0.36 Ga (**e**) to 0.44 Ga (**f**). The latter being the same as in model **a** and **b** (see Figure 5 and 6a).

Model **g** varied instead a calibration point. In this case, the MRCA of Cyanobacteria was restricted with a maximum age of 2.01 Ga with no minimum age; and did not include the other two cyanobacterial calibrations described in Supplementary Text S3 (node 17 and 21) below. However, the estimated mean age for this node was 2.56 Ga (95% CI: 2.30 – 2.89 Ga). This change in calibration resulted in ages that were overall about 0.2 – 0.3 Ga younger for the deepest nodes relative to our benchmarking models, which did not include a maximum age for the MRCA of Cyanobacteria (e.g. **a**, **c**). This effect is a consequence of overall faster calculated rates. The span of time between Cyanobacteria and Vampirovibrionia was found to be 0.37 Ga and that between the root node and Cyanobacteria was found to be 0.76 Ga. A similar effect was observed when Vampirovibrionia are restricted to faster evolutionary rates instead (**h** and **i**), but maintaining the cyanobacterial calibrations (node 17 and 21). For example, in model **i**, the span of time between Vampirovibrionia and Cyanobacteria was calculated to be 0.36 Ga and between the root node and Cyanobacteria 0.75 Ga. The effect was less strong for model **h**, but followed a similar trend.

Uncorrelated gamma models (**j** and **k**) resulted in spread-out divergence time estimates (see also the preceding figure). If it is considered that this model is a better representation of the changes in the rates of evolution as a function of time, then this would imply a less explosive diversification of bacterial clades in contrast to what has been suggested in other independent analyses [6, 7]. Removal of all calibrations on Cyanobacteria and MSV resulted in collapsed age estimates (**l** and **m**).

Super-relaxed clock models that implemented soft bounds (**n** to **r**) resulted on a very large spread of ages that appear unrealistic, regardless of whether an autocorrelated and uncorrelated rate model was used, but replicate the results in ref. [8] that used “soft bounds” in all calculations, although **n** to **r** did not include an archaeal outgroup. We attribute this effect to the non-parametric rate smoothing method employed, which attempts to minimize differences in substitution rates among lineages (birth and death, soft bounds) [5]. It should be noted that soft bounds were originally developed for datasets featuring largely homogenous rates of protein evolution, such as highly-conserved mitochondrial proteins in primates, showing variation in sequence identity of just below 2%, and were not anticipated to be used on molecular clocks that contained representatives from all domains of life [9].

**
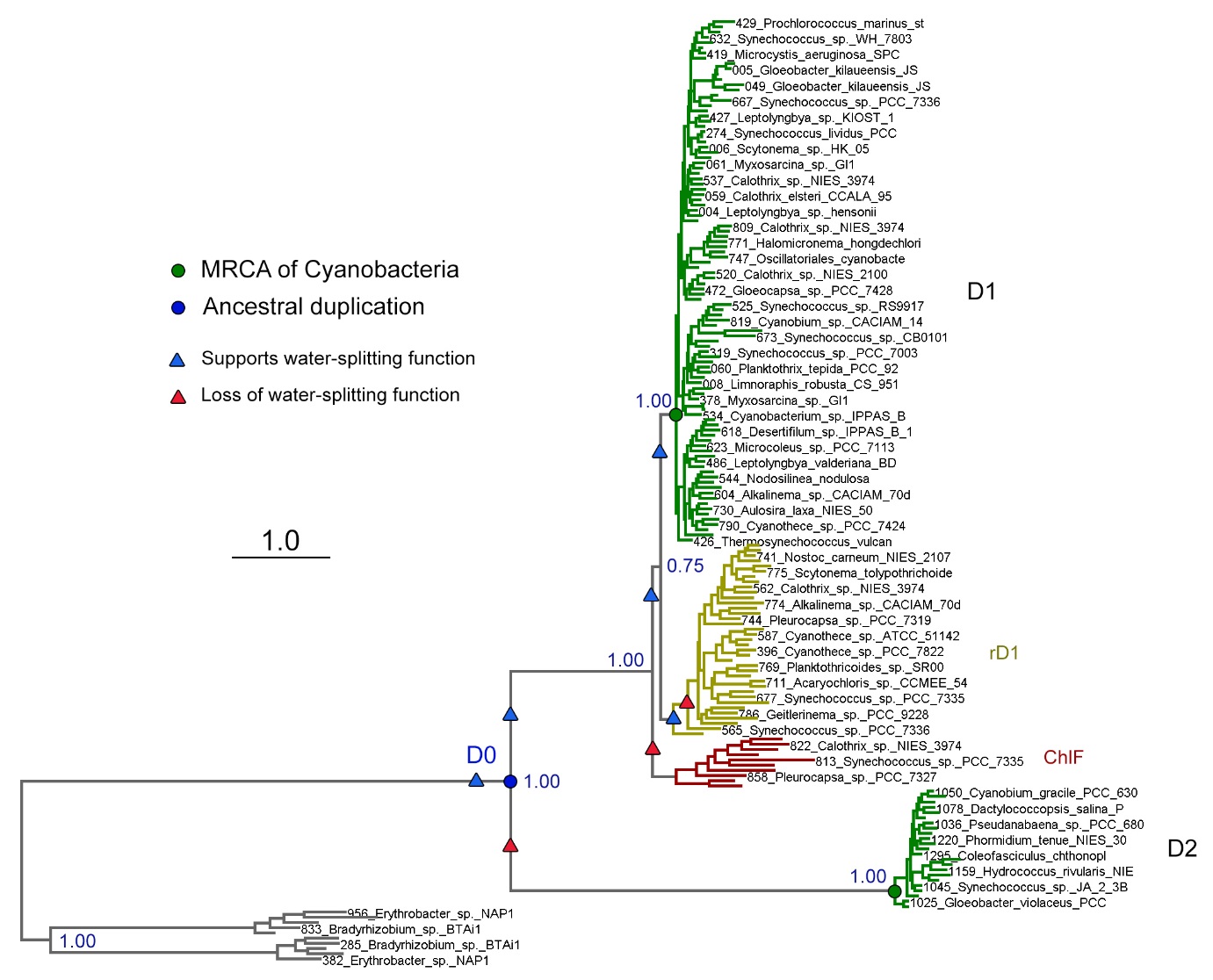
**

**Supplementary Figure S8.** ML tree of D1 and D2 sequences used for Ancestral Sequence Reconstruction. Green dots mark the standard D1 and D2 sequences inherited by the MRCA of Cyanobacteria [10]. Coloured triangles mark predicted capacity for supporting water oxidation as assessed by the presence or absence of ligands to the Mn_4_CaO_5_ cluster and overall similarity to D1. ChlF and rD1 are atypical D1 forms, characterized by lack of some, but not all, ligands to the Mn_4_CaO_5_ cluster as described in Cardona et al. [11] before. ChlF denotes the “super-rogue” D1 (Group 1 D1) or chlorophyll *f* synthase [12]. The clade denoted rD1 (“rogue” D1) is more widely distributed than ChlF. It has been proposed previously that this D1 has the role of “switching off” PSII in the dark [13] or under other conditions. We found that the ancestrally reconstructed sequence to all rD1 had a full set of ligands to the Mn_4_CaO_5_ cluster, with glutamate at position equivalent to 170 instead of aspartate. Furthermore, the earliest branching sequence in this clade was found to be that of *Halothece* sp. PCC 7418 (WP_015224801.1), which had a full set of ligands with E170 and reproduced the analysis in ref. [11], but this was not noted then. The ancestral sequence to ChlF was found to lack only a single ligand to the cluster, H332 and also featured E170. This is consistent with a scenario in which all atypical D1 sequences emerged from a D1 that was capable of water oxidation. All reconstructed sequences are available on request to the corresponding author.


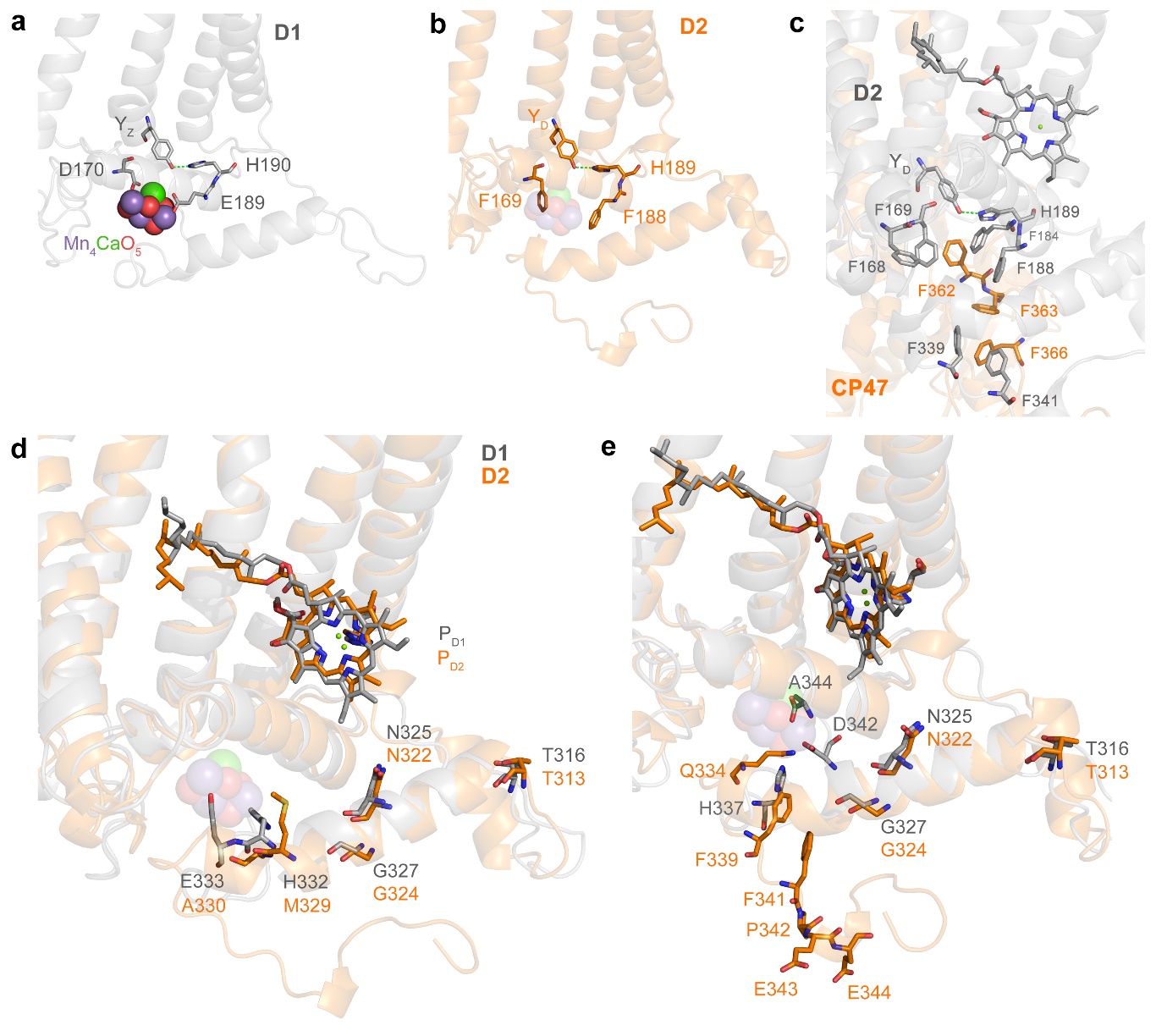
 **Supplementary Figure S9.** Comparison of the electron donor site of D1 and D2. **a** The Mn_4_CaO_5_ cluster and associated redox Y_Z_-H190 pair, as well as the inner ligands D170 and E189. **b** The electron donor site of D2 showing the redox active Y_D_-H189 pair and the phenylalanine residues that occupy positions homologous to D1-D170 and E189. The equivalent position of the Mn_4_CaO_5_ cluster is shown transparently from an overlap with D1. **c** Entire patch of phenylalanine residues blocking access to Y_D_-H189. **d** Overlap of D1 and D2. Residues occupying homologous positions are shown as sticks and assuming no indels after conserved residues D1-G327 and D2-G324. **e** Overlap of D1 and D2 focusing on the C-terminal residues. Assuming no indels, ligand D1-H337 would be equivalent to D2-Q334, D1-D342 to D2-F339, and D1-A344 to D2-F341. The codons of the *psbD* gene (D2) that encodes P342, E343, and E344 overlap with the Shine-Dalgarno ribosomal binding site of *psbC* (CP43).


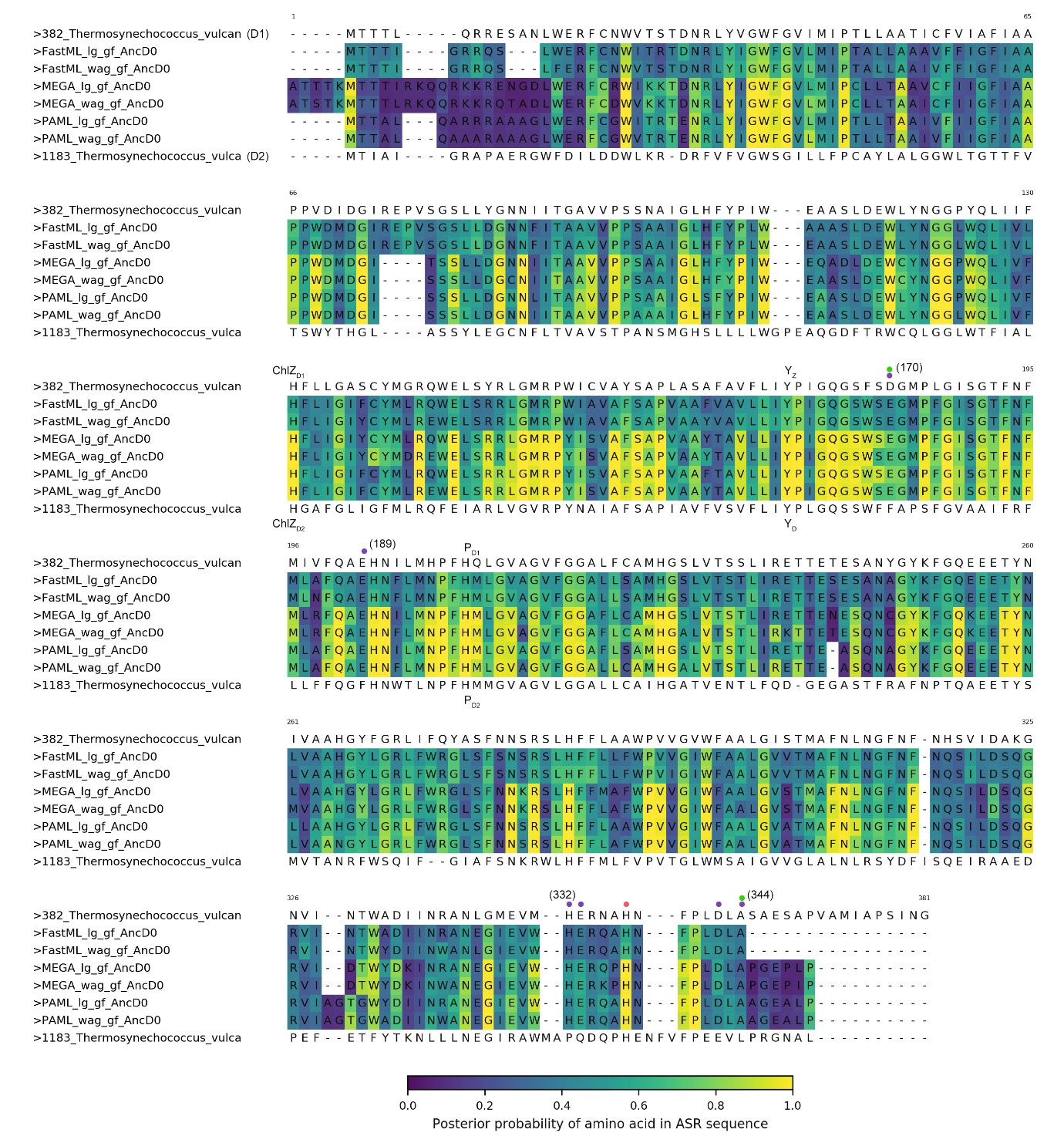


**Supplementary Figure S10.** Posterior probabilities (PP) of selected ancestrally reconstructed D0 sequences. The top and bottom sequences represent the D1 (PsbA1) and D2 subunits of *Thermosynechococcus vulcanus*. The colour at each site denotes the PP for the most likely residue as shown in the colour map at the bottom of the alignment. Ligands to the Mn_4_CaO_5_ cluster are highlighted with coloured dots: purple for Mn, green for Ca, and red for O. Y_Z_ and Y_D_ denote the redox active tyrosine residues; P_D1_ and P_D2_ denote the ligands to the chlorophylls equivalent to the “special pair”; ChlZ_D1_ and ChlZ_D2_ denote the ligands to these antenna chlorophylls.


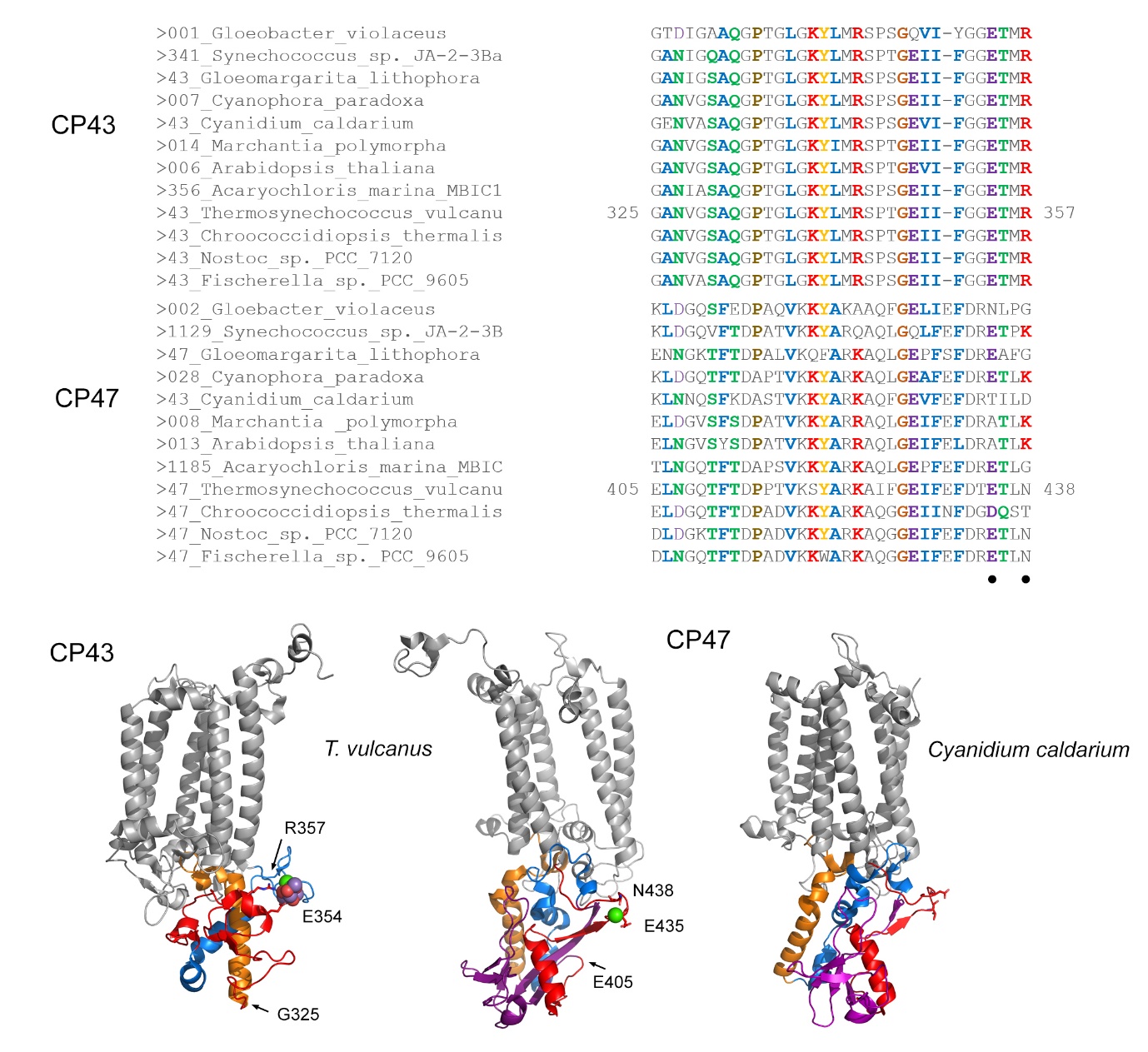


**Supplementary Figure S11.** Comparison of the region denoted as EF_3_ in the extrinsic domain of CP43 and CP47. The sequence alignments compare the protein fold highlighted in red ribbons on the structures below and as indicated with arrows. Sequence identity is detected indicating a swap of position in one subunit relative to the other. A Ca is bound in CP47 to E435 and N438, which are predicted to originate from homologous positions to the Mn_4_CaO_5_ cluster ligands CP43-E354 and R357. This Ca site is not observed in the 2.77 Å crystal structure of the red algae *Cyanidium caldarium* [14].

**
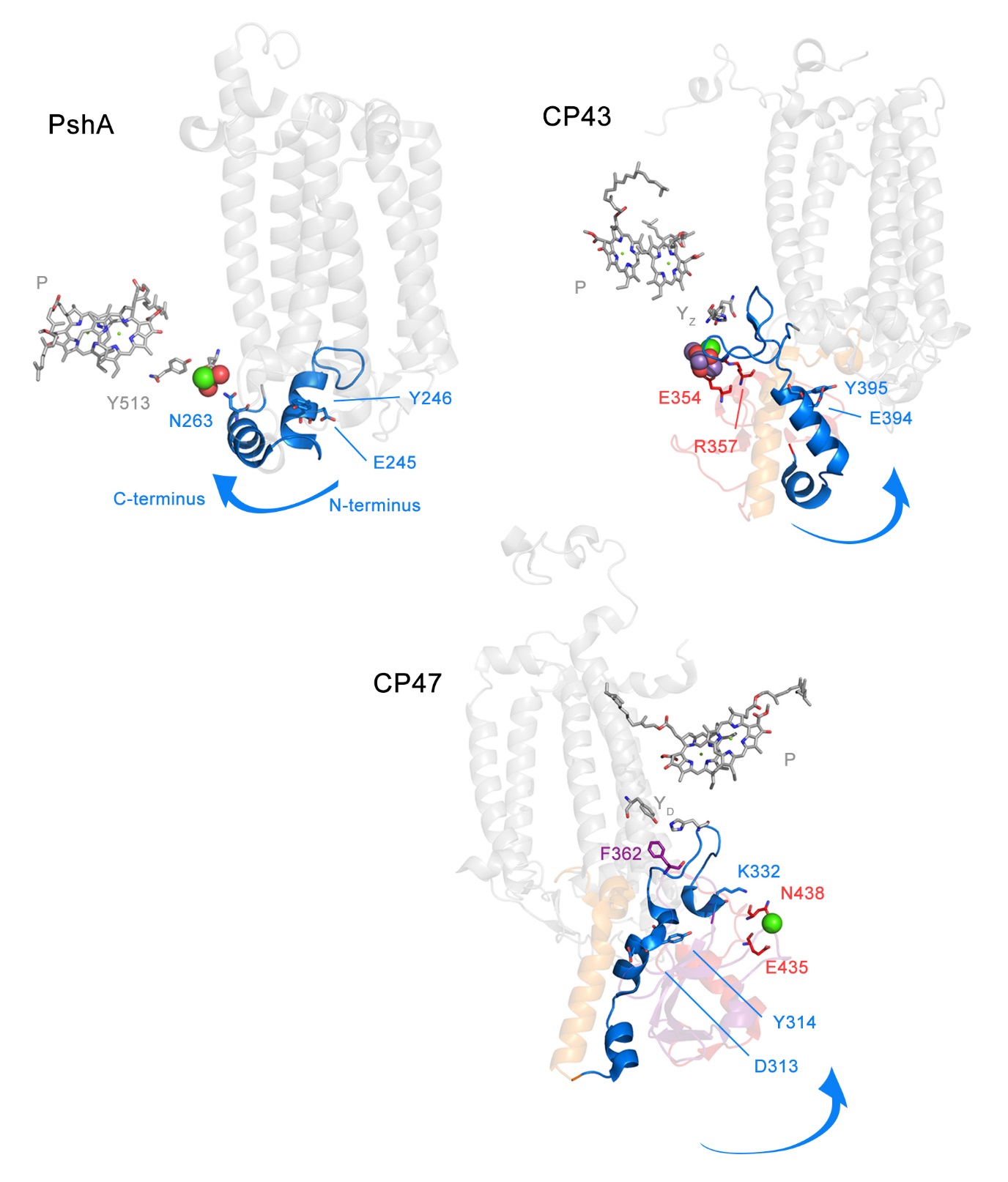
**

**Supplementary Figure S12.** Structural comparison of region denoted as EF_1_ in the extrinsic domain of heliobacterial PshA, CP43 and CP47. The fold is characterized by two small alpha helix and although there is little sequence conservation between PshA and the cyanobacterial sequences, the fold contain a potentially conserved and characteristic EY or DY pair. The blue arrow indicates the direction of the fold from N- to C-terminus.

**Supplementary Text S1. Rates of protein evolution in context**

Proteins display rates of amino acid substitutions that spans nearly six orders of magnitude (see image below). Take two identical sequences: if one of them evolves at a rate of 1 amino acid substitution per site per billion years and the other does not change, and assuming that all sites have a similar chance of mutating, it would take 1 Ga for each site to have changed once in the evolving sequence. This means that it would take under 1 Ga for these two hypothetical sequences two lose all sequence identity. Alternatively, if both sequence are evolving independently from each other at *that* same rate, it would take less than half that time, 500 Ma, for both sequences to lose all sequence identity, because both are accumulating change [15].


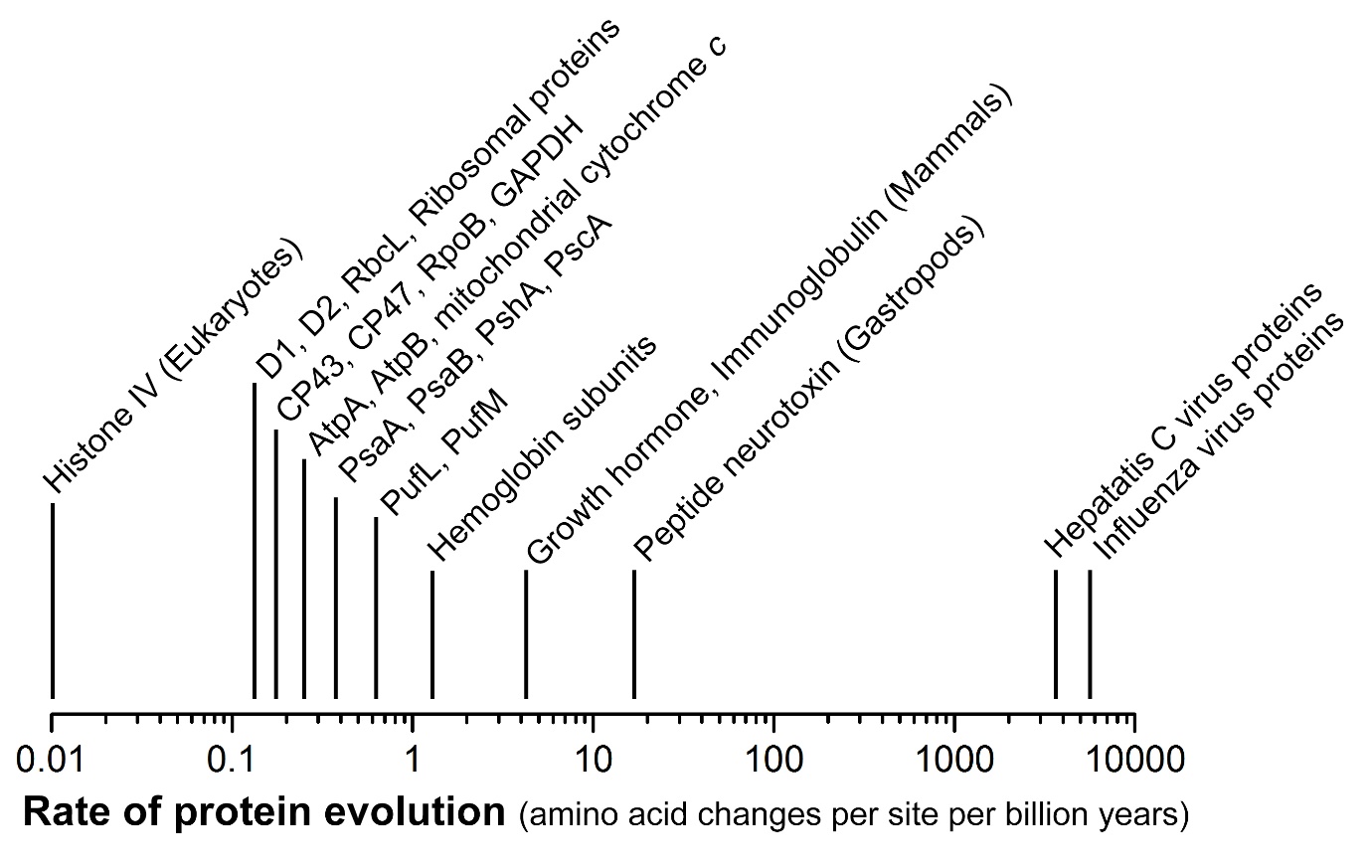


If two proteins are evolving at 10 amino acid substitutions per site per Ga, assuming the same hypothetical conditions in which all sites and all substitutions are equally probable, it would take less than 50 Ma for the two identical sequences to lose all trace of identity. On the other hand, if the two sequences were evolving at 0.1 amino acid substitutions per site per Ga, it would take 5 Ga for them to lose all sequence identity. That means that after a period of 2.5 Ga of evolution, these two slowly evolving sequences would still retain about 50% sequence identity. Therefore, most highly-conserved proteins across the tree of life, that are predicted to be very ancient, tracing back to the LUCA or the earliest forms of life, such as ATP synthase, RNA polymerase, or the ribosome; proteins that have retained substantial sequence identity over a multibillion year history, evolve at rates well below 1 amino substitution per site per Ga. In this way, for example, if we compare the protein sequence of RNA polymerase subunit B (RpoB) between two very distantly related bacteria such as *Thermotoga* (Thermotogae) and *Thermosynechococcus* (Cyanobacteria), the level of sequence identity is about 40% including gap and insertions. If we consider that the phyla containing these two clades diverged over 2.5 Ga ago, it can be expected that these sequences have been evolving at rates on average somewhat above 0.1 amino acid changes per site per Ga, but certainly well below 1.

In contrast, fast evolving proteins, like those found in viruses, that accumulate change in just a few months or years, display rates of evolution of several thousand changes per site per billion years. At the other extreme, one of the slowest evolving protein known is histone subunit H4, a protein of about 100 residues that has barely changed at all within the past billion years, showing just 2 amino acid changes between *Arabidopsis* and *Homo sapiens*, for example.

Molecular clocks will use a protein sequence alignment and some known time constraints (calibrations) to estimate and extrapolate the rates of protein evolution in amino acid changes per site per unit of time, across a phylogenetic tree, much in the same way that we have considered in the preceding paragraphs. Thus, knowing the *distance* between two sequences (number of amino acid changes per site), and the rate of protein evolution (the *speed* of sequence change), it is possible to estimate the divergence *time*. Modern molecular clocks with the implementation of Maximum Likelihood or Bayesian Inference methods can account for the uncertainty generated by sparse calibration points, and the fact that in reality different clades, different sequences, and different parts in a sequence evolve at different rates.

In the image above, the rates of evolution for D1, D2 , PufL and PufM were taken from ref. [10]. Those for PsaA, PsaB, PshA, PscA from ref. [16]. For CP43, CP47, AtpA, AtpB, RpoB and ribosomal proteins were taken from this study. For histone IV, GAPDH, cytochrome *c*, hemoglobin, growth hormone and immunoglobulin and other not shown here, see ref. [17]. For gastropod neurotoxins and viral proteins, see also [10] and references therein.

**Supplementary Text S2.** **Quantification of rates of evolution**

*Rationale*

Molecular clocks are conventionally used to estimate divergence times. In general terms, given: 1) a tree topology, which sets the relationship between taxa; 2) a sequence alignment, which sets the phylogenetic distance between taxa; and 3) some known events (calibrations), which set the rates of evolution, the molecular clock can then estimate divergence times. This means that if the tree topology and divergence times for two sets of protein sequences are the same, any differences in phylogenetic distances between these two should only reflect differences in the rate of evolution. Thus, assuming that CP43/CP47 and Alpha/Beta have mainly been inherited vertically in Cyanobacteria and photosynthetic eukaryotes, any difference in phylogenetic distance between the two is the result of differences in the rates of evolution. For example, the level of sequence identity between CP43 in *Cyanidioschizon* and *Arabidopsis* is 78%, and the level of sequence identity between Alpha in the same species is 69%. Given that these plastid-encoded subunits have mostly been inherited vertically since the MRCA of Archaeplastida, then one can argue that Alpha is evolving somewhat faster than CP43. This is because faster rates of protein evolution should lead to faster rates of change resulting in a faster decrease in the level of sequence identity (increase in phylogenetic distance).

Now, because CP43 and CP47 are paralogues, we can then use this approach to estimate the rates of evolution at the moment of duplication under a given number of specific scenarios. For example, assuming that the MRCA of Cyanobacteria occurred at 2.0 Ga, we can then model how the rates of evolution would change at the moment of duplication if this occurred at 2.5, 3.0, 3.5 Ga, or at any other time point of interest. Because these set of proteins have likely achieved mutational saturation at the largest distance, the rate of evolution at the point of duplication will be an underestimation (slower than it should be), given that saturation would hide substitution events. Similarly, keeping the time of duplication constant, we can then model how the rates of evolution would change across the tree if the MRCA of Cyanobacteria is assumed to have occurred at 2.0, 2.5, 3.0 Ga, or at any other time of interest.

Therefore, even though it is difficult to determine the absolute time of origin of Cyanobacteria, or of the early duplications of the core subunits of ATP synthase, it is possible to determine what rates of evolution are required to fulfil any particular scenario.

*Method*

To measure rates of evolution of CP43/CP47 and Alpha/Beta, as shown in Figure 4 a-d, a total of 19 sequences from photosynthetic eukaryotes and 4 cyanobacterial sequences per subunit were selected. A standardized tree topology was constructed from consensus evolutionary relationships as illustrated in the image below (panel **a**):

1. Relationships between land plants was taken from ref. [18].
2. It is well established that the divergence of red algae predates the MRCA of land plants [19-21].
3. Ponce-Toledo, Deschamps [22] recently suggested that *Gloeomargarita* is the closest living cyanobacterial relative to the plastid ancestor and predated the emergence of heterocystous Cyanobacteria, but see also [20].
4. The clade containing *Chroococcidiopsis thermalis* PCC 7203 is one of heterocystous Cyanobacteria’s closest non-heterocystous relatives [23, 24].
5. *Gloeobacter* spp. is the earliest branching and well-described genus of Cyanobacteria capable of oxygenic photosynthesis [24-28].

**
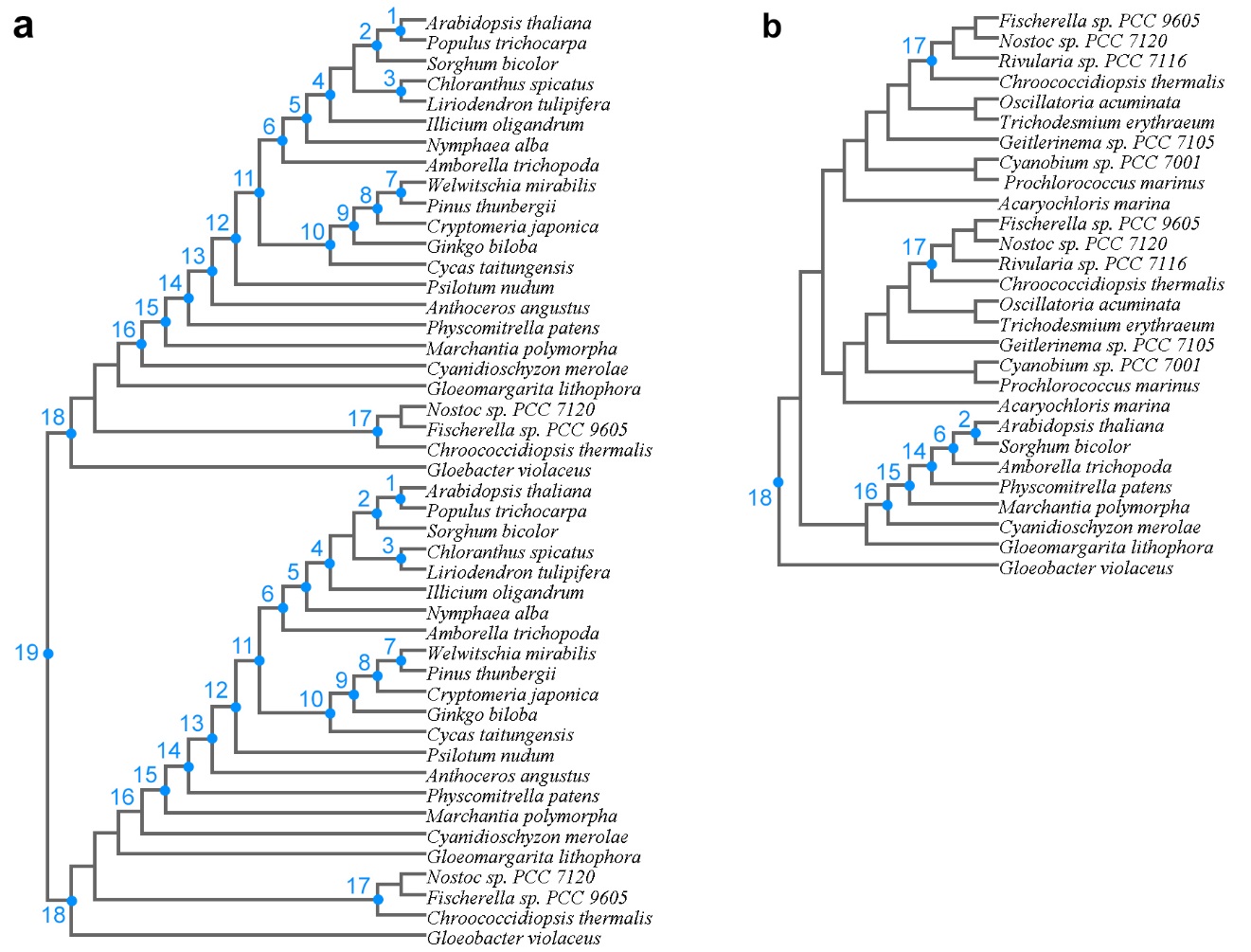
**

Calibration points were allocated as marked with numbered blue dots and listed in the table shown in page 27. Nodes 1 to 15 were applied following the justifications and best practice listed in ref. [18], but using a 515 Ma maximum agie calibration for land plants as suggested in ref. [29]. Node 16 represents here the MRCA of red and green lineages of photosynthetic eukaryotes. The minimum age was set to 1.0 Ga based on the *Bangiomorpha* fossil as described above [21, 30]. The maximum age was set to 1.8 Ga, which is similar to the oldest ages reported in recent molecular clock analyses for the MRCA of photosynthetic eukaryotes and it is also similar to the age of the earliest plausible fossilised unicellular eukaryotes [31, 32]. That said, compelling fossil evidence for eukaryotes has been presented as far back as 2.1 Ga [33]. Node 17 marks the divergence of heterocystous Cyanobacteria and it was given a minimum age of 0.72 Ga based on the recently described fossils of filaments bearing clear heterocysts from the Tonian period [34]. A maximum age of 1.65 Ga was given to this node, based on the recent report of heterocystous cyanobacteria from the Gaoyuzhuang Formation [35]. Although, it is unclear if reported akinetes that are older than 1.65 Ga are truly that [36], we considered that the 0.72-1.65 Ma range for the origin of heterocystous Cyanobacteria was a reasonably broad constraint for the purpose of this experiment, but see below.

Node 18 denotes the MRCA of Cyanobacteria. The age for this node is highly debated ranging from before to after the GOE. Node 19 represents the duplication events leading to CP43 and CP47, and to Alpha and Beta. To calculate the rates of evolution under different scenarios, node 18 and node 19 were varied. Firstly, a molecular clock was run using a scenario that assumed that the MRCA of Cyanobacteria postdated the GOE. To do this, node 18 was set to be between 1.6 and 1.8 Ga, which emulates results reported in recent studies [8, 37]. This was compared to a scenario that assumed that the MRCA of Cyanobacteria antedated the GOE, and thus node 18 was set to be between 2.6 and 2.8 Ga, which simulates other evolutionary scenarios [20, 38]. In both cases, the duplication event (node 19) was set to be 3.5 Ga old, or changed as stated in the main text, by assigning a gamma prior at the desired time fixed with a narrow standard deviation of 0.05 Ga. In a separate experiment, the age of the duplication was varied while maintaining node 18 restricted to between 1.6 and 1.8 Ga, while node 19, the duplication point, was set with a gamma prior with an average varied from 0.8 to 4.2 and with a narrow standard deviation of 0.05 Ga.

The period of time between the duplication event (node 19), which led to the divergence of CP43 and CP47, and the MRCA of Cyanobacteria (node 18), we define as ΔT. ΔT is calculated as the subtraction of the mean age of node 19 and node 18. For PSII, we used node 18 from the CP43 subunit and for ATP synthase we used node 18 from the Alpha subunit. In consequence, varying the age of the duplication from 0.8 to 4.2 Ga allows changes in the rate of evolution to be simulated with varying ΔT, ranging from 0.2 to 2.5 Ga. That is to say, it allows the rate of evolution at the point of duplication to be calculated as if it had occurred at any point in time before the MRCA of Cyanobacteria.

As an additional comparison, we calculated rates of evolution for cyanobacterial FtsH subunits, which AAA ATPase domain is structurally similar to the catalytic head of ATP synthase [39]. Cyanobacterial FtsH are involved in membrane protein quality control, with some isoforms targeting specifically PSII subunits. It features a late duplication event leading to cyanobacterial FtsH1 and FtsH2 subunits (using the nomenclature of Shao et al. [40]). It was shown previously that the duplication leading to FtsH1 and FtsH2 occurred after the divergence of *Gloeobacter* spp. Genes encoding plastid FtsH subunits are encoded in the nuclear genome of photosynthetic eukaryotes. Because not all the selected species had fully sequenced nuclear genomes, only those with available FtsH sequences at the time of this study were used. The tree topology shown in panel **b** was used as template for the calculation of the rate of evolution and was based on that presented by Shao et al. [40]. It was concluded that the Cyanobacteria-inherited closest paralog to FtsH1 and FtsH2 in photosynthetic eukaryotes was also acquired before their initial duplication. Therefore, from all FtsH paralogs in photosynthetic eukaryote genomes, those with greater sequence identity to cyanobacterial FtsH1/2 were used. Because this duplication is specific to Cyanobacteria, a few additional strains were included in this tree following well-established topologies [20, 24]. Calibrations were placed as indicated in panel **b**, numbered blue dots. To test the change in the rate of evolution at the time of duplication in comparison with CP43/CP47, node 19 was set to 1.6-1.8 Ga or 2.6-2.8 Ga.

Rates were calculated with Phylobayes 3.3 using a log normal autocorrelated molecular clock model under the CAT+Γ non-parametric model of amino acid substitution and with a uniform distribution of equilibrium frequencies. Four discrete categories for the gamma distribution were used and four chains were executed in parallel until convergence. The instant rates of evolution, which are the rates at each internal node in the tree, were retrieved from the output files of Phylobayes without modification and expressed as amino acid substitutions per site per Ga [41, 42].

Finally, we conducted a large molecular clock using the combined 897 CP43 and CBP sequences, including 40 eukaryotic CP43 sequences, to test whether using a more complex phylogeny would result in rates of evolution substantially different to those calculated with the method described above. This dataset was also interesting because it includes several gene duplication events associated with the origin of CBP. Calibrations were assigned as illustrated in the following two images below (Part 1 and Part 2) that follow the phylogeny presented in Figure 1a.

**
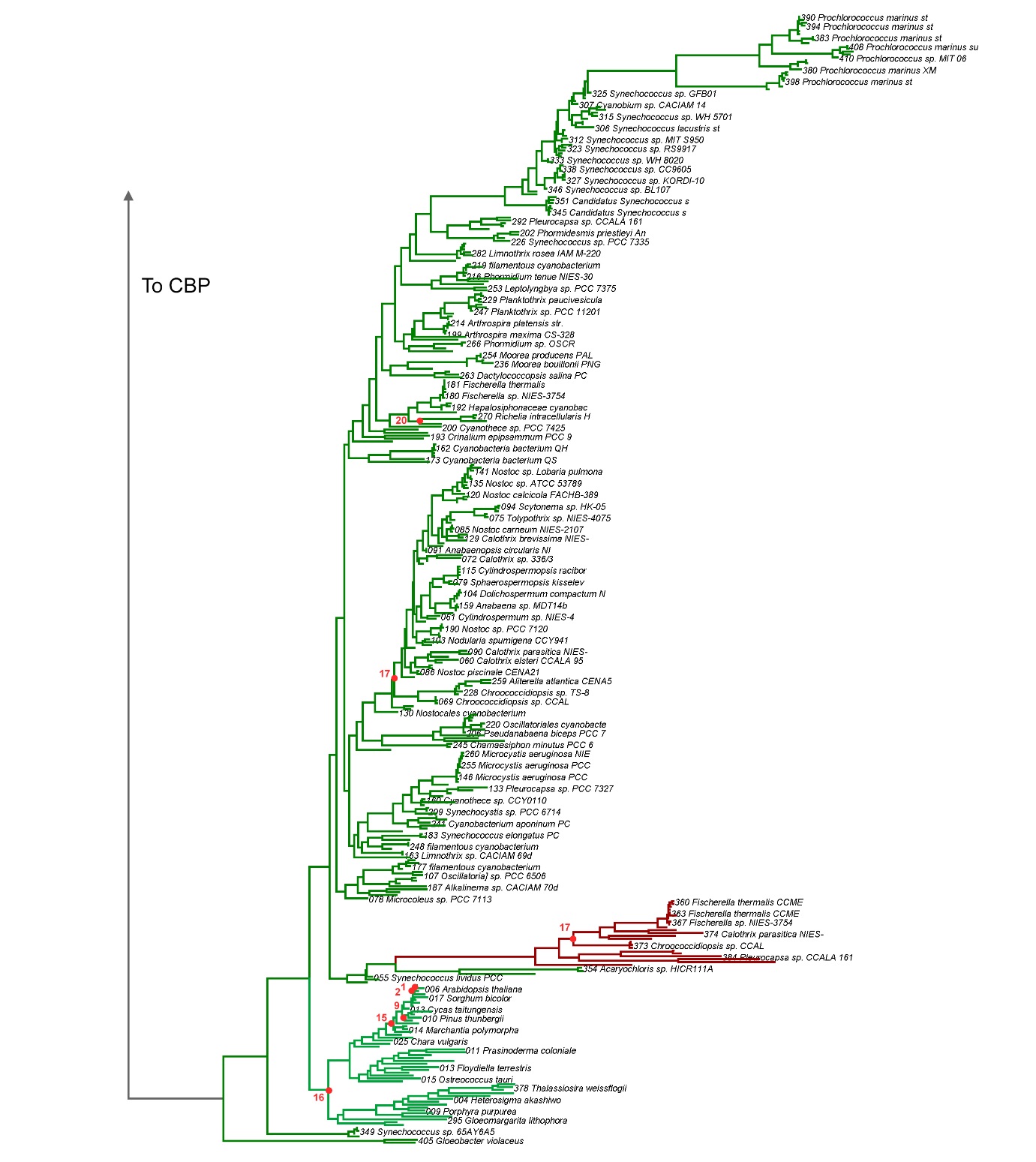
**

***Part 1.***

**
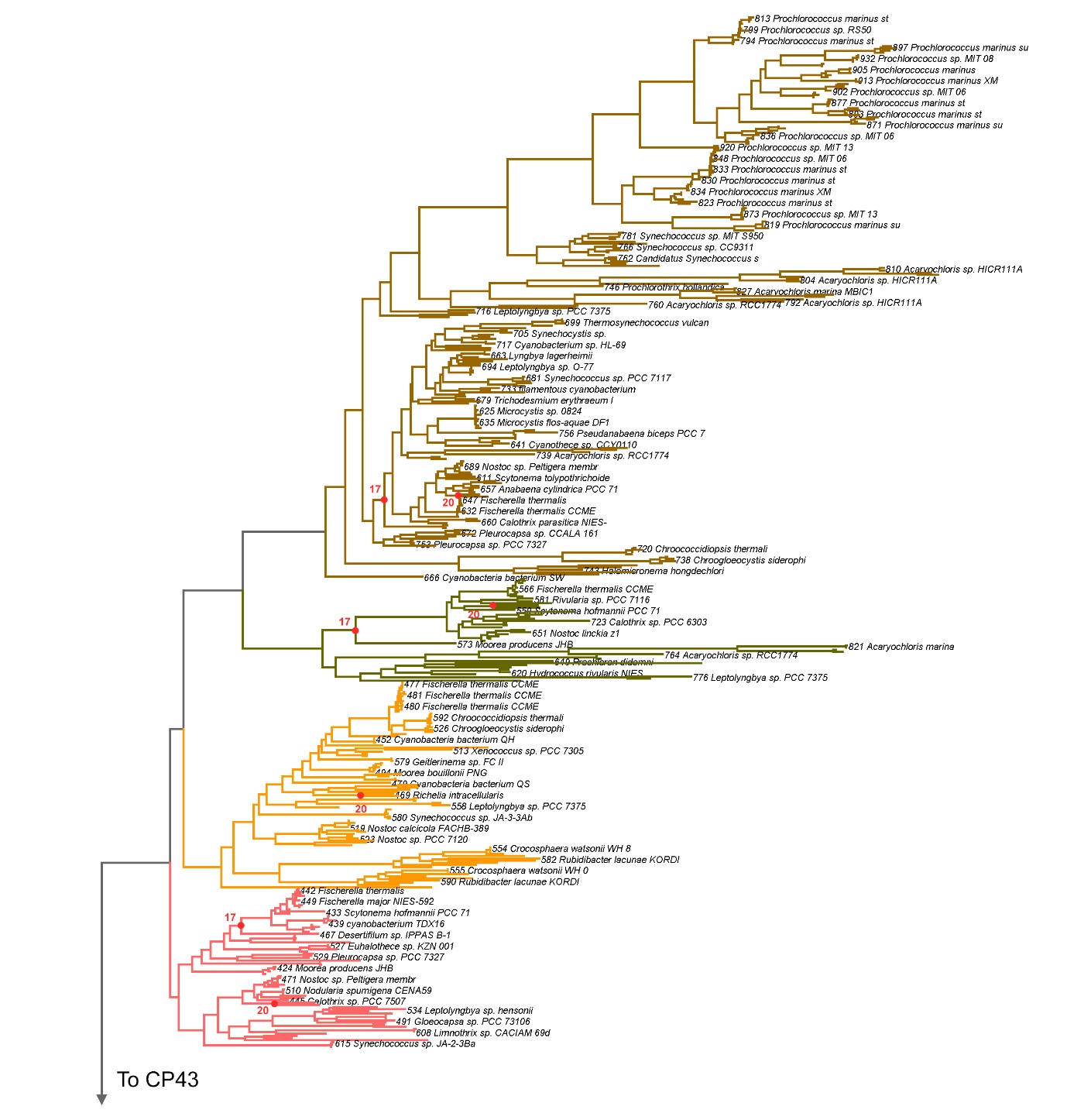
**

***Part 2.***

Cross-calibrations were used across paralogs constraining the origin of heterocystous Cyanobacteria. In this case, only the minimum constraint of 0.72 Ga was used with no maximum constraint to allow greater flexibility. Additional calibrations were assigned also across paralogs (point 20), this was considered as the node made by *Richelia intracellularis* and its closest sister sequence, as implemented in ref. [38]. This strain is a specific endosymbiont of a diatom and its divergence was set to be no older than the earliest discussed age for diatoms [43]. The root equivalent to the MRCA of Cyanobacteria (divergence of *Gloeobacter* in CP43) was not calibrated. The root of the tree was instead varied: first it was given a maximum age of 4.52 Ga as recently implemented and justified by Betts, et al. [8] as the earliest plausible time in which the planet was inhabitable after the moon forming impact [44], and no minimum age was used. A second tree was executed with no constraint on the root and no root prior. A third root was implemented constrained to be between 2.3 Ga (the GOE) and 3.2 Ga. The latter date represents the age of the cyanobacteria-like well-preserved microbial mats of the Berberton Greenstone Belt in South Africa and neighbouring Eswatini [45]. Rates were obtained using the autocorrelated CAT+Γ model as described above. Because these root constraints did not have a strong effect in the overall estimated rates, we carried out an additional control applying an uncorrelated gamma clock model [46] with a root constrained at 4.52 Ga and no minimum age.

Time constraints

| **Node label** | **Relationship** | **Max age (Ma)** | **Min age (Ma)** |
| --- | --- | --- | --- |
| 1 | *Arabidopsis* v *Populus* | 127 | 82 |
| 2 | *Arabidopsis* v *Sorghum* | 248 | 124 |
| 3 | *Chloranthus* v *Liriodendron* | 248 | 98 |
| 4 | *Arabidopsis* v *Illicium* | 248 | 124 |
| 5 | *Arabidopsis* v *Nymphaea* | 248 | 124 |
| 6 | *Arabidopsis* v *Amborella* | 248 | 124 |
| 7 | *Welwitschia* v *Pinus* | 309 | 121 |
| 8 | *Welwitschia* v *Cryptomeria* | 309 | 147 |
| 9 | *Welwitschia* v *Ginkgo* | 366 | 107 |
| 10 | *Welwitschia* v *Cycas* | 366 | 306 |
| 11 | *Arabidopsis* v *Cycas* | 366 | 306 |
| 12 | *Arabidopsis* v *Psilotun* | 454 | 388 |
| 13 | *Arabidopsis* v *Anthoceros* | 515 | 420 |
| 14 | *Arabidopsis* v *Physcomitrella* | 515 | 420 |
| 15 | *Arabidopsis* v *Marchantia* | 515 | 449 |
| 16 | *Arabidopsis* v *Cyanidioschyzon* | 1800 | 1000 |
| 17 | *Nostoc* v closest non-heterocystous strain | 1560 (or none) | 720 |
| 18 | *Arabidopsis* v *Gloeobacter* | Variable (or none) | Variable (or none) |
| 19 | CP43 v CP47 or AtpA v AtpB duplications | Variable | Variable |
| 20 | *Richelia intracelullaris* v closest neighbouring sequence | 250 | 100 |
| 21 | Chroococcales cyanobacteria | — | 2016 |
| 22 | *Termititenax* | 396 | 140 |
| 23 | *Candidatus* Ruthmannia eludens v GCA 002716725.1 | 660 | 542 |
| 24a | GB GCA 001899365.1 Koala v GB GCA 001917115.1 Homo | 201 (or 55) | — |
| 24b | GB GCA 001917115.1 *Homo* v GB GCA 000980455.1 Homo | 201 | — |
| 25 | GB GCA 001917115.1 *Homo* v *Vampirovibrio chlorellavorus* | 1800 | — |
| 26 | *Polynucleobacter necessarius* | 444 | — |
| 27 | *Bradyrhizobium* | 86 | — |
| 28 | Rickettsias | — | 1800 |
| 29 | *Wolbachia* and *Anaplasma* | 395 | — |
| 30 | Phototrophic Chlorobia | — | 1640 |
| 31 | Chromatiaceae | — | 1640 |

**Supplementary Text S3.** **Calibrating the RpoB and ribosomal proteins phylogenies**

We applied a set of twelve calibrations to the RpoB phylogeny as depicted in the image below (Part 1 and Part 2). Of these, eight had never been used before. A set of calibrations consisted of the earliest unambiguous evidence for Chroococcales Cyanobacteria of the Belcher group (point 21), the age of which has been recently revisited to 2.01 Ga [47]. This was assigned to the younger node from where Chroococcales strains branch out in the tree, with no maximum restrictions. The appearance of heterocystous Cyanobacteria were restricted from 0.72 Ga and 1.65 Ga as described in Supplementary Text S2. No constraints on the node representing the MRCA of Cyanobacteria were used. However, for rigor, we also tested an alternative single calibration on the node representing the MRCA of Cyanobacteria with a maximum of 2.01 Ga and no minimum, and with no other calibrations in the clade. This considered a scenario in which crown group Cyanobacteria are younger than the Belcher fossils.

In addition to cyanobacterial calibrations, we also applied the often-used biomarker evidence for phototrophic Chlorobia and Chromatiaceae at 1.64 Ga [48], see for example refs. [6, 49]. These were used as a minimum with no maximum constraints (node 30 and 31 in the image below, Part 2).

The new set of calibrations were chosen from well-described symbiotic relationships. Two of these are known in the phylum Margulisbacteria. The first one is *Termititenax*, these are specific ectosymbionts of spirochetes that live within oxymonad protists in the gut of diverse termites and cockroaches [50]. The two *Termititenax* sequences used in this analysis clustered together and therefore we calibrated this node to be between 396 Ma, some of the oldest fossil evidence of insects [51] and 140 Ma for *Mastotermes nepropadyom*, a Jurassic fossil termite [52] (node 22). This was done under the assumption that this symbiotic relationship may have started before the divergence of termites and cockroaches, as suggested in ref. [50]. The second one is *Candidatus* Ruthmannia eludens, a cell-type specific endosymbiont of placozoans, early-branching metazoans. This symbiont has been detected in all haplotypes examined, regardless of geographical location or sampling time [53, 54], which suggest that this association may be as old as placozoans. Therefore, we used a minimum calibration of 542 Ma as the Cambrian explosion of animal diversity and 660 Ma, the earliest biomarker evidence for desmosponges [55] (node 23), which should antedate the divergence of placozoans. This calibration was assigned to the node separating *Candidatus* Ruthmannia eludens from its closest sister sequence.

Similar to Margulisbacteria, members of the clade Vampirovibrionia have been reported to form close associations with eukaryotes. The clade Gastranaerophilales is thought to be composed mostly of strains that inhabit the animal gut. Thus, we calibrated the node separating two strains isolated from human and koala faeces that clustered together with a maximum age of 201 Ma, representing the Jurassic split of marsupials and placental mammals [56] (node 24a). However, the koala and human sequences were embedded in the Gastranaerophilales clade within other sequences from the human gut. Because of this, we trialled changing this calibration to 55 Ma instead, the oldest primate fossil [57] and assuming that the retrieved sequences from the human gut had a common ancestor younger than the MRCA of primates. Alternatively, we tested moving this calibration to the ancestral nodes of the clade that included all the human gut sequences (node 24b). Gastranaerophilales is closely related to the order Vampirovibrionales, which include *Vampirovibrio chlorellavorus*. This strain is a predator of the eukaryotic green algae *Chlorella* [58], and therefore we trialled a calibration assuming that Gastranaerophilales and Vampirovibrionales radiated after the MRCA of eukaryotes (node 25). We thus assigned a maximum calibration to this node of 1.8 Ga representing the earliest described plausible eukaryote fossils [31] and no minimum age.


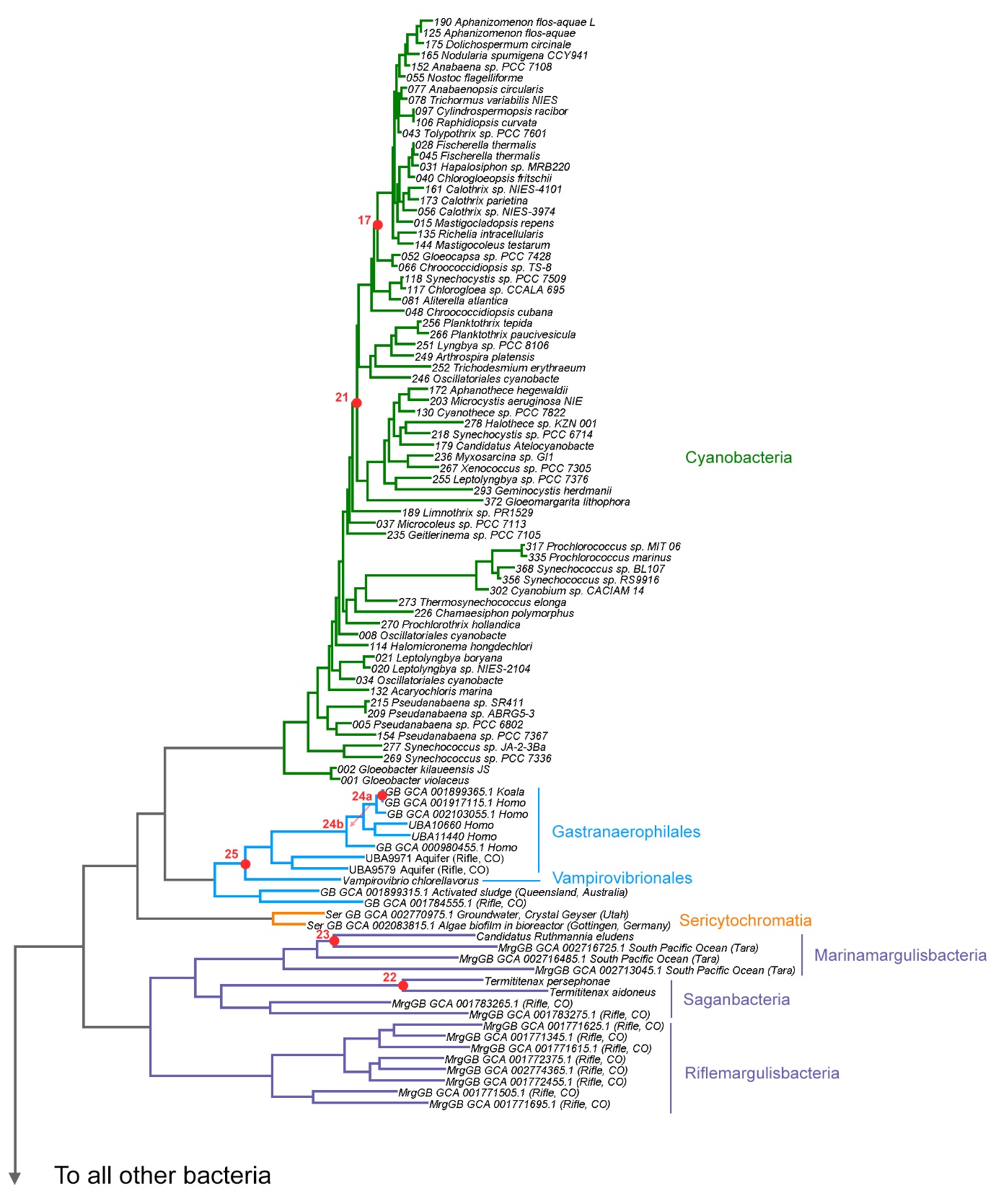


***Part 1.***


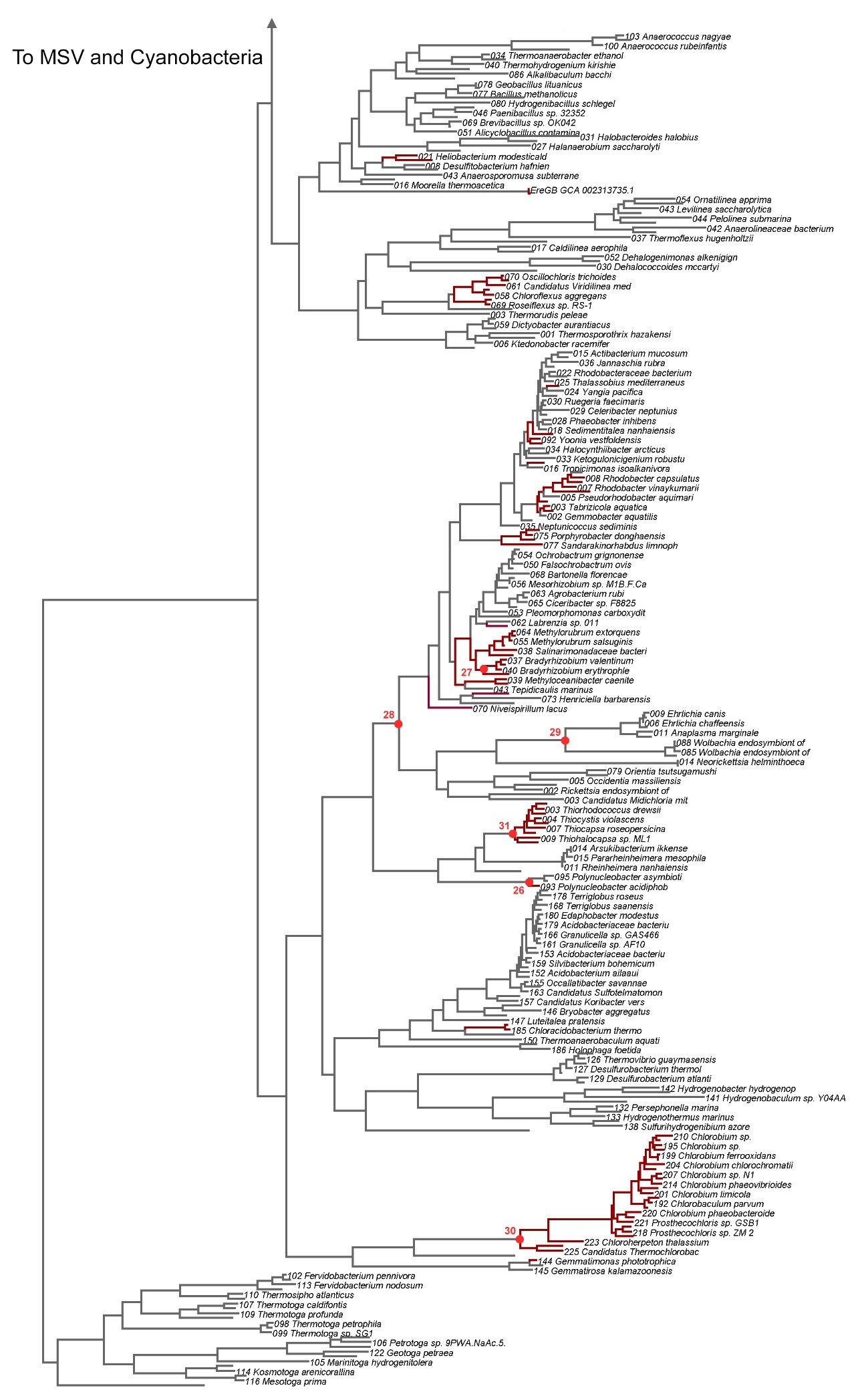


***Part 2***.

Another highly specific obligate symbiosis is that of the betaproteobacterium *Polynucleobacter necessarius* and ciliates of the genus *Euplotes* (Spirotrichea) [59]. *Polynucleobacter* has close free-living phototrophic relatives within the same genus [59]. We set the node separating the phototrophic and non-phototrophic *Polynucleobacter* (node 26) a maximum age of 444 Ma for the oldest fossil evidence of spirotrichs, as implemented in Parfrey et al. [60], and which predates the radiation of the genus *Euplotes* [61]*.*

Another well-known association is that of the soil bacteria *Bradyrhizobium* and legumes. Thus we gave the node separating *Bradyrhizobium* spp. from its closest relative in the RpoB tree, *Xanthobacter autotrophicus*, a maximum age of 86 Ma for Rosids, which contain legumes [29] (node 27).

The Rickettsiales are Alphaproteobacteria that exists in very close association with eukaryotes [62]. An association that may reach to the lineage leading to the origin of mitochondria [63]. Therefore, we assumed that the divergence of Rickettsiales occurred before the MRCA of eukaryotes and gave this node a minimum age of 1.8 Ga [31] (node 28). Finally, the family Anaplasmataceae contains bacteria that exists in close association with insects as endosymbionts (e.g. *Wolbachia*) or as parasite vectors (e.g. *Anaplasma*). Therefore, we set a maximum constraint for the MRCA of *Wolbachia* and *Anaplasma* (node 29)*,* excluding *Neorickettsia*, to be as old as the earliest evidence for insects about 395 Ma ago [51].

To constrain the age of the root of the RpoB tree, approximating the MRCA of Bacteria at the divergence of Thermotogae, we first set a broad gamma prior with an average of 3.8 Ga and a standard deviation of 0.5 Ga. We found this to perform well and used it as benchmark to compare with a range of evolutionary models and the effects of key calibrations (Supplementary Figure S8). Alternatively, we applied a broad calibration on the root with a maximum of 4.52 Ga, as described in above in Supplementary Text S2, and a minimum of 3.41 Ga, which is the earliest well-accepted evidence for photosynthesis [64]. This evidence was hypothesized to be anoxygenic in ref. [64]. Therefore, Bacteria should be at least as old as the earliest evidence for photosynthesis under conventional evolutionary scenarios.

To further understand the effect that a distant outgroup would have on the estimated divergence time, and to measure the rate of evolution of RpoB at the divergence of Bacteria and Archaea, we repeated the clock including 112 diverse archaeal sequences, in addition to Thermotogae, MSV, and Cyanobacteria, but removing all other clades as a compromise between robustness and computing time. Calibrations were assigned on MSV and Cyanobacteria as described above.

We compared the RpoB molecular clock that contained sequences from Archaea with that of a clock executed using a dataset of concatenated ribosomal proteins that included sequences from Cyanobacteria, Vampirovibrionia, Margulisbacteria, Thermotogae and a set of diverse sequences from Archaea. The tree was calibrating using point 17 and 21, a calibration on the MRCA of Gastranaerophilales with a maximum 201 Ma (point 24b), and a root calibration between 4.52 and 3.41 Ga, as illustrated in the figure below.


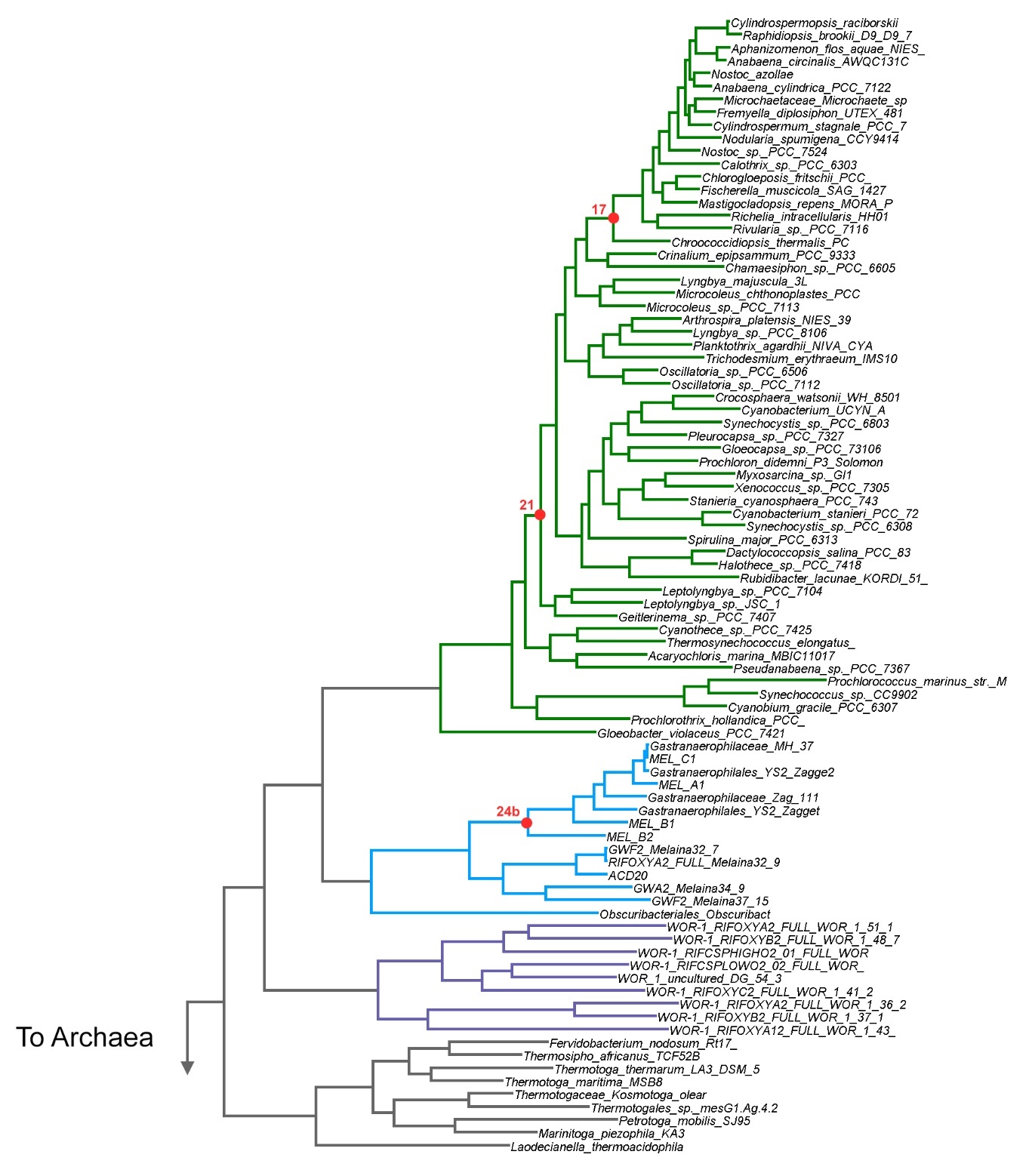


**References**

1. Cardona, T., *A fresh look at the evolution and diversification of photochemical reaction centers.* Photosynth. Res., 2015. **126**: 111-134. DOI: 10.1007/s11120-014-0065-x.

2. Cardona, T. and A.W. Rutherford, *Evolution of photochemical reaction centres: more twists?* Trends Plant Sci., 2019. **24**: 1008-1021. DOI: 10.1016/j.tplants.2019.06.016.

3. Mulkidjanian, A.Y., K.S. Makarova, M.Y. Galperin, and E.V. Koonin, *Inventing the dynamo machine: the evolution of the F-type and V-type ATPases.* Nat. Rev. Microbiol., 2007. **5**: 892-9. DOI: 10.1038/nrmicro1767.

4. Carnevali, P.B.M., F. Schulz, C.J. Castelle, R.S. Kantor, P.M. Shih, I. Sharon, J.M. Santini, M.R. Olm, Y. Amano, B.C. Thomas, K. Anantharaman, D. Burstein, E.D. Becraft, R. Stepanauskas, T. Woyke, and J.F. Banfield, *Hydrogen-based metabolism as an ancestral trait in lineages sibling to the Cyanobacteria.* Nat. Commun., 2019. **10**: 463. DOI: 10.1038/s41467-018-08246-y.

5. Ho, S.Y.W. and S. Duchene, *Molecular-clock methods for estimating evolutionary rates and timescales.* Mol. Ecol., 2014. **23**: 5947-5965. DOI: 10.1111/mec.12953.

6. David, L.A. and E.J. Alm, *Rapid evolutionary innovation during an Archaean genetic expansion.* Nature, 2011. **469**: 93-96. DOI: 10.1038/Nature09649.

7. Zhu, Q., U. Mai, W. Pfeiffer, S. Janssen, F. Asnicar, J.G. Sanders, P. Belda-Ferre, G.A. Al-Ghalith, E. Kopylova, D. McDonald, T. Kosciolek, J.B. Yin, S. Huang, N. Salam, J.-Y. Jiao, Z. Wu, Z.Z. Xu, K. Cantrell, Y. Yang, E. Sayyari, M. Rabiee, J.T. Morton, S. Podell, D. Knights, W.-J. Li, C. Huttenhower, N. Segata, L. Smarr, S. Mirarab, and R. Knight, *Phylogenomics of 10,575 genomes reveals evolutionary proximity between domains Bacteria and Archaea.* Nat. Commun., 2019. **10**: 5477. DOI: 10.1038/s41467-019-13443-4.

8. Betts, H.C., M.N. Puttick, J.W. Clark, T.A. Williams, P.C.J. Donoghue, and D. Pisani, *Integrated genomic and fossil evidence illuminates life's early evolution and eukaryote origin.* Nat. Ecol. Evol., 2018. **2**: 1556-1562. DOI: 10.1038/s41559-018-0644-x.

9. Yang, Z. and B. Rannala, *Bayesian estimation of species divergence times under a molecular clock using multiple fossil calibrations with soft bounds.* Mol. Biol. Evol., 2006. **23**: 212-26. DOI: 10.1093/molbev/msj024.

10. Cardona, T., P. Sánchez-Baracaldo, A.W. Rutherford, and A.W.D. Larkum, *Early Archean origin of Photosystem II.* Geobiology, 2019. **17**: 127-150. DOI: 10.1111/gbi.12322.

11. Cardona, T., J.W. Murray, and A.W. Rutherford, *Origin and evolution of water oxidation before the last common ancestor of the Cyanobacteria.* Mol. Biol. Evol., 2015. **32**: 1310-1328. DOI: 10.1093/molbev/msv024.

12. Ho, M.Y., G. Shen, D.P. Canniffe, C. Zhao, and D.A. Bryant, *Light-dependent chlorophyll f synthase is a highly divergent paralog of PsbA of Photosystem II.* Science, 2016. **353**: aaf9178. DOI: 10.1126/science.aaf9178.

13. Wegener, K.M., A. Nagarajan, and H.B. Pakrasi, *An atypical psbA gene encodes a sentinel D1 protein to form a physiologically relevant inactive Photosystem II complex in Cyanobacteria.* J. Biol. Chem., 2015. **290**: 3764-74. DOI: 10.1074/jbc.M114.604124.

14. Ago, H., H. Adachi, Y. Umena, T. Tashiro, K. Kawakami, N. Kamiya, L.R. Tian, G.Y. Han, T.Y. Kuang, Z.Y. Liu, F.J. Wang, H.F. Zou, I. Enami, M. Miyano, and J.R. Shen, *Novel features of eukaryotic Photosystem II revealed by its crystal structure analysis from a red alga.* J. Biol. Chem., 2016. **291**: 5676-5687. DOI: 10.1074/jbc.M115.711689.

15. Kimura, M. and T. Ota, *On the rate of molecular evolution.* J. Mol. Evol., 1971. **1**: 1-17. DOI: 10.1007/bf01659390.

16. Cardona, T., *Early Archean origin of heterodimeric Photosystem I.* Heliyon, 2018. **4**: e00548. DOI: 10.1016/j.heliyon.2018.e00548.

17. McLaughlin, J.P. and M.O. Dayhoff, *Evolution of species and proteins: A time scale*, in *Atlas of protein seqeunce and structure*, M.O. Dayhoff, Editor. 1972, National Biomedical Research Foundation: Washington D.C. 47-52.

18. Clarke, J.T., R.C.M. Warnock, and P.C.J. Donoghue, *Establishing a time-scale for plant evolution.* New Phytologist, 2011. **192**: 266-301. DOI: 10.1111/j.1469-8137.2011.03794.x.

19. Yang, E.C., S.M. Boo, D. Bhattacharya, G.W. Saunders, A.H. Knoll, S. Fredericq, L. Graf, and H.S. Yoon, *Divergence time estimates and the evolution of major lineages in the florideophyte red algae.* Sci Rep, 2016. **6**: 21361. DOI: 10.1038/srep21361.

20. Sánchez-Baracaldo, P., J.A. Raven, D. Pisani, and A.H. Knoll, *Early photosynthetic eukaryotes inhabited low-salinity habitats.* Proc. Natl. Acad. Sci. U.S.A., 2017. **114**: E7737-E7745. DOI: 10.1073/pnas.1620089114.

21. Butterfield, N.J., *Bangiomorpha pubescens n. gen., n. sp.: implications for the evolution of sex, multicellularity, and the Mesoproterozoic/Neoproterozoic radiation of eukaryotes.* Paleobiology, 2000. **26**: 386-404. DOI: 10.1666/0094-8373(2000)026<0386:Bpngns>2.0.Co;2.

22. Ponce-Toledo, R.I., P. Deschamps, P. Lopez-Garcia, Y. Zivanovic, K. Benzerara, and D. Moreira, *An early-branching freshwater cyanobacterium at the origin of plastids.* Current Biology, 2017. **27**: 386-391. DOI: 10.1016/j.cub.2016.11.056.

23. Fewer, D., T. Friedl, and B. Budel, *Chroococcidiopsis and heterocyst-differentiating cyanobacteria are each other's closest living relatives.* Mol. Phylogen. Evol., 2002. **23**: 82-90. DOI: 10.1006/mpev.2001.1075.

24. Shih, P.M., D. Wu, A. Latifi, S.D. Axen, D.P. Fewer, E. Talla, A. Calteau, F. Cai, N. Tandeau de Marsac, R. Rippka, M. Herdman, K. Sivonen, T. Coursin, T. Laurent, L. Goodwin, M. Nolan, K.W. Davenport, C.S. Han, E.M. Rubin, J.A. Eisen, T. Woyke, M. Gugger, and C.A. Kerfeld, *Improving the coverage of the cyanobacterial phylum using diversity-driven genome sequencing.* Proc. Natl. Acad. Sci. U.S.A., 2013. **110**: 1053-8. DOI: 10.1073/pnas.1217107110.

25. Schirrmeister, B.E., A. Antonelli, and H.C. Bagheri, *The origin of multicellularity in cyanobacteria.* BMC Evol. Biol., 2011. **11**: 45. DOI: 10.1186/1471-2148-11-45.

26. Ciccarelli, F.D., T. Doerks, C. von Mering, C.J. Creevey, B. Snel, and P. Bork, *Toward automatic reconstruction of a highly resolved tree of life.* Science, 2006. **311**: 1283-7. DOI: 10.1126/science.1123061.

27. Nakamura, Y., T. Kaneko, S. Sato, M. Mimuro, H. Miyashita, T. Tsuchiya, S. Sasamoto, A. Watanabe, K. Kawashima, Y. Kishida, C. Kiyokawa, M. Kohara, M. Matsumoto, A. Matsuno, N. Nakazaki, S. Shimpo, C. Takeuchi, M. Yamada, and S. Tabata, *Complete genome structure of Gloeobacter violaceus PCC 7421, a cyanobacterium that lacks thylakoids.* DNA Research, 2003. **10**: 137-145. DOI: 10.1093/dnares/10.4.137.

28. Honda, D., A. Yokota, and J. Sugiyama, *Detection of seven major evolutionary lineages in cyanobacteria based on the 16S rRNA gene sequence analysis with new sequences of five marine Synechococcus strains.* J. Mol. Biol., 1999. **48**: 723-739. DOI: 10.1007/Pl00006517.

29. Morris, J.L., M.N. Puttick, J.W. Clark, D. Edwards, P. Kenrick, S. Pressel, C.H. Wellman, Z.H. Yang, H. Schneider, and P.C.J. Donoghue, *The timescale of early land plant evolution.* Proc. Natl. Acad. Sci. U.S.A., 2018. **115**: E2274-E2283. DOI: 10.1073/pnas.1719588115.

30. Knoll, A.H., S. Worndle, and L.C. Kah, *Covariance of microfossil assemblages and microbialite textures across an upper mesoproterozoic carbonate platform.* Palaios, 2013. **28**: 453-470. DOI: 10.2110/palo.2013.p13-005r.

31. Knoll, A.H., E.J. Javaux, D. Hewitt, and P. Cohen, *Eukaryotic organisms in Proterozoic oceans.* ‎Philos. Trans. Royal Soc. B, 2006. **361**: 1023-1038. DOI: 10.1098/rstb.2006.1843.

32. Han, T.M. and B. Runnegar, *Megascopic eukaryotic algae from the 2.1-billion-year-old Negaunee iron-formation, Michigan.* Science, 1992. **257**: 232-235. DOI: 10.1126/science.1631544.

33. El Albani, A., M.G. Mangano, L.A. Buatois, S. Bengtson, A. Riboulleau, A. Bekker, K. Konhauser, T. Lyons, C. Rollion-Bard, O. Bankole, S.G.L. Baghekema, A. Meunier, A. Trentesaux, A. Mazurier, J. Aubineau, C. Laforest, C. Fontaine, P. Recourt, E.C. Fru, R. Macchiarelli, J.Y. Reynaud, F. Gauthier-Lafaye, and D.E. Canfield, *Organism motility in an oxygenated shallow-marine environment 2.1 billion years ago.* Proc. Natl. Acad. Sci. U.S.A., 2019. **116**: 3431-3436. DOI: 10.1073/pnas.1815721116.

34. Pang, K., Q. Tang, L. Chen, B. Wan, C. Niu, X. Yuan, and S. Xiao, *Nitrogen-fixing heterocystous cyanobacteria in the tonian period.* Current Biology, 2018. **28**: 616-622 e1. DOI: 10.1016/j.cub.2018.01.008.

35. Shi, M., Q.L. Feng, M.Z. Khan, and S.X. Zhu, *An eukaryote-bearing microbiota from the early mesoproterozoic Gaoyuzhuang Formation, Tianjin, China and its significance.* Precambrian Res., 2017. **303**: 709-726. DOI: 10.1016/j.precamres.2017.09.013.

36. Schirrmeister, B.E., P. Sánchez-Baracaldo, and D. Wacey, *Cyanobacterial evolution during the Precambrian.* Int. J. Astrobiol., 2016. **15**: 187-204. DOI: 10.1017/S1473550415000579.

37. Shih, P.M., J. Hemp, L.M. Ward, N.J. Matzke, and W.W. Fischer, *Crown group Oxyphotobacteria postdate the rise of oxygen.* Geobiology, 2017. **15**: 19-29. DOI: 10.1111/gbi.12200.

38. Sánchez-Baracaldo, P., *Origin of marine planktonic cyanobacteria.* Sci. Rep., 2015. **5**: 17418. DOI: 10.1038/srep17418.

39. Langklotz, S., U. Baumann, and F. Narberhaus, *Structure and function of the bacterial AAA protease FtsH.* Biochim Biophys Acta, 2012. **1823**: 40-8. DOI: 10.1016/j.bbamcr.2011.08.015.

40. Shao, S., T. Cardona, and P.J. Nixon, *Early emergence of the FtsH proteases involved in photosystem II repair.* Photosynthetica, 2018. **56**: 163-177. DOI: 10.1007/s11099-018-0769-9.

41. Lepage, T., D. Bryant, H. Philippe, and N. Lartillot, *A general comparison of relaxed molecular clock models.* Mol. Biol. Evol., 2007. **24**: 2669-80. DOI: 10.1093/molbev/msm193.

42. Kishino, H., J.L. Thorne, and W.J. Bruno, *Performance of a divergence time estimation method under a probabilistic model of rate evolution.* Mol. Biol. Evol., 2001. **18**: 352-361. DOI: 10.1093/oxfordjournals.molbev.a003811.

43. Medlin, L.K., W.H.C.F. Kooistra, R. Gersonde, P.A. Sims, and U. Wellbrock, *Is the origin of the diatoms related to the end-Permian mass extinction?* Nova Hedwigia, 1997. **65**: 1-11.

44. Barboni, M., P. Boehnke, B. Keller, I.E. Kohl, B. Schoene, E.D. Young, and K.D. McKeegan, *Early formation of the Moon 4.51 billion years ago.* Science Advances, 2017. **3**: e1602365. DOI: 10.1126/sciadv.1602365.

45. Homann, M., C. Heubeck, A. Airo, and M.M. Tice, *Morphological adaptations of 3.22 Ga-old tufted microbial mats to Archean coastal habitats (Moodies Group, Barberton Greenstone Belt, South Africa).* Precambrian Res., 2015. **266**: 47-64. DOI: 10.1016/j.precamres.2015.04.018.

46. Drummond, A.J., S.Y.W. Ho, M.J. Phillips, and A. Rambaut, *Relaxed phylogenetics and dating with confidence.* Plos Biology, 2006. **4**: 699-710. DOI: 10.1371/journal.pbio.0040088.

47. Hodgskiss, M.S.W., O.M.J. Dagnaud, J.L. Frost, G.P. Halverson, M.D. Schmitz, N.L. Swanson-Hysell, and E.A. Sperling, *New insights on the Orosirian carbon cycle, early Cyanobacteria, and the assembly of Laurentia from the Paleoproterozoic Belcher Group.* Earth Planet. Sci. Lett., 2019. **520**: 141-152. DOI: 10.1016/j.epsl.2019.05.023.

48. Brocks, J.J., G.D. Love, R.E. Summons, A.H. Knoll, G.A. Logan, and S.A. Bowden, *Biomarker evidence for green and purple sulphur bacteria in a stratified Palaeoproterozoic sea.* Nature, 2005. **437**: 866-870. DOI: 10.1038/Nature04068.

49. Magnabosco, C., K.R. Moore, J.M. Wolfe, and G.P. Fournier, *Dating phototrophic microbial lineages with reticulate gene histories.* Geobiology, 2018. **16**: 179-189. DOI: 10.1111/gbi.12273.

50. Utami, Y.D., H. Kuwahara, K. Igai, T. Murakami, K. Sugaya, T. Morikawa, Y. Nagura, M. Yuki, P. Deevong, T. Inoue, K. Kihara, N. Lo, A. Yamada, M. Ohkuma, and Y. Hongoh, *Genome analyses of uncultured TG2/ZB3 bacteria in 'Margulisbacteria' specifically attached to ectosymbiotic spirochetes of protists in the termite gut.* ISME J., 2019. **13**: 455-467. DOI: 10.1038/s41396-018-0297-4.

51. Engel, M.S. and D.A. Grimaldi, *New light shed on the oldest insect.* Nature, 2004. **427**: 627-630. DOI: 10.1038/nature02291.

52. Legendre, F., A. Nel, G.J. Svenson, T. Robillard, R. Pellens, and P. Grandcolas, *Phylogeny of Dictyoptera: Dating the origin of cockroaches, praying mantises and termites with molecular data and controlled fossil evidence.* PloS one, 2015. **10**: e0130127. DOI: 10.1371/journal.pone.0130127.

53. Gruber-Vodicka, H.R., N. Leisch, M. Kleiner, T. Hinzke, M. Liebeke, M. McFall-Ngai, M.G. Hadfield, and N. Dubilier, *Two intracellular and cell type-specific bacterial symbionts in the placozoan Trichoplax H2.* Nat. Microbiol., 2019. **4**: 1465-1474. DOI: 10.1038/s41564-019-0475-9.

54. Eitel, M., H.J. Osigus, R. DeSalle, and B. Schierwater, *Global diversity of the Placozoa.* PloS one, 2013. **8**: e57131. DOI: 10.1371/journal.pone.0057131.

55. Zumberge, J.A., G.D. Love, P. Cardenas, E.A. Sperling, S. Gunasekera, M. Rohrssen, E. Grosjean, J.P. Grotzinger, and R.E. Summons, *Demosponge steroid biomarker 26-methylstigmastane provides evidence for Neoproterozoic animals.* Nat. Ecol. Evol., 2018. **2**: 1709-1714. DOI: 10.1038/s41559-018-0676-2.

56. Grossnickle, D.M., S.M. Smith, and G.P. Wilson, *Untangling the multiple ecological radiations of early mammals.* Trends in Ecology & Evolution, 2019. **34**: 936-949. DOI: 10.1016/j.tree.2019.05.008.

57. Williams, B.A., R.F. Kay, and E.C. Kirk, *New perspectives on anthropoid origins.* Proc. Natl. Acad. Sci. U.S.A., 2010. **107**: 4797-804. DOI: 10.1073/pnas.0908320107.

58. Soo, R.M., B.J. Woodcroft, D.H. Parks, G.W. Tyson, and P. Hugenholtz, *Back from the dead; the curious tale of the predatory cyanobacterium Vampirovibrio chlorellavorus.* PeerJ, 2015. **3**: e968. DOI: 10.7717/peerj.968.

59. Hahn, M.W., J. Schmidt, A. Pitt, S.J. Taipale, and E. Lang, *Reclassification of four Polynucleobacter necessarius strains as representatives of Polynucleobacter asymbioticus comb. nov., Polynucleobacter duraquae sp. nov., Polynucleobacter yangtzensis sp. nov and Polynucleobacter sinensis sp.s nov., and emended description of Polynucleobacter necessarius.* Int. J. Syst. Evol. Microbiol., 2016. **66**: 2883-2892. DOI: 10.1099/ijsem.0.001073.

60. Parfrey, L.W., D.J. Lahr, A.H. Knoll, and L.A. Katz, *Estimating the timing of early eukaryotic diversification with multigene molecular clocks.* Proc. Natl. Acad. Sci. U.S.A., 2011. **108**: 13624-9. DOI: 10.1073/pnas.1110633108.

61. Fernandes, N.M. and C.G. Schrago, *A multigene timescale and diversification dynamics of Ciliophora evolution.* Mol. Phylogen. Evol., 2019. **139**: 106521. DOI: 10.1016/j.ympev.2019.106521.

62. Yu, X.-J. and D.H. Walker, *The Order Rickettsiales*, in *The prokaryotes: A handbook on the biology of bacteria*, M. Dworkin, Editor. 2006, Springer: Singapure. 493-528. DOI: 10.1007/0-387-30745-1_20.

63. Roger, A.J., S.A. Munoz-Gomez, and R. Kamikawa, *The Origin and Diversification of Mitochondria.* Curr Biol, 2017. **27**: R1177-R1192. DOI: 10.1016/j.cub.2017.09.015.

64. Tice, M.M. and D.R. Lowe, *Photosynthetic microbial mats in the 3,416-Myr-old ocean.* Nature, 2004. **431**: 549-52. DOI: 10.1038/nature02888.
